# Super-resolution mapping of nuclear stress signaling by probiotic-derived extracellular vesicles in UV-induced skin photodamage

**DOI:** 10.1126/sciadv.aek5197

**Published:** 2026-10-30

**Authors:** Ga-eun Go, Seungju Han, Minjae Kang, Sangoh Choi, Soo-eun Lee, Uidon Jeong, Sang-Hee Lee, Ki-Nam Gu, Hanji Kim, Bo Kyoung Hwang, Yunkwan Kim, Euitaek Jeong, Nae-Gyu Kang, Doory Kim

**Affiliations:** ^1^ Department of Chemistry, Hanyang University, Seoul 04763, Republic of Korea.; ^2^ Center for Bio-imaging & Translational Research, Korea Basic Science Institute, Cheongju 28119, Republic of Korea.; ^3^ R&D Institute, LG Household & Health Care (LG H&H), Seoul 07795, Republic of Korea.; ^4^ Research Institute for Convergence of Basic Science, Hanyang University, Seoul 04763, Republic of Korea.; ^5^ Institute of Nano Science and Technology, Hanyang University, Seoul 04763, Republic of Korea.; ^6^ Research Institute for Natural Sciences, Hanyang University, Seoul 04763, Republic of Korea.

## Abstract

Ultraviolet (UV)-induced photodamage drives oxidative stress, DNA damage, and chronic inflammation, leading to progressive cellular ultrastructure and skin function degeneration. Despite the potential of probiotics as anti-photodamage agents, their nanoscale action mechanism remains unexplored. Here, we utilize super-resolution fluorescence microscopy to investigate the cellular and molecular basis of *Lactobacillus plantarum*-derived extracellular vesicles (*L. plantarum* EVs) in mitigating photodamage. We showed that UV irradiation triggered extensive organellar remodeling along with elevated reactive oxygen species production, DNA damage signaling, and inflammatory activation. Remarkably, pre- and posttreatment with *L. plantarum* EVs preserved organelle integrity and restored native nanoscale architecture. Additionally, EV treatment delayed UV-induced PARP-1 activation and nuclear condensate clustering, while suppressing NF-κB translocation and downstream inflammatory cytokine expression. These findings establish probiotic-derived EVs as modulators of stress-responsive signaling and structural homeostasis during UV-induced photodamage. By providing a super-resolution mechanistic framework for understanding probiotic EV-mediated modulation of cellular photodamage responses, this study provides a basis for future investigation of EV-based strategies for skin protection.

## INTRODUCTION

Aging is a complex biological process characterized by progressive physiological decline and increased vulnerability to environmental stressors. Recently, substantial research efforts have focused on identifying the molecular mechanisms and interventions capable of delaying or reversing age-associated cellular dysfunction. Among the age-related changes, extrinsic aging caused by environmental oxidative factors such as solar radiation (*1*), cigarette smoke (*2*), and pollution is a significant concern in dermatological and cosmetic research (*3*). Photoaging, largely driven by ultraviolet (UV) radiation, degrades essential skin components such as collagen and elastin, amplifying wrinkles, hyperpigmentation, and loss of elasticity (*4*). Consequently, the development of effective strategies to mitigate or reverse photoaging has become an objective of anti-aging research.

Probiotics, particularly *Lactobacillus* and *Bifidobacterium* strains, have gained considerable attention for their anti-inflammatory (*5*), immunomodulatory, and skin-protective properties (*6*). Accumulating evidence suggests that probiotics exert anti-photoaging effects by reducing UV-induced oxidative stress and inflammation, thereby preserving collagen integrity and skin elasticity (*7*–*11*). While probiotic-derived conditioned media also demonstrate protection against UV-induced oxidative stress, hyperpigmentation, and collagen loss (*12*), these benefits are increasingly attributed to secreted extracellular vesicles (EVs). Bacterial EVs are emerging as pivotal mediators of microbe–host communication (*13*–*15*). These nanosized particles transport bioactive proteins, lipids, and metabolites that regulate skin cell homeostasis and protect against UV-induced photodamage (*16*, *17*). Consequently, understanding how probiotic EVs modulate cellular responses to UV-induced damage may provide new mechanistic insight into microbiome-mediated skin protection.

However, the current study has notable limitations. Many studies have focused on live probiotic applications without characterizing the distinct effects of bacterial EVs, making it difficult to clarify the exact mechanisms underlying the EV-mediated delivery of key molecules. In addition, standardized models for evaluating probiotic-derived EVs under UV-induced photodamage conditions are sparse, hindering direct comparison and reproducibility of the findings. To build on existing knowledge, more rigorous and targeted investigations are required, particularly those examining the precise molecular and cellular pathways through which *Lactobacillus plantarum* (*L. plantarum*) EVs exert protective or restorative effects on photodamaged skin cells.

In this study, we aimed to overcome these challenges by establishing a UV-induced photodamage model in human skin cells and evaluating the preventive and restorative potential of *L. plantarum* EVs using time-resolved super-resolution fluorescence microscopy. By integrating nanoscale imaging with quantitative spatiotemporal analysis, we directly visualize the dynamic molecular reorganization of key DNA damage response factors during UV-induced photostress. In particular, we uncover a previously unrecognized mechanism by which probiotic EVs modulate the condensate-like assembly kinetics of PARP-1, which is a phase separation–driven nuclear organization process that coordinates DNA damage sensing and downstream inflammatory signaling. Our work provides the first nanoscale evidence that bacterial EVs can kinetically tune stress-responsive biomolecular condensates in host cells, thereby reshaping the early molecular trajectory of photodamage. Together, these findings establish a new mechanistic framework for probiotic EV–mediated skin protection and highlight time-resolved super-resolution imaging as a powerful platform for decoding dynamic molecular processes underlying aging and inflammation.

## RESULTS

### UV-induced ultrastructural remodeling of cellular organelles revealed by super-resolution microscopy

We first aimed to establish a reliable UV-induced photodamage model by examining the ultrastructural changes in cellular organelles, as this is essential for accurately mimicking the early cellular events of photodamage and for detecting and quantifying the subtle remodeling associated with skin aging. As these subtle changes are likely obscured by the resolution limits of conventional optical microscopy, we attempted to visualize them using super-resolution stochastic optical reconstruction microscopy (STORM) imaging (Fig. 1). As UV illumination is expected to induce ultrastructural changes in key organelles, we exposed human dermal fibroblast (HDF) to varying UV intensities from 3 to 7 J/cm^2^ (*18*, *19*) and performed STORM imaging of the mitochondria, endoplasmic reticulum (ER), Golgi apparatus, microtubules, and microtubule organizing center (MTOC). Cell viability measured by Trypan blue exclusion assay confirmed that cells irradiated at 5 J/cm^2^ maintained high viability comparable to that of untreated controls (fig. S1), indicating that our UV exposure conditions induce cellular stress responses without triggering significant cell death.

**Fig. 1.**
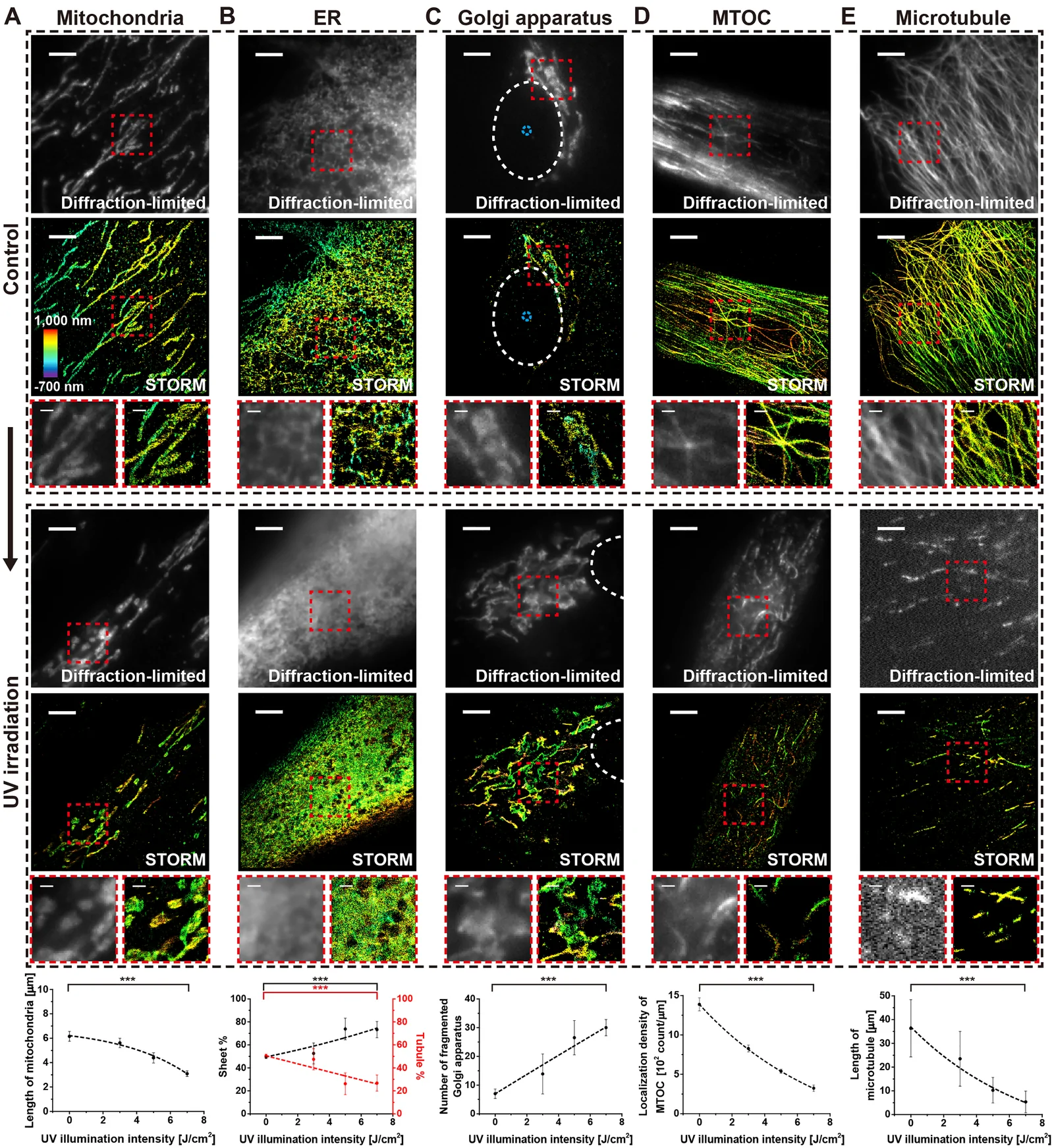
STORM imaging and quantification of organelle ultrastructural changes in human dermal fibroblast (HDF) cells under varying UV illumination intensities. Representative STORM images of (**A**) mitochondria, (**B**) endoplasmic reticulum (ER), (**C**) Golgi apparatus, (**D**) microtubule organizing center (MTOC), and (**E**) microtubules in control (no UV illumination) and UV-illuminated HDF cells are shown at the top and middle, respectively. Diffraction-limited images for the corresponding region of interest are shown together for a comparison. Corresponding quantitative analyses are shown at the bottom: (A) mitochondrial length, (B) ratio of tubular or sheet-like ER structures, (C) number of Golgi fragments, (D) localization density of MTOC, and (E) microtubule length. White dashed line: nuclear boundary. Scale bars, 5 μm and 1 μm in red dashed box. Data are presented as mean ± SEM from twelve independent experiments. One-way ANOVA with Tukey’s post hoc test, ***P < 0.001 compared to the control.

We observed the resolved mitochondrial structures in HDF and the resulting ultrastructural changes due to UV photodamage, as shown in Fig. 1A. The mitochondria in HDF exhibited an elongated morphology; however, with increasing UV illumination intensity, we observed progressive shortening of the mitochondria, implying mitochondrial fission. STORM imaging of the ER revealed two morphologies, tubular and sheet-like structures, which were present in roughly equal proportions in normal HDF. Upon exposure to higher UV intensities, the tubular ER structures progressively converted into sheet-like forms, reaching 73% at an intensity of 7 J/cm^2^ (Fig. 1B). Imaging of the Golgi apparatus revealed that strong UV exposure induced Golgi fragmentation and dispersion away from the nucleus (Fig. 1C, fig. S2). Because the Golgi apparatus is closely associated with the microtubule network and MTOC, we also performed STORM imaging of the MTOC. As shown in Fig. 1D, we observed that strong UV illumination induced MTOC depolymerization. Given that disruption of the microtubule network causes Golgi fragmentation and dispersal in diseased or stressed cells, our observations suggest that UV-induced Golgi fragmentation is a likely consequence of microtubule depolymerization. In this context, we performed STORM imaging of microtubules in UV-photodamaged HDF. STORM imaging of microtubules in UV-exposed cells revealed that higher UV intensities led to the severing and disruption of microtubules (Fig. 1E). As MTOC depolymerization destabilizes the foundational microtubule network, these broken microtubules likely result from MTOC depolymerization. Given that the microtubule network maintains the positioning and structural integrity of the Golgi apparatus and other organelles, disrupted microtubules may contribute to the fragmentation, dispersal, and structural alterations of the Golgi apparatus and ER observed under UV stress. These phenomena, including mitochondrial fission, ER transition from tubular to sheet-like structures, Golgi fragmentation and dispersion, and depolymerization of MTOC, have also been reported in cells infected with *Staphylococcus aureus* (*S. aureus*) EVs (*17*). To distinguish direct UV-induced organelle remodeling from secondary effects of cytoskeletal disruption, we compared UV irradiation with nocodazole-induced microtubule depolymerization (fig. S3). Although nocodazole treatment effectively disrupted the microtubule network to a similar extent, it failed to reproduce the mitochondrial fission, ER remodeling, and extensive Golgi fragmentation induced by UV irradiation. These results indicate that microtubule disruption alone cannot explain the widespread organelle remodeling observed under UV stress. Therefore, we interpreted these UV-induced organelle alterations as cellular responses to harmful stress, indicating organelle damage.

### *L. plantarum*-derived extracellular vesicle-mediated protection and restoration of cellular organelle ultrastructure in UV-damaged cells

Given that UV-induced photodamage leads to profound ultrastructural alterations in major cellular organelles, we considered these alterations reliable indicators of cellular conditions under stress. Given that probiotics have been reported to alleviate UV-induced oxidative stress and inflammation, we investigated whether EVs derived from the representative probiotic *L. plantarum* could restore the ultrastructural integrity of UV-photodamaged organelles. To test this hypothesis, we applied *L. plantarum* EVs to HDF and performed super-resolution STORM imaging to assess their therapeutic and protective effects.

To evaluate the therapeutic potential of *L. plantarum* EVs, we first applied EVs at varying concentrations and incubation times (*20*) and performed STORM imaging of the mitochondria, ER, Golgi apparatus, MTOC, and microtubules. Remarkably, we observed progressive recovery of UV-induced ultrastructural alterations, including mitochondrial fission, ER transition from tubular to sheet-like morphology, Golgi fragmentation and dispersal, MTOC depolymerization, and microtubule breakage (Fig. 2, A to E). The extent of recovery increased with both EV dose and treatment duration, indicating that *L. plantarum* EVs restored organelle integrity following UV-induced photodamage.

**Fig. 2.**
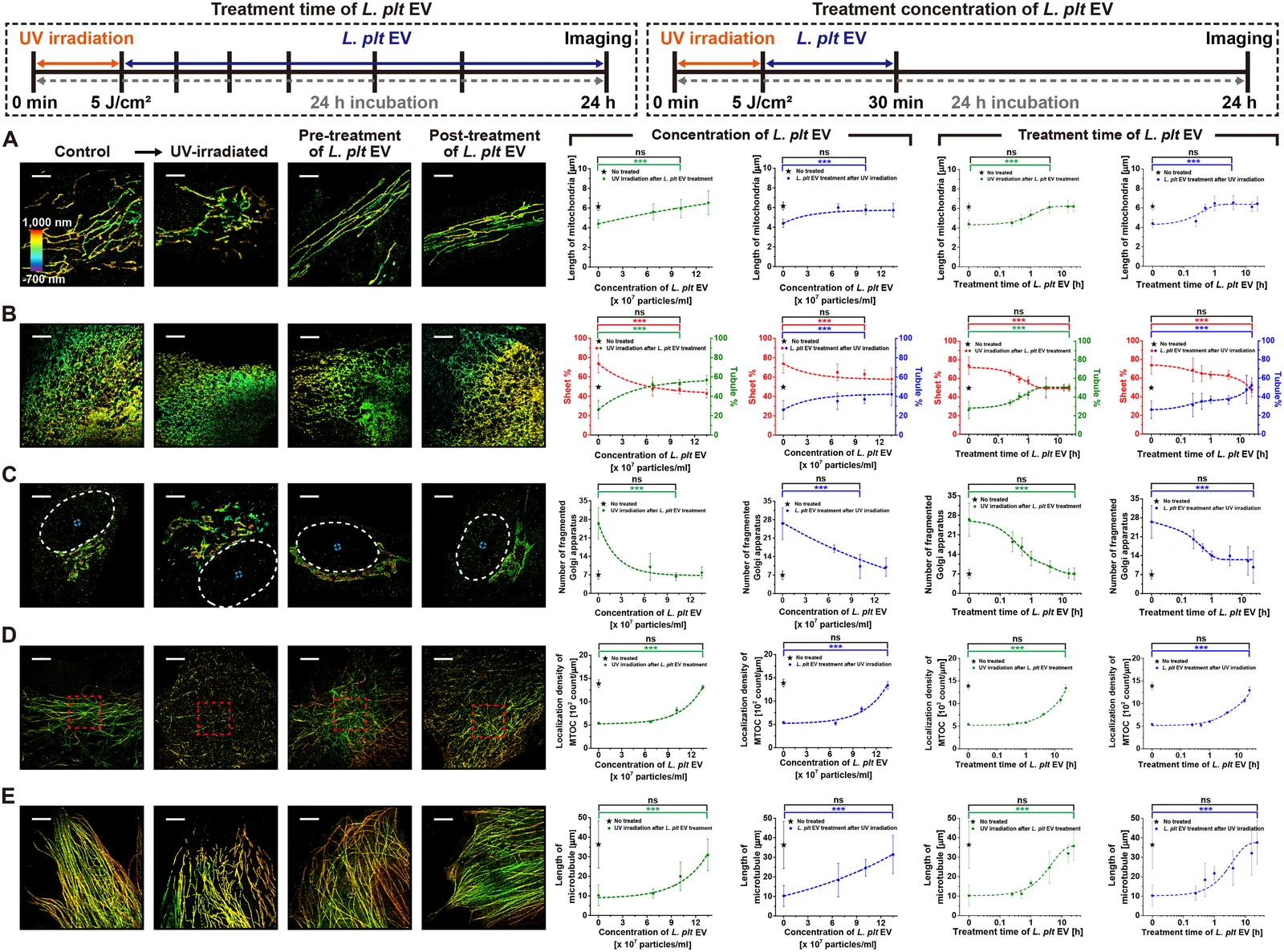
Protective and restorative effects of *Lactobacillus plantarum* extracellular vesicles (*L. plantarum* EVs) on UV-damaged organelles in HDF cells, as revealed by STORM imaging. Representative STORM images of (**A**) mitochondria, (**B**) endoplasmic reticulum (ER), (**C**) Golgi apparatus, (**D**) microtubule organizing center (MTOC), and (**E**) microtubules under four experimental conditions: untreated control, UV-damaged cells, cells pretreated with *L. plantarum* EVs before UV exposure, and cells treated with *L. plantarum* EVs after UV damage. Quantitative analyses of organelle morphology as a function of *L. plantarum* EV concentration and treatment duration are presented in the graphs on the right: (A) mitochondrial length, (B) relative ratio of tubular or sheet-like ER structure, (C) number of Golgi fragments, (D) localization density of MTOC, and (E) microtubule length. Trend lines were fitted using an exponential fitting model and are intended solely to guide the eye and illustrate the overall dose- and time-dependent recovery trends. White dashed line: nuclear boundary. Red dashed line: MTOC. Scale bars, 5 μm. Data are presented as mean ± SEM from twelve independent experiments. One-way ANOVA with Tukey’s post hoc test, ns: *P >* 0.05, ***P < 0.001 compared to the control.

Next, to determine whether *L. plantarum* EVs exhibit a protective effect against UV-induced photodamage, HDFs were pretreated with EVs at various concentrations and durations before UV exposure. The characteristic phenomena of UV-induced damage, including mitochondrial fission, ER remodeling, Golgi fragmentation, MTOC depolymerization, and microtubule breakage, were largely absent in EV-pretreated cells. Cells pretreated with higher EV concentrations and longer incubation times maintained organelle morphologies comparable to those of the untreated controls, even after UV illumination (Fig. 2, A to E, fig. S2). Specifically, treatment with 6.75 × 10^7^ particles/mL of EVs for 16 hours was sufficient to preserve organelle ultrastructure at the control level. These findings suggest that *L. plantarum* EVs can restore UV-induced organelle alterations and provide a protective shield against subsequent photodamage. To further support these findings at the ultrastructural level, transmission electron microscopy (TEM) imaging of mitochondria was performed as a representative organelle, given that mitochondrial morphology provides a particularly clear and interpretable readout of UV-induced structural damage under TEM. UV irradiation induced mitochondrial shortening and loss of internal cristae, while posttreatment with *L. plantarum* EVs restored mitochondrial length and cristae density to levels comparable to those of untreated controls (fig. S4).

To determine whether the protective effects of *L. plantarum* EVs extend beyond the preservation of intracellular organelle architecture, we next examined type I collagen, a major extracellular matrix component produced by dermal fibroblasts. STORM imaging revealed that UV irradiation markedly reduced intracellular collagen I abundance and extracellular collagen fibril density. In contrast, both pre- and posttreatment with *L. plantarum* EVs substantially restored collagen I following UV exposure (fig. S5A). Consistent with the STORM imaging results, Western blot analysis independently confirmed the UV-induced reduction in collagen I and its recovery following EV treatment (fig. S5B). These findings indicate that the protective effects of *L. plantarum* EVs extend beyond intracellular structural preservation to the maintenance of a major dermal extracellular matrix component.

To determine whether these protective effects represent a general response to bacterial EVs, we further examined EVs derived from *Staphylococcus epidermidis* (*S. epidermidis*) as an unrelated bacterial EV control. In contrast to *L. plantarum* EVs, *S. epidermidis* EVs showed little or no protective effect against UV-induced mitochondrial fragmentation and failed to delay the UV-induced peak in PARP-1 clustering or suppress NF-κB nuclear translocation (fig. S6). These results indicate that the protective effects observed with *L. plantarum* EVs are not a nonspecific response to bacterial EV exposure.

Interestingly, when we tested *L. plantarum*-derived nanoparticles (LDNPs) produced by high-pressure homogenization and applied them to UV-stressed HDFs, we found that LDNPs could recapitulate the bioactivity of naturally secreted EVs. As shown in fig. S7, LDNP treatment effectively restored the ultrastructural integrity of mitochondria, ER, and the Golgi apparatus, comparable to the effects of native EVs, as confirmed by STORM imaging. It suggests that LDNPs recapitulate the functional effects of endogenous *L. plantarum* EVs by incorporating critical bioactive components of *L. plantarum*.

To confirm that the observed biological effects are attributable to cellular uptake of *L. plantarum* EVs, we performed confocal and STORM imaging of CFSE-labeled EVs. Both imaging analyses revealed association of labeled EVs with the plasma membrane followed by a time-dependent increase in intracellular fluorescence, indicating cellular internalization of EV-associated components. Confocal z-sectioning and STORM imaging further confirmed that the fluorescent signals were localized within the interior of the cells across multiple z-planes, rather than remaining at the cell surface. Dye-only control experiments confirmed that the observed signal was not attributable to residual free dye (fig. S8). Together, these results establish that *L. plantarum* EVs are genuinely internalized by HDF cells, providing a cellular basis for the downstream modulation of intracellular signaling described below.

To identify a functional component underlying the observed EV-mediated effects, we next focused on NAD^+^ as the most likely candidate mediator among the diverse bioactive constituents of *L. plantarum* EVs, which are known to include bacterial cytoplasmic membrane constituents, fatty acids, and various amino and nucleic acids. NAD^+^ emerged as the most functionally relevant candidate given its well-documented anti-inflammatory properties and its established role in regulating cellular stress responses. NAD^+^ has been previously detected in *L. plantarum* EV preparations by LC-MS and quantified at 0.55 μg/mL at the EV concentration used in this study (6.75 × 10^7^ particles/mL) (*20*), confirming its presence as a genuine constituent of *L. plantarum* EVs. Notably, our previous quantitative analysis also detected NAD^+^ in *S. epidermidis* EVs, but at approximately half the level measured in *L. plantarum* EVs (*20*), suggesting that differences in EV-associated NAD^+^ abundance may contribute, at least in part, to their differential biological activities. To determine whether NAD^+^ alone is sufficient to recapitulate the EV-mediated effects, NAD^+^-loaded liposomes were applied to UV-photodamaged HDF cells, and intracellular NAD^+^ levels were quantified following EV pre- and posttreatment. Both treatment conditions significantly elevated intracellular NAD^+^ levels and restored UV-induced organelle damage in a concentration- and time-dependent manner closely mirroring that observed with *L. plantarum* EV treatment (fig. S9). To further confirm that the observed restorative effects are attributable to the bioactive cargo of *L. plantarum* EVs rather than to the lipid vesicle carrier itself, we applied NAD^+^-free empty liposomes to UV-photodamaged HDF cells under the same pre- and posttreatment conditions. As shown in fig. S10, empty liposome treatment did not restore UV-induced organelle damage, with cells continuing to exhibit mitochondrial fission, sheet-like ER structures, and fragmented and dispersed Golgi. These results confirm that the organelle-protective effects observed with *L. plantarum* EVs arise specifically from their bioactive constituents rather than from a nonspecific effect of lipid-based vesicles.

To further determine whether the protective effects of *L. plantarum* EVs depend on cellular NAD^+^ availability, we depleted intracellular NAD^+^ using FK866, an inhibitor of NAMPT-mediated NAD^+^ biosynthesis. As shown in fig. S11, FK866 pretreatment largely abolished the EV-mediated restoration of intracellular NAD^+^ levels following UV irradiation. Moreover, NAD^+^ depletion abolished the EV-induced delay in PARP-1 clustering, shifting the peak from 6 hours back to 3 hours, and substantially impaired the EV-mediated restoration of UV-induced mitochondrial fragmentation (fig. S11). Together with the ability of NAD^+^-loaded liposomes to recapitulate the effects of native EVs, these loss-of-function results further support cellular NAD^+^ as an important functional mediator of *L. plantarum* EV activity.

Taken together, the gain-of-function experiments using NAD^+^-loaded liposomes and the loss-of-function experiments using FK866-mediated NAD^+^ depletion support cellular NAD^+^ as an important functional mediator of the protective effects of *L. plantarum* EVs, although contributions from other EV-associated components cannot be excluded.

Finally, to further evaluate whether the anti-photodamage effects of *L. plantarum* EVs observed in 2D cell culture are preserved in a more physiologically relevant system, we performed additional experiments using a reconstructed human skin model. Because the thickness of the reconstructed tissue precluded STORM imaging, we performed confocal microscopy and TEM instead (fig. S12). Confocal imaging demonstrated that UV irradiation induced mitochondrial fission, as evidenced by decreased mitochondrial length, increased circularity, and decreased aspect ratio, together with microtubule depolymerization, all of which were largely restored by both pre- and posttreatment with *L. plantarum* EVs. Furthermore, TEM imaging of reconstructed skin sections confirmed UV-induced mitochondrial fission with cristae loss, ER lumen swelling and dilation, as well as heterochromatin condensation and nuclear membrane invagination, all of which were substantially reversed following EV treatment. These findings demonstrate that the protective effects of *L. plantarum* EVs observed in HDF monolayers are also reproducible in a physiologically more relevant reconstructed skin model, extending our observations beyond conventional two-dimensional cell culture.

### Mechanistic insights into UV-induced photodamage at the cellular and molecular levels

Our results demonstrated that UV-induced photodamage disrupts the ultrastructural integrity of major cellular organelles and that these alterations can be restored or prevented by treatment with *L. plantarum*-derived EVs. To further elucidate the underlying basis of these phenomena, we investigated the mechanisms driving UV-induced organelle remodeling at the cellular and molecular levels.

As UV irradiation induces the generation of reactive oxygen species (ROS), which trigger diverse cellular responses, we first assessed whether sufficient ROS were produced under our experimental conditions to account for the observed organelle alterations. Thus, we quantified intracellular ROS levels in HDF at various time points after UV illumination to establish the temporal sequence of molecular and cellular events. Intracellular ROS levels were quantified using 2′,7′- dichlorodihydrofluorescein diacetate (DCFH-DA), and the fluorescence intensity was measured as an indicator of ROS generation. As shown in Fig. 3A and fig. S13, intracellular ROS generation increased after UV illumination, peaked at 15 min postirradiation, and decreased. These results confirmed that our UV exposure conditions were sufficient to elicit robust ROS production. To understand the dose-dependent oxidative response, intracellular ROS levels were quantified in HDF at approximately 15 min after exposure to increasing UV intensities. As shown in Fig. 3A, the ROS levels increased progressively with UV intensity, confirming robust oxidative stress under UV exposure.

**Fig. 3.**
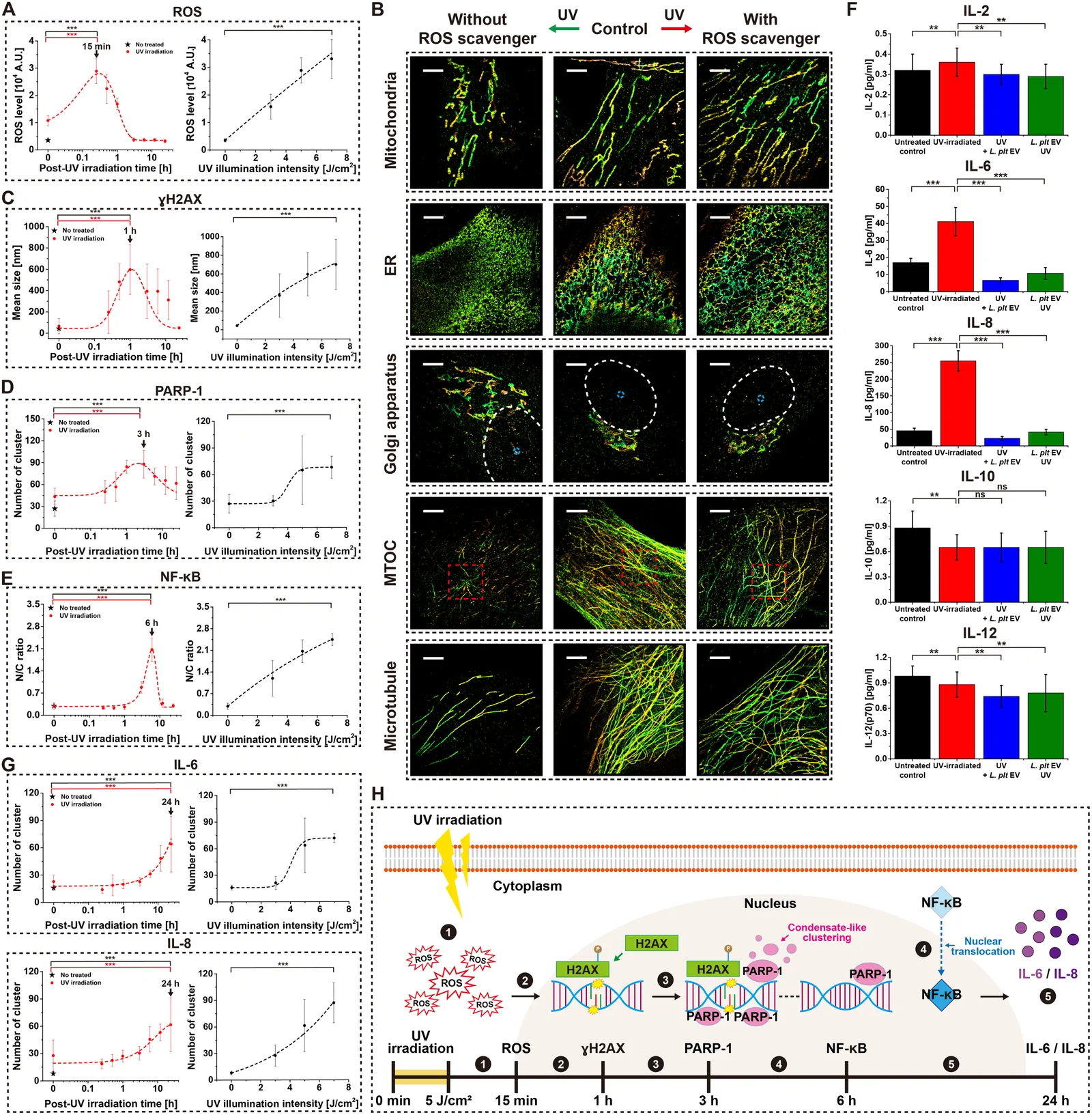
Molecular mechanisms driving UV-induced organelle remodeling. (**A**) Quantification of intracellular ROS levels measured using DCFH-DA fluorescence in HDF cells after UV irradiation. Left: Time course of ROS generation at different postirradiation time points. Right: Dependence of ROS generation on UV illumination intensity, measured 15 min after exposure. (**B**) Representative STORM images of mitochondria, endoplasmic reticulum (ER), Golgi apparatus, microtubule organizing center (MTOC), and microtubules in UV-irradiated HDF with or without ascorbic acid treatment as an ROS scavenger. White dashed line: nuclear boundary. Red dashed line: MTOC. (**C** and **D**) Quantitative analysis of (C) γH2AX and (D) PARP-1 in HDF cells after UV irradiation. Left: Time course of γH2AX size (C) and number of PARP-1 clusters (D) at different postirradiation time points. Right: Dependence of γH2AX size (C) and number of PARP-1 clusters (D) on UV illumination intensity, measured 1 hour after exposure. (**E**) Quantification of the temporal dynamics (left) and UV illumination intensity dependence of NF-κB nuclear translocation in HDF cells following UV irradiation, expressed as the ratio of nuclear to cytosolic localization based on STORM imaging. (**F**) Cytokine levels (IL-2, IL-6, IL-8, IL-10, and IL-12) in HDF cells under four conditions: untreated control, UV-irradiated, and UV-irradiated with either pre- or posttreatment with *L. plantarum* EVs. (**G**) Temporal evolution of IL-6 and IL-8 nanoscale clustering in HDF cells after UV irradiation, as determined by STORM. Trend lines in (A), (C), (D), (E), and (G) were fitted using Gaussian, exponential, or Boltzmann fitting models depending on the characteristics of each dataset and are intended solely to guide the eye and illustrate the overall temporal trend. (**H**) Proposed mechanism of UV-induced photodamage. Scale bars, 5 μm. Data are presented as mean ± SEM from twelve independent experiments. One-way ANOVA with Tukey’s post hoc test, ns: *P >* 0.05, **P < 0.01, ***P < 0.001 compared to the control.

Next, we investigated whether UV-induced ultrastructural alterations in cellular organelles were mediated by ROS. To test this hypothesis, we applied the well-known ROS scavenger, ascorbic acid, to HDF after UV exposure and subsequently examined organelle morphology using STORM imaging. As shown in Fig. 3B and fig. S14, the characteristic morphological changes observed in UV-irradiated cells, including mitochondrial fission, transition of the ER from tubular to sheet-like architecture, Golgi fragmentation and dispersal, MTOC depolymerization, and microtubule breakage, were largely absent in the ascorbic acid-treated cells. In contrast, the organelle morphology of ascorbic acid-treated HDF closely resembled that of the untreated controls, even after UV illumination. Comparable results were obtained when ascorbic acid was applied prior to UV exposure, demonstrating that ROS scavenging is effective regardless of treatment timing (fig. S14). These findings support the conclusion that UV-induced remodeling of organelle ultrastructure is a direct or indirect consequence of ROS generation.

UV-induced ROS production has been reported to cause DNA damage. To further explore whether UV-induced ROS generation leads to nuclear DNA damage under our experimental conditions, we examined the formation of γH2AX foci as a marker of DNA double-strand breaks at various time points after UV illumination. As shown in Fig. 3C, γH2AX foci appeared first, showing their maximal size at approximately 1 hour after UV exposure, which was after ROS formation. STORM imaging of HDF cells exposed to increasing UV intensities revealed a clear intensity-dependent increase in γH2AX foci within the nucleus, with both the number and size of foci increasing in proportion to the UV dose (Fig. 3C and fig. S15).

Following the detection of DNA double-strand breaks, we examined the activation dynamics of poly(ADP-ribose) polymerase-1 (PARP-1), a key DNA damage sensor rapidly recruited to DNA lesions. We utilized STORM imaging to visualize the spatiotemporal distribution of PARP-1 at various intervals following UV exposure. UV-irradiated HDFs exhibited pronounced nanoscale PARP-1 nuclear clustering, indicating localized activation and accumulation (Fig. 3D and fig. S16). Cluster frequency increased progressively, peaking at approximately 3 hours postirradiation before a gradual decline. Furthermore, PARP-1 cluster number correlated positively with UV intensity, suggesting more extensive activation and aggregation at putative DNA lesion sites (Fig. 3D). In contrast, untreated control cells displayed only sparse, diffuse PARP-1 signals, consistent with basal expression. Collectively, these results indicate that UV-induced PARP-1 activation occurs in a dose- and time-dependent manner following ROS generation and DNA damage, reflecting its central role in the DNA repair response triggered by UV-induced oxidative stress.

Following the detection of PARP-1 activation, we investigated whether UV-induced DNA damage activates downstream signaling pathways, particularly the NF-κB pathway, which is triggered by genotoxic stress. Therefore, we examined the subcellular localization dynamics of NF-κB in HDF cells at different time points after UV illumination using STORM imaging. In control (non-irradiated) cells, NF-κB signals were predominantly localized to the cytosol, consistent with their inactive state. However, upon UV exposure, a clear time-dependent translocation of NF-κB from the cytoplasm to the nucleus was observed. The relative abundance of NF-κB within the nucleus, quantified as the ratio of nuclear to cytosolic localization numbers, progressively increased after UV illumination and reached a peak approximately 6 hours postirradiation (Fig. 3E and fig. S17). This temporal pattern indicates that UV-induced oxidative stress and DNA damage trigger NF-κB activation and nuclear translocation, consistent with its established role in regulating the transcriptional response to cellular stress.

Given that NF-κB functions as a central transcription factor that regulates immune and inflammatory responses, we investigated whether its nuclear translocation upon UV irradiation was associated with the induction of pro-inflammatory cytokines. To determine which cytokines were induced through the NF-κB cascade, we performed a cytokine multiplex assay and found that UV irradiation led to significant increases in interleukin (IL)-6 and IL-8 concentrations of 241% and 560%, respectively (Fig. 3F). To visualize the spatiotemporal dynamics of cytokine expression at the single-molecule level, we performed STORM imaging of IL-6 and IL-8 in HDF at various time points after UV irradiation. As shown in Fig. 3G and figs. S18 and S19, both IL-6 and IL-8 exhibited a marked time-dependent increase in the formation of nanoscale clusters after exposure to UV light. Specifically, the number of IL-6 and IL-8 clusters increased noticeably at approximately 12 hours postirradiation and continued to increase progressively up to 24 hours postirradiation, indicating sustained cytokine upregulation in response to UV-induced stress. These observations suggest that UV illumination activates a prolonged inflammatory response, consistent with the NF-κB–mediated transcriptional induction of pro-inflammatory cytokines.

Collectively, kinetic analyses of ROS generation, PARP-1 activation, NF-κB nuclear translocation, and IL-6/IL-8 clustering revealed a well-defined temporal hierarchy of molecular events (Fig. 3H). Intracellular ROS levels exhibited the earliest peak, appearing within minutes of UV exposure, followed by the formation of γH2AX foci, which reached their maximal size approximately 1 hour post-irradiation. Subsequently, PARP-1 was rapidly recruited to DNA lesions, and NF-κB nuclear localization peaked at approximately 6 hours after UV exposure. Finally, the cytosolic cluster numbers of IL-6 and IL-8 continued to increase, reaching a maximum at approximately 24 hours. These temporal differences delineate a sequential cascade in which ROS production precedes DNA damage and PARP-1 activation, leading to NF-κB nuclear translocation and culminating in the clustering of pro-inflammatory cytokines. Taken together, these findings provide a mechanistic framework for understanding the molecular progression of UV-induced photodamage and inflammation.

### Probiotic EV-mediated protection and recovery from UV-induced photodamage in the molecular processes

Next, we investigated the mechanisms driving the protective and restorative effects of probiotic EVs at both cellular and molecular levels. To determine how *L. plantarum*-derived EVs modulate the UV-induced molecular cascade, we performed time-resolved STORM imaging of HDF treated with *L. plantarum* EVs before UV illumination. As shown in Fig. 4A, the time-course profile of intracellular ROS generation was only marginally affected by treatment with *L. plantarum* EVs, indicating that the vesicles did not completely suppress the initial oxidative events. Similarly, time-resolved STORM imaging of nuclear DNA damage markers revealed that the formation of γH2AX foci was not significantly reduced in *L. plantarum* EV-treated cells compared to UV-only controls (Fig. 4B and fig. S20).

**Fig. 4.**
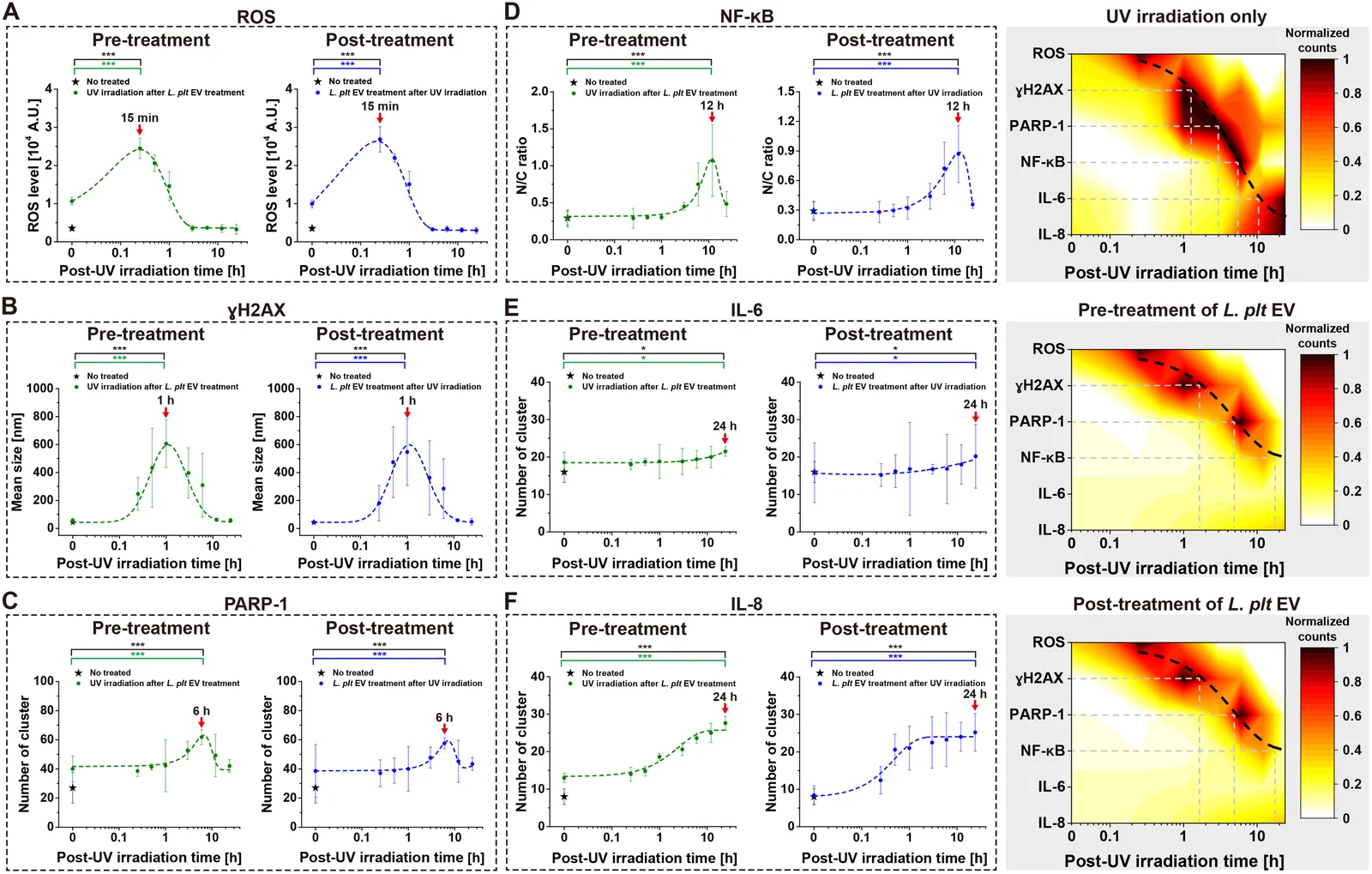
Effects of *L. plantarum* EV treatment on the mechanism of UV-induced photodamage. (**A** to **F**) Time course of intracellular ROS levels (A), γH2AX cluster size (B), PARP-1 cluster number (C), NF-κB nuclear-to-cytoplasmic (N/C) localization density ratio (D), IL-6 cluster number (E), and IL-8 cluster number (F) in HDF cells after UV irradiation with either pretreatment (left panels) or posttreatment (right panels) with *L. plantarum* EVs. At each time point, the mean values of each readout from STORM images were plotted as a function of postirradiation time to reconstruct the temporal progression of UV-induced molecular signaling. Trend lines were fitted using Gaussian or exponential fitting models depending on the characteristics of each dataset and are intended solely to guide the eye and illustrate the overall temporal trend. Red arrows: time point at which each molecular target reaches its peak value under UV irradiation with EV treatment. The heatmaps on the right side summarize the time-course data of all six molecular targets under UV irradiation only, pretreatment, and posttreatment conditions on a single shared time axis. Each target was normalized to its respective peak value to enable direct comparison of the relative timing and amplitude of activation across the signaling cascade. The dashed line in the heatmap indicates the peak position of each target. Data are presented as mean ± SEM from twelve independent experiments. One-way ANOVA with Tukey’s post hoc test, *P < 0.05, ***P < 0.001 compared to the control.

Next, we examined the time-course profile of PARP-1 cluster formation within the nucleus to determine how *L. plantarum* EVs modulate UV-induced molecular signaling. As shown in Fig. 4C and fig. S21, nanoscale PARP-1 clusters were still detected in HDF pretreated with *L. plantarum* EVs, indicating that *L. plantarum* EV treatment did not prevent PARP-1 recruitment to UV-induced DNA lesions. However, a pronounced alteration in the activation kinetics was observed despite the comparable kinetics of ROS and γH2AX. While PARP-1 cluster formation in UV-only cells reached a maximum at approximately 3 hours postirradiation, EV-treated cells exhibited a delayed peak at approximately 6 hours postirradiation. This temporal delay suggests that *L. plantarum* EVs do not directly suppress the DNA damage response but modulate the progression and amplification of downstream stress signaling initiated by PARP-1 activation. Given that excessive or rapid PARP-1 activation has been implicated in propagating inflammatory signaling cascades following genotoxic stress, the delayed PARP-1 clustering observed in EV-treated cells may reflect a buffering effect that mitigates the transmission of DNA damage signals to pro-inflammatory pathways.

A delayed downstream stress response was also observed for NF-κB in EV-treated cells. For example, STORM analysis of NF-κB demonstrated that EV treatment profoundly inhibited its nuclear translocation and clustering. Whereas UV irradiation alone induced a progressive accumulation of NF-κB within the nucleus, reaching a peak nuclear/cytosolic ratio at approximately 6 hours, cells pretreated with *L. plantarum* EVs exhibited substantially lower nuclear localization throughout all time points, with a delayed peak at approximately 12 hours (Fig. 4D and fig. S22). This result indicates that *L. plantarum* EVs directly interfere with the activation or nuclear import of NF-κB, rather than indirectly through modulation of early ROS production. Similarly, the formation of nanoscale IL-6 and IL-8 clusters was strongly suppressed in EV-treated cells. Quantitative STORM analyses showed that the number of cytokine clusters was reduced by more than 32–45% relative to the UV-only controls (Fig. 4, E and F, and figs. S23 and S24) at 24 hours postirradiation. These data indicate that EV-mediated inhibition of NF-κB activation effectively prevents the downstream transcriptional induction of pro-inflammatory cytokines.

Collectively, these observations indicate that pretreatment with *L. plantarum*-derived EVs mitigates UV-induced inflammatory responses, primarily by modulating the PARP-1–NF-κB signaling axis. Although *L. plantarum* vesicles did not fully inhibit the initial ROS generation or subsequent DNA damage, they obviously delayed PARP-1 activation and foci formation and suppressed the nuclear translocation of NF-κB. This temporal regulation leads to attenuated IL-6 and IL-8 expression and reduced cytokine clustering at the single-cell level. Together with the preservation of ultrastructural integrity in major organelles, these molecular findings suggest that *L. plantarum* EVs exert protective effects by regulating stress-responsive transcriptional pathways rather than by scavenging antioxidants alone.

To further evaluate the therapeutic potential of *L. plantarum*-derived EVs, we examined whether *L. plantarum* EV treatment after UV exposure could reverse or attenuate the ongoing photodamage cascade. Unlike pretreatment, in which *L. plantarum* EVs were applied to cells before ROS generation, posttreatment allowed us to examine whether *L. plantarum* EVs could intervene once early oxidative and genotoxic events were initiated. To investigate this, we performed time-resolved STORM imaging using the same experimental workflow as that applied to UV-photodamaged *L. plantarum* EV-pretreated cells. Time-resolved STORM imaging revealed that treatment with *L. plantarum* EVs immediately following UV illumination did not alter the initial ROS burst, as ROS had reached near-maximal levels by the time of treatment (Fig. 4A). Similarly, the size of the γH2AX clusters remained nearly unchanged compared with the UV-only controls (Fig. 4B and fig. S20), indicating that *L. plantarum* EV treatment did not markedly influence the extent of UV-induced DNA damage. These results suggest that *L. plantarum* EVs do not inhibit UV-induced DNA damage.

However, EV treatment disrupts the propagation of inflammatory signaling cascades. For example, PARP-1 nanocluster formation was delayed in a time-dependent manner following *L. plantarum* EV addition compared to UV-only cells, exhibiting a delayed peak at approximately 6 hours, suggesting that *L. plantarum* EVs may inhibit PARP-1 recruitment to damaged sites (Fig. 4C and fig. S21). Similarly, STORM imaging showed that NF-κB nuclear translocation, which progressively increased in UV-only cells, was considerably delayed and suppressed after *L. plantarum* EV application (Fig. 4D and fig. S22). The overall nuclear/cytosolic ratio remained markedly lower in the posttreatment group at peak hours. Downstream cytokine induction is also reduced. The nanoscale clustering of IL-6 and IL-8, which exhibited robust time-dependent increases in UV-only cells, was substantially reduced in *L. plantarum* EV posttreated cells (Fig. 4, E and F, and figs. S23 and S24). Quantitative analyses demonstrated that the total cluster number decreased by more than 32–45%, and the characteristic accumulation over 24 hours was almost completely attenuated. These findings indicate that posttreatment with *L. plantarum* EVs is sufficient to disrupt the continuation and amplification of UV-induced inflammatory signaling, even after its initiation.

Collectively, pre- and posttreatment analyses demonstrate that *L. plantarum* EVs effectively mitigate UV-induced photodamage. While these vesicles do not fully suppress the initial oxidative burst or γH2AX formation, they markedly attenuate and delay downstream inflammatory signaling, specifically PARP-1 activation and NF-κB nuclear translocation, which ultimately suppresses IL-6 and IL-8 clustering. The ability of *L. plantarum* EVs to modulate these molecular events, even when administered after UV exposure, highlights their therapeutic potential for reversing ongoing cellular damage. These findings, combined with the preservation of organelle ultrastructure, demonstrate that *L. plantarum* EVs function as robust modulators of inflammatory transcriptional programs rather than through passive antioxidant scavenging.

We next performed genetic and pharmacological perturbation experiments to establish the causal role of the PARP-1–NF-κB signaling axis in mediating these effects. PARP-1 depletion by siRNA suppressed UV-induced NF-κB nuclear translocation and IL-6/IL-8 clustering in a manner closely mirroring *L. plantarum* EV treatment, and additional EV treatment in PARP-1-depleted cells produced no further suppression, indicating that EV-mediated effects on downstream inflammatory signaling are dependent on PARP-1 (fig. S25). Consistent with this, pharmacological inhibition of NF-κB with TPCA-1 similarly reduced cytokine clustering, and combining TPCA-1 with EV treatment did not yield additional suppression beyond TPCA-1 alone (fig. S26). Together, these results confirm that *L. plantarum* EVs exert their anti-inflammatory effects through the PARP-1–NF-κB axis rather than through secondary or parallel pathways.

### Modulation of PARP-1 condensate-like organization by *L. plantarum* EVs

Because *L. plantarum* EVs delayed PARP-1 activation following UV exposure, we investigated their influence on the nuclear organization and assembly dynamics of PARP-1. Using STORM imaging, we characterized PARP-1 clustering kinetics at multiple intervals post-irradiation. Quantitative analysis of cluster size distributions in UV-irradiated cells revealed a progressive increase in PARP-1 clusters through 3 hours postirradiation, followed by a shift toward smaller cluster sizes (Fig. 5A). This temporal pattern reflects transient PARP-1 clustering that disperses as signaling progresses downstream. In contrast, both pre- and posttreatment with *L. plantarum* EVs markedly delayed these clustering dynamics, shifting the peak cluster size distribution to approximately 6 hours after UV exposure (Fig. 5A).

**Fig. 5.**
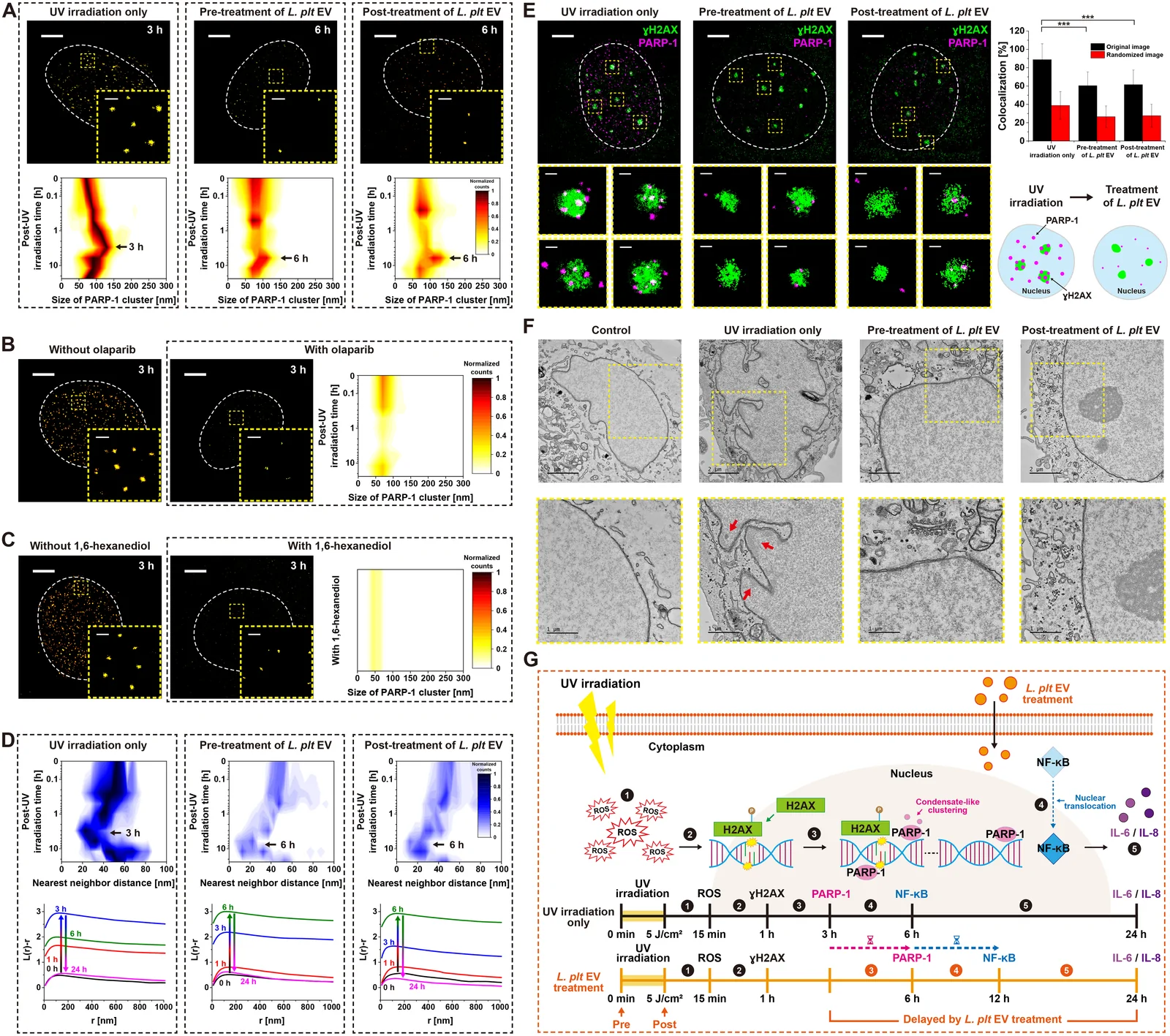
Effect of *L. plantarum*-derived EVs on condensate-like PARP-1 assembly. (**A**) Representative STORM images of PARP-1 at peak time points after UV irradiation under each condition, together with quantitative analysis of PARP-1 cluster size distributions obtained from time-resolved STORM imaging following UV exposure, with pre and posttreatment with *L. plantarum* EVs. (**B**) Representative STORM images of PARP-1 following pharmacological inhibition with the PARP inhibitor olaparib, compared with PARP-1 organization after UV irradiation in the absence of olaparib at peak time points after UV irradiation under each condition. (**C**) Representative STORM images at peak time points after UV irradiation under each condition and quantitative analysis of PARP-1 cluster size distributions after UV exposure, with and without 1,6-hexanediol treatment. (**D**) Quantitative analysis of the spatial organization of PARP-1 assemblies over time using nearest-neighbor distance measurements (top) and Ripley’s L function derived from the STORM images (bottom). (**E**) Two-color STORM imaging of γH2AX (green) and PARP-1 (magenta) following UV exposure without (left) and with (right) *L. plantarum* EV treatment. Colocalization analysis is shown on the right panel. (**F**) Transmission electron microscopy (TEM) images of nuclei from control, UV-irradiated, and UV + *L. plantarum* EV-treated cells. Red arrows indicate heterochromatin condensation. (**G**) Proposed anti-photodamage mechanism of *L. plantarum* EVs. White dashed line: nuclear boundary. Scale bars, 5 μm in [(A) to (C)] and (E), 2 μm in (F), and 500 nm in the yellow dashed box region in [(A) to (C)] and (E), and 1 μm in the yellow dashed box region in (F). Data are presented as mean ± SEM from twelve independent experiments. One-way ANOVA with Tukey’s post hoc test, ***P < 0.001 compared to UV-irradiated.

To distinguish EV-mediated modulation from direct enzymatic inhibition, we examined PARP-1 organization following treatment with the PARP inhibitor olaparib (*21*). In contrast to EV treatment, olaparib treatment resulted in a near-complete loss of detectable PARP-1 clustering in STORM images (Fig. 5B), indicating that pharmacological inhibition disrupted PARP-1 assembly. This phenotype was distinct from that observed with *L. plantarum* EV treatment, demonstrating that *L. plantarum* EVs do not suppress PARP-1 clustering in an inhibitor-like manner but regulate the temporal progression of PARP-1 assembly.

The presence of dynamic PARP-1 clusters raises the possibility that PARP-1 undergoes condensate-like organization following UV stress. To functionally examine this possibility, cells were treated with 1,6-hexanediol when PARP-1 clusters were fully established (Fig. 5C). Phase-separated condensates are typically maintained by weak multivalent interactions, and their selective sensitivity to 1,6-hexanediol is widely used as a functional criterion for distinguishing condensate-like assemblies from stable protein aggregates (*22*). Consistent with this behavior, PARP-1 clusters were rapidly and almost completely dispersed following 1,6-hexanediol treatment, rendering the clustering undetectable by STORM (Fig. 5C). These results suggest that UV-induced PARP-1 assemblies exhibit condensate-like properties driven by weak reversible interactions rather than by irreversible aggregation.

To directly validate the dynamic nature of UV-induced PARP-1 assemblies in living cells, we performed live-cell imaging using the LivePAR biosensor. Time-lapse confocal imaging revealed progressive intranuclear clustering of LivePAR signal following UV irradiation, with *L. plantarum* EV-pretreated cells exhibiting a delayed onset and reduced magnitude of clustering compared to UV-only controls (fig. S27A), consistent with the temporal dynamics observed in our time-resolved STORM data. To further characterize the biophysical properties of these assemblies, fluorescence recovery after photobleaching (FRAP) experiments on LivePAR-positive nuclear foci demonstrated rapid and near-complete fluorescence recovery following photobleaching, indicating dynamic molecular exchange with the surrounding nucleoplasm and confirming the liquid-like nature of UV-induced PARP-1 condensates (fig. S27B).

To further characterize the spatial evolution of PARP-1 assemblies over time, we analyzed the nearest-neighbor distances (NNDs) and Ripley’s L function derived from STORM localization data across the same time course (Fig. 5D). NND analysis provides a quantitative measure of the local molecular density based on the distance between each localization in a PARP-1 cluster and its closest neighbor, providing information on the onset and extent of nanoscale clustering. Following UV irradiation, the NND distributions rapidly shifted toward smaller values until 3 hours, indicating an early increase in the local PARP-1 density, consistent with the rapid nucleation of PARP-1 assemblies. As time progressed, the NND values gradually increased, reflecting the attenuation and partial dispersal of these assemblies during the later stages. In contrast, *L. plantarum* EV treatment markedly altered this temporal pattern. The reduction in NNDs occurred more slowly and reached a lower magnitude than under UV-only conditions, indicating delayed and attenuated nucleation of PARP-1 assemblies. Thus, *L. plantarum* EVs modulated the initial local interactions that drive PARP-1 clustering, delaying the formation of high-density nanoscale assemblies without preventing their clustering. To complement this local density analysis, we used Ripley’s L function to quantify the PARP-1 spatial organization across multiple length scales. Ripley’s analysis revealed that UV irradiation induced pronounced clustering over defined nanometer-scale distances, as reflected by an increased deviation of L(r) from complete spatial randomness and a prominent peak in L(r)−r at early time points until 3 hours after UV irradiation. As shown in Fig. 5D, the position and amplitude of this peak evolved over time, which was consistent with the maturation and coalescence of PARP-1 assemblies. In *L. plantarum* EV-treated cells, the increase in clustering was delayed, indicating that *L. plantarum* EVs alter the timing and spatial progression of PARP-1 assembly across nanoscale lengths. Together, the combined NND and Ripley’s L function analyses demonstrated that *L. plantarum* EVs reshaped the nucleation–maturation trajectory of PARP-1 assemblies by delaying the development of a higher-order spatial organization. These findings support a model in which *L. plantarum* EVs kinetically tune condensate-like PARP-1 assembly rather than inhibiting it, modulating the physical basis of downstream DNA damage signaling.

Next, we investigated whether *L. plantarum* EV-mediated modulation of PARP-1 assembly kinetics affected its spatial coupling to DNA damage sites. Two-color STORM imaging of γH2AX and PARP-1 revealed significant colocalization of PARP-1 with γH2AX-marked DNA damage foci following UV irradiation, indicating that PARP-1 is rapidly activated and recruited to DNA damage sites under UV stress (Fig. 5E). To test whether this colocalization was statistically meaningful rather than arising from random spatial overlap, we performed a randomization control in which γH2AX cluster locations were spatially randomized within the nuclear region while preserving the original localization density. Colocalization analysis revealed that the percentage of γH2AX-marked DNA damage foci overlapping with PARP-1 localization in the original images was significantly higher than that obtained from the randomized datasets, confirming that PARP-1 is non-randomly and preferentially enriched at DNA damage sites following UV irradiation. We next performed two-color STORM imaging of γH2AX and PARP-1 in UV-irradiated cells treated with *L. plantarum* EVs and conducted quantitative colocalization analysis to determine how *L. plantarum* EV pre or posttreatment altered the spatial association between PARP-1 assemblies and DNA damage sites. *L. plantarum* EV treatment significantly reduced this colocalization to 60–62%, despite comparable γH2AX formation, indicating that *L. plantarum* EVs attenuate the recruitment and sustained organization of PARP-1 at damage sites.

Finally, we investigated whether the modulation of PARP-1 assembly kinetics was reflected in the ultrastructural changes within the nucleus. TEM analysis of UV-irradiated cells revealed pronounced nuclear abnormalities, including nuclear envelope invaginations, irregular nuclear morphology, and aberrant heterochromatin condensation, indicating a stress-associated loss of nuclear structural integrity and a stress-associated pathological chromatin state (Fig. 5F). In contrast, cells treated with *L. plantarum* EVs following UV exposure exhibited preserved nuclear mechanical integrity, reduced heterochromatin overcondensation, preserved euchromatin regions, smoother chromatin contrast, and a more homogeneous nucleoplasmic texture. Although condensate-like assemblies, such as PARP-1 clusters, are not expected to be directly visualized by conventional TEM because of their dynamic, hydration-rich, and membrane-less nature, these ultrastructural differences follow the downstream consequences of altered PARP-1 organizational kinetics. While UV stress alone resulted in a heterogeneous, grainy nucleoplasmic appearance and perinuclear chromatin collapse, EV treatment preserved nuclear integrity, yielding a smoother nuclear envelope and reduced chromatin disorganization.

To further examine whether NAD^+^, identified as a key functional constituent of *L. plantarum* EVs, contributes to the observed delay in PARP-1 clustering, we performed time-resolved STORM imaging of PARP-1 under NAD^+^ pre and posttreatment conditions. NAD^+^-loaded liposomes applied to UV-photodamaged HDF cells similarly delayed the peak of PARP-1 clustering from ∼3 hours to ∼6 hours after UV exposure (fig. S9), closely mirroring the temporal shift observed with *L. plantarum* EV treatment. These results suggest that the EV-mediated delay in PARP-1 condensate assembly is at least in part attributable to NAD^+^ delivered by the vesicles.

Collectively, these results demonstrate that *L. plantarum* EVs did not suppress PARP-1 activity through direct inhibition but delayed and attenuated the condensate-like assembly of PARP-1 by modulating its spatial and temporal organization in the nucleus (Fig. 5G). This kinetic tuning limits the excessive recruitment of PARP-1 to DNA damage sites and suppresses the amplification of downstream inflammatory signaling, including NF-κB activation, while preserving early DNA damage sensing. Thus, our findings reveal a previously unrecognized mechanism by which probiotic EVs modulate stress-responsive nuclear organization to mitigate UV-induced inflammatory aging.

## DISCUSSION

In this study, we established a nanoscale mechanistic framework for UV-induced photodamage and its modulation by *L. plantarum* EVs using STORM. By directly visualizing molecular clustering and organelle ultrastructure at the single-cell level, our study revealed that probiotic EVs do not suppress photodamage but reprogram the temporal organization of stress-responsive nuclear signaling.

UV irradiation induces pronounced ultrastructural organelle damage and rapid DNA damage signaling. Super-resolution imaging revealed that PARP-1 undergoes transient, time-dependent clustering following UV exposure, suggesting that its activation is regulated by assembly dynamics rather than a binary on/off switch. Notably, *L. plantarum* EVs significantly delayed PARP-1 clustering without preventing the initial DNA damage, suggesting that these EVs modulate the kinetics of the nuclear stress response rather than acting as direct inhibitors. This kinetic delay is associated with reduced nuclear translocation and nanoscale clustering of NF-κB, subsequently attenuating IL-6 and IL-8 expression. Combined with the preservation of organelle ultrastructure, these findings support a hierarchical model in which *L. plantarum* EVs maintain cellular integrity by inhibiting the propagation of dysregulated nuclear stress signaling, positioning temporal control as a key determinant of cellular resilience to UV-induced damage. This activity may correspond to a calming effect on UV-induced dermal inflammatory and stress responses in human dermal fibroblasts and a reconstructed dermal tissue model.

We propose a conceptual framework where UV-triggered PARP-1–mediated nuclear clustering serves as a signaling hub that activates inflammatory programs and organelle remodeling. *L. plantarum* EVs intervene early in this cascade by buffering PARP-1 assembly dynamics, effectively uncoupling DNA damage sensing from excessive inflammatory amplification. Our data suggest that these EVs do not inhibit PARP-1 condensation per se; instead, they tune the multivalent interactions underlying condensate nucleation and maturation. By modulating the interaction landscape, likely through PARP-1-dependent multivalency and chromatin-associated anchoring, probiotic EV treatment yields kinetically tuned, less inflammatory PARP-1 assemblies, ultimately mitigating photodamage-associated dysfunction. Super-resolution imaging is essential for elucidating this mechanism. Conventional ensemble assays would capture only the average changes in protein levels or localization, whereas STORM imaging reveals nanoscale clustering behaviors and time-dependent effects that define the regulatory role of *L. plantarum* EVs. Although bacterial EVs, including those from probiotic strains such as *L. plantarum*, have been shown to modulate host inflammatory responses and cell signaling pathways through the delivery of bioactive cargo, no reports have demonstrated the direct regulation of biomolecular condensates or phase separation processes by probiotic EVs. Therefore, our results underscore the value of super-resolution microscopy not only as an imaging tool but also as a mechanistic approach for dissecting stress response pathways in complex cellular environments.

In summary, we demonstrate that *L. plantarum* EVs exert anti-photodamage effects by modulating the spatiotemporal organization of nuclear stress signaling, rather than via the direct suppression of DNA damage or oxidative stress. By delaying PARP-1 clustering and downstream NF-κB activation, these findings establish a new conceptual framework for probiotic EV-mediated photoprotection, highlighting the critical role of temporal control in regulating inflammatory and aging-associated pathways. Notably, the UV-induced organelle remodeling and inflammatory responses described here mirror cellular phenotypes associated with several hallmarks of aging (*23*), including mitochondrial dysfunction and chronic inflammation. Also, the ability of LDNPs to recapitulate the EV-mediated preservation of organelle ultrastructure in UV-stressed dermal fibroblasts indicates that LDNP formulations can reproduce the core functional effects of native EVs characterized in this study. Importantly, the restoration of collagen I following EV treatment further extends these findings beyond intracellular structural recovery, demonstrating preservation of a major component of the dermal extracellular matrix. Together, these results provide mechanistic insight into how probiotic microbiome-derived EVs modulate cellular responses to UV-induced photodamage and preserve cellular homeostasis. Although emerging evidence for microbiome–host communication and the gut–skin axis provides broader biological context for the potential effects of probiotic-derived EVs, whether and how these vesicles reach skin tissues in vivo remains to be established (*24*). Similarly, direct topical delivery of vesicles of this size across the stratum corneum remains challenging, and the development of effective delivery strategies represents a separate area for future investigation (*25*). Thus, further studies using chronic UV exposure and in vivo models will be important for determining whether the cellular mechanisms identified here can ultimately be translated into strategies for preventing or mitigating skin photodamage.

## MATERIALS AND METHODS

### Purification of *L. plantarum* EVs

EVs from cultured *L. plantarum* OD11 (KCTC16121BP) were obtained through sequential ultracentrifugation, first at 10,000*g* for 30 min and subsequently at 150,000*g* for 2.5 hours (Hitachi, Chiyoda-ku, Tokyo, Japan). The pellets collected from this process were dissolved in distilled water to a final volume of 100 ml and passed through a 0.22 μm bottle-top filter. The average size and vesicle concentration of the purified EVs were measured as 207.8 nm and 2.5 × 10^9^ particles/ml, respectively, using nanoparticle tracking analysis (NTA; Zetaview, Particle Metrix, Germany).

To generate LDNPs, *L. plantarum* OD11 (KCTC16121BP), originally isolated from Korean mulberry (*Morus alba*), was grown in MRS broth at 30°C with shaking at 180 rpm for 18 hours. Bacterial cells were harvested by centrifugation (1,500 rpm, 5 min) and resuspended in 500 ml of PBS. The suspension was then processed by high-pressure homogenization using a microfluidizer (MN400BF, Micronox, Korea) at 2,000 bar for five consecutive passes. The resulting nanoparticle suspension was supplemented with preservatives (5% butylene glycol and 3% hexanediol) and subsequently passed through a 0.22 μm bottle-top filter for sterilization.

LDNPs were introduced in this study to verify whether the bioactive effects of naturally secreted *L. plantarum* EVs are attributable to their specific cargo and lipid bilayer architecture rather than to the natural secretion process itself. As previously reported, LDNPs closely mimic the physical and biological characteristics of native EVs, including size distribution, membrane structure, and cellular uptake efficiency, and are therefore well suited as a scalable and reproducible alternative to naturally secreted EVs for functional validation purposes.

### Sample preparation for STORM imaging

HDF cells (ATCC, CRL-1635) were cultured in Dulbecco’s Modified Eagle’s medium (DMEM; LM 001–05, Welgene) supplemented with 10% (v/v) fetal bovine serum (FBS; A31605, Thermo Fisher Scientific) and 1% (v/v) penicillin-streptomycin (15070; Thermo Fisher Scientific). Cells were maintained at 37°C in a humidified incubator with 5% CO_2_ (*17*).

For STORM imaging, HDF cells were seeded onto glass-bottom confocal dishes (100350; SPL Life Science) and cultured under standard conditions (37°C, 5% CO_2_, humidified atmosphere) for 1–2 days. After incubation, the cells were gently washed with pre-warmed Dulbecco’s phosphate-buffered saline (DPBS; DPB-001, SolBio), which was replaced with phenol red-free DMEM (A1896701; Thermo Fisher Scientific). To examine cellular responses to different UV doses, cells were exposed to UV irradiation (365 nm) at 3, 5, or 7 J/cm^2^ under dark conditions (*18*, *19*) and then cultured in fresh medium for 24 hours before imaging. To monitor the sequence and extent of molecular expression following UV exposure, the cells were first irradiated with 5 J/cm^2^ UV light and then incubated without UV illumination for varying durations. Finally, to investigate the therapeutic effects of *L. plantarum* EVs and LDNPs, the cells were treated with 6.75 × 10^7^ particles/mL of *L. plantarum* EVs and LDNPs after UV exposure (*20*). To further examine the functional contribution of NAD^+^, cells were additionally treated with NAD^+^-containing liposomes, provided by the R&D center of LG H&H. The dose of NAD^+^-containing liposomes was adjusted to provide an NAD^+^ concentration equivalent to that present in 6.75 × 10^7^ particles/mL of *L. plantarum* EVs, calculated based on quantitative results reported in a previous study (*20*). As a control, empty liposomes (NAD^+^-free) were prepared and applied at the same lipid concentration as the NAD^+^-containing liposomes. Similarly, the dose of *S. epidermidis* EVs was determined based on the quantitative NAD^+^ analysis reported in the previous study to provide an equivalent amount of NAD^+^, serving as the unrelated bacterial EV control. For the pretreatment groups, cells were treated with *L. plantarum* EVs, LDNPs, *S. epidermidis* EVs, NAD^+^-containing liposomes, or NAD^+^-free liposomes before UV exposure, followed by the same UV irradiation and incubation procedures described above. After UV irradiation and EV treatment, the cells were gently washed with DPBS and fixed at room temperature (RT) for 10 min in a solution containing 3% (v/v) paraformaldehyde (PFA; 15714, Electron Microscopy Sciences) and 0.1% (v/v) glutaraldehyde (GA; 16020, Electron Microscopy Sciences). Following fixation, the cells were washed with DPBS, and residual aldehydes were quenched with 0.1% (v/v) sodium borohydride (16940–66-2; Sigma-Aldrich) for 7 min at RT. For permeabilization, the cells were treated with 0.25% (v/v) Triton X-100 (22140; Electron Microscopy Sciences) for 10 min and washed with DPBS. To minimize nonspecific antibody binding, the cells were blocked with 3% (w/v) bovine serum albumin (BSA; CNB102–0100, CellNest) for 30 min at RT. Primary antibodies diluted in 3% BSA were added to the cells and incubated for 40 min at RT. After washing with DPBS, the cells were incubated with the corresponding secondary antibodies diluted in 3% BSA for 1 hour at RT in the dark. To further fix the antibody labels, the cells were postfixed with 2% (v/v) PFA and 0.05% (v/v) GA for 10 min at RT. Finally, the imaging buffer was added immediately before STORM imaging after washing. If imaging was not performed immediately, samples were stored at 4°C in a NaN_3_-containing buffer to inhibit bacterial infection. All immunolabeling procedures were performed according to previously described and well-established protocols (*26*–*29*).

The primary antibodies used in this study were as follows: rabbit polyclonal anti-TOM22 (ab246862; Abcam) targeting the outer mitochondrial membrane; rabbit monoclonal anti-KLC3 (ab180523; Abcam) for ER labeling; mouse monoclonal anti-GM130 (610822; BD Transduction Laboratories) for Golgi apparatus detection; rabbit monoclonal anti-IL-6 (30399S; Cell Signaling Technology) specific to IL-6; rabbit monoclonal anti-IL-8 (ab289967; Abcam) for IL-8 labeling; rabbit monoclonal anti-NF-κB p65 (8242S; Cell Signaling Technology) for tracking the intracellular localization of NF-κB; rabbit monoclonal anti-PARP-1 (ab191217; Abcam) for assessing PARP-1 activity; rat monoclonal anti-tubulin (ab6160; Abcam) for microtubule visualization; mouse monoclonal anti-acetyl alpha tubulin (32–2700; Thermo Fisher Scientific) for MTOC; mouse monoclonal anti-gamma H2A.X (phospho S139) (ab26350; Abcam) for detecting DNA damage; and rabbit monoclonal anti-collagen I antibody (ab138492; Abcam) for visualizing intracellular collagen and extracellular collagen fibrils. The secondary antibodies used were Alexa Fluor 647 (AF647)-conjugated donkey anti-mouse IgG (A-31571; Thermo Fisher Scientific), AF647-conjugated donkey anti-rabbit IgG (A-31573; Thermo Fisher Scientific), AF647-conjugated goat anti-rat IgG (A-21247; Thermo Fisher Scientific), and AF488-conjugated donkey anti-mouse IgG (A-17832; Thermo Fisher Scientific).

For nuclear staining, Hoechst 33342 solution (62249; Thermo Fisher Scientific) was diluted in DPBS and applied to the cells under light-protected conditions immediately before the STORM imaging.

### Sample preparation for microtubule depolymerization

To assess the contribution of microtubule depolymerization to UV-induced organelle disruption, cells were pretreated with nocodazole (M1404; Sigma-Aldrich) (*30*), a microtubule-depolymerizing agent, at 700 nM at 37°C for 30 min. After treatment, the cells were washed with DPBS and the medium was replaced with fresh DMEM. The cells were then incubated for 24 hours, fixed, and subsequently subjected to immunolabeling as described above.

### Cell viability test

Cell viability was determined using a trypan blue solution (T8154; Sigma-Aldrich). HDF cells were cultured to approximately 70% confluency and washed briefly with DPBS after removal of the culture medium. Cells were detached using trypsin (12605–010; Thermo Fisher Scientific) for 5 min at 37°C under 5% CO_2_ and subsequently collected in fresh DMEM. The cell suspension was centrifuged at 200*g* for 5 min, and the pellet was resuspended in DMEM to form a homogeneous single-cell suspension. For viability measurement, an equal volume of trypan blue solution was added to the cell suspension. Then, 10 μl of the mixture was loaded onto a hemocytometer (0650030; MARIENFELD), and cells were examined under a light microscope. Live and dead cells were counted in the four corner chambers of the hemocytometer, and the percentage of viable cells was quantified using the Cell Counter plugin in ImageJ software.

### Sample preparation for ROS detection

For ROS detection, the cultured cells were washed once with pre-warmed DPBS and incubated with 10 μM DCFH-DA (D6883; Sigma-Aldrich) diluted in DPBS for 20 min at 37°C. After incubation, the cells were washed with DPBS and fixed with 4% (v/v) PFA for 10 min at RT (*31*, *32*). Fluorescence imaging was performed to visualize the generation of ROS.

Ascorbic acid (PHR1008; Sigma-Aldrich) was used as an ROS scavenger to remove ROS from the cells. Cells were exposed to UV irradiation at a dose of 5 J/cm^2^ and incubated for 24 hours in a culture medium containing 100 μM ascorbic acid (*33*). The cells were treated with ascorbic acid before or after UV exposure. After treatment, the cells were fixed and processed for sample preparation as described above.

### Sample preparation for PARP-1 perturbation

To investigate whether the PARP-1 clusters were condensate-like organizations, cells were incubated until sufficient PARP-1 clusters were formed after UV irradiation, followed by treatment with 1,6-hexanediol (240227; Sigma-Aldrich). A 1.5% (v/v) 1,6-hexanediol solution was freshly prepared in DMEM and pre-warmed immediately before use (*34*). The 1,6-hexanediol–containing medium was then added to the cell-seeded dishes, and the cells were incubated at 37°C for 2 min (*34*). Immediately after treatment, the cells were fixed and immunolabeled for STORM microscopy.

We also tested the PARP inhibitor, olaparib (SML3705; Sigma-Aldrich), for comparison. The cells were pretreated with 5 μM olaparib at 37°C for 1 hour before UV irradiation, and then washed to remove residual olaparib (*35*). After the cells were subjected to UV irradiation under the indicated conditions, the culture medium was replaced with fresh DMEM, and the cells were further incubated for various time points. At each time point, the cells were fixed and processed for immunolabeling, as previously described.

### siRNA-mediated knockdown of PARP-1 expression

Prior to PARP-1 knockdown, HDF cells were cultured in antibiotic-free DMEM for 24 hours at 37°C. To knock down PARP-1 expression, three Stealth RNAi siRNA duplexes targeting PARP-1 (HSS100243, HSS100244, and HSS100245; Thermo Fisher Scientific) were designed using the Stealth Designer algorithm. A non-targeting Stealth RNAi siRNA was used as a negative control (12935300; Thermo Fisher Scientific) (*36*). The sequences of the siRNA duplexes were as follows: 5′-ACU CCU ACU ACA AGC UGC AGC UUC U-3′, 5′-AGA AGC UGC AGC UUG UAG UAG GAG U-3′ (HSS100243); 5′-GCG UAU GAC UUG GAA GUC AUC GAU A-3′, 5′-UAU CGA UGA CUU CCA AGU CAU ACG C-3′ (HSS100244); 5′-GGA GAC CCA AUA GGC UUA AUC CUG U-3′, 5′-ACA GGA UUA AGC CUA UUG GGU CUC C-3′ (HSS100245).

Each lyophilized siRNA duplex was reconstituted in RNase-free water to a stock concentration of 20 μM. HDF cells were incubated in culture medium without antibiotics for 24 hours prior to transfection at approximately 70% confluency. To enhance knockdown efficiency, the three PARP-1-targeting siRNAs were pooled and transfected into HDF cells in Opti-MEM (11058021; Thermo Fisher Scientific) using Lipofectamine RNAiMAX reagent (13778030; Thermo Fisher Scientific) according to the manufacturer’s instructions. The total final concentration of the pooled siRNAs was adjusted to 50 nM for STORM imaging and 10 nM for Western blot analysis. Following transfection, cells were incubated for 48 hours to allow PARP-1 knockdown. Cells were subsequently exposed to UV irradiation and treated with *L. plantarum* EVs. Following treatment, Western blot analysis was performed to validate PARP-1 knockdown efficiency, and STORM imaging was used to observe the effects of PARP-1 depletion on UV-induced cellular response.

### Western blot

Western blot analysis was performed based on a previously reported protocol (*37*, *38*). Briefly, PARP-1-knockdown HDF cells were washed with pre-warmed DPBS and lysed on ice for 30 min using RIPA buffer (89901; Thermo Fisher Scientific) supplemented with a protease inhibitor cocktail (P8340; Sigma-Aldrich). The cell lysates were then centrifuged at 12,000–14,000 rpm for 20 min at 4°C, and the supernatants were collected for further analysis.

Protein concentrations were determined using a BCA protein assay kit (23225; Thermo Fisher Scientific) according to the manufacturer’s instructions. A total of 20 μg of proteins from each sample were mixed with Laemmli sample buffer (S3401; Sigma-Aldrich) and heated for 10 min prior to electrophoresis. Proteins were separated on 4–20% polyacrylamide gel electrophoresis (4561093; Bio-Rad) and subsequently transferred onto PVDF membranes using a Trans-Blot Turbo (BR17001917; Bio-Rad). For immunoblotting, membranes were blocked with 5% non-fat dry milk (M7409; Sigma-Aldrich) in Tris-buffered saline with Tween 20 (TBST) for 1 hour at RT and then incubated with primary antibodies overnight at 4°C. Following three washes with TBST, membranes were incubated with secondary antibody for 2 hours. After additional washing steps, protein bands were visualized using ECL substrate (34577; Thermo Fisher Scientific). Chemiluminescence signals were detected and recorded using a Gel documentation system (iBright FL1500; Thermo Fisher Scientific), and band intensities were quantified using ImageJ.

The following antibodies were used for immunoblotting: rabbit monoclonal anti-PARP-1 antibody (ab191217; Abcam), rabbit monoclonal anti-collagen I antibody (ab138492; Abcam), anti-β-actin antibody (ab8227; Abcam), and HRP-conjugated donkey anti-rabbit IgG (H + L) cross-adsorbed secondary antibody (SA1–200; Invitrogen).

Protein signals were quantified using iBright Analysis Software (Thermo Fisher Scientific). Relative PARP-1 and collagen I expression was calculated by normalizing the local background-corrected volume of each target protein band to the corresponding local background-corrected volume of the β-actin band.

### Sample preparation for NF-κB inhibition

To inhibit NF-κB signaling, cells were pretreated with 20 μM TPCA-1 (2559; Tocris Bioscience), a selective inhibitor of IKK2, for 30 min at 37°C (*39*). Cells were then exposed to UV irradiation at a dose of 5 J/cm^2^ and incubated in fresh DMEM. The incubation period varied depending on the organelle or molecule analyzed and was selected based on the time point at which the maximal response was observed. Following incubation, cells were fixed, subjected to immunolabeling, and imaged using STORM.

### Sample preparation for NAD^+^ depletion

Intracellular NAD^+^ levels in HDF cells were depleted by treatment with 100 nM FK866 (F8557; Sigma-Aldrich) (*40*), an inhibitor of NAD^+^ biosynthesis, for 24 hours at 37°C. Following FK866 treatment, the cells were rinsed with DPBS to remove the inhibitor and subsequently exposed to UV irradiation. After UV exposure, *L. plantarum* EVs were added, and the cells were maintained in fresh DMEM for the designated period according to each experimental condition. Thereafter, samples were prepared for STORM imaging through fixation, permeabilization, blocking, labeling, and postfixation, following the procedure described above.

### Immunology multiplex assay

To measure cytokine expression levels in the cell culture supernatant, the cells were cultured in fresh medium to facilitate interactions between the cells and the substances for 24 hours at 37°C in a humidified incubator with 5% CO_2_. After incubation, the cells were gently washed with DPBS and detached using trypsin for 5 min. The detached cells were collected by adding DMEM and centrifuging at 200*g* for 5 min to pellet the cells. The resulting culture supernatant was collected and analyzed for cytokine content using a multiplex assay kit (Merck; HCYTA-60 K-08) on a Luminex system (Luminex 200 machine and xPONENT software). The panel included pro-inflammatory cytokines (IL-2, IL-6, IL-8, and IL-12(p70)) and the anti-inflammatory cytokine IL-10. All measurements were performed in duplicate.

### NAD^+^ quantification

To determine intracellular NAD^+^ levels, cells subjected to each treatment condition were detached by trypsinization and collected by centrifugation at 200*g* for 5 min. The collected cell pellets were resuspended in extraction buffer and lysed through two consecutive freeze–thaw cycles. Following vortex mixing, the lysates were centrifuged at maximum speed for 5 min, and the resulting supernatants were transferred to new tubes. Then, the supernatants were passed through 10 kDa spin columns (ab93349; Abcam) by centrifugation at 10,000*g* for 10 min.

Intracellular NAD^+^ levels were subsequently analyzed using the NAD/NADH Assay Kit (ab65348; Abcam) according to the manufacturer’s protocol and as previously described (*20*). Total NAD (NAD^+^ + NADH) and NADH concentrations were measured separately, and the amount of NAD^+^ was determined by subtracting the NADH value from the total NAD value. For selective NADH measurement, sample aliquots were incubated at 60°C for 30 min to decompose NAD^+^. An NADH standard curve was prepared using the supplied NADH standard solution. Background control wells received the background reaction mixture, whereas experimental wells containing total NAD or NADH samples were incubated with the master reaction mixture at RT for 5 min. After addition of the developer solution, absorbance changes were recorded for 1–4 hours. Optical density was measured at 450 nm using a microplate reader (SpectraMAX PLUS 384; Molecular Devices). Final NAD^+^ concentrations were obtained by subtracting background absorbance from each sample value, followed by interpolation using the NADH standard curve and correction for dilution factors.

### Sample preparation for EV labeling and cellular uptake

To label *L. plantarum* EVs, EVs were stained using the CellTrace CFSE Cell Proliferation Kit (C34570; Thermo Fisher Scientific). A 10 mM stock solution of the dye in DMSO was diluted in DPBS to a final working concentration of 40 μM, and 60 μl of this working solution was prepared. *L. plantarum* EVs (2.5 × 10^9^ particles/ml) were mixed with the 60 μl dye working solution and incubated at 37°C for 2 hours, with gentle agitation by tapping the tube every hour to ensure homogeneous labeling (*41*, *42*).

After incubation, unbound dye was removed and dye-labeled EVs were isolated by size-exclusion chromatography using a NAP-5 column (17085302; Cytiva). DPBS was used as the elution buffer, and a total of eight fractions of 120 μL each were collected, of which fraction 3 was used for subsequent experiments. All procedures were performed under light-protected conditions to prevent dye quenching.

To track the intracellular uptake of EVs, seeded HDF cells were washed, and pre-warmed DMEM containing dye-labeled EVs was added, followed by incubation at 37°C for 2 hours. As a dye-only control, CellTrace Far Red dye was processed through the same purification procedure in the absence of EVs, and the resulting fraction was added to HDF cells. After incubation, the cells were washed with DPBS, fixed with 4% (v/v) PFA for 10 min, and the nuclei were stained with Hoechst 33342 solution for 1 min. Fluorescence uptake of dye-labeled EVs was subsequently examined using a confocal microscope (Stellaris 5; Leica Microsystems).

### Sample preparation of human skin tissue imaging

To determine whether the protective effects observed in HDF cells were similarly reproduced at the tissue level, a reconstructed human skin tissue model, Neoderm-ED (Tego Science; Seoul, Korea), was exposed to UV irradiation and subsequently treated with 6.75 × 10^7^ particles/ml of *L. plantarum* EVs. Following treatment, the tissues were maintained in antibiotic-free maintenance medium and incubated for 24 hours at 37°C in a humidified atmosphere containing 5% CO_2_.

The tissues were then fixed with 4% PFA for 15 min and sectioned into 100 μm-thick slices. For permeabilization, tissue sections were incubated with 0.1% (v/v) Triton X-100 for 15 min, followed by washing with DPBS. The sections were subsequently blocked with 10% (w/v) BSA for 2 hours at RT. Primary antibodies were diluted in the same blocking solution and incubated with the tissue sections for 24 hours. After three washes with DPBS, the sections were incubated with the appropriate secondary antibodies for 24 hours. Finally, nuclei were stained with Hoechst 33342. The stained tissue sections were mounted using VECTASHIELD Antifade Mounting Medium (H-1000; Vector Laboratories) and subsequently imaged using a confocal laser scanning microscope (Stellaris 5; Leica Microsystems).

### LivePAR-GFP transfection and live-cell imaging

Plasmid transfection was performed following a previously described approach (*43*). HDF cells were seeded in glass-bottom confocal dishes one day before transfection to reach approximately 70–90% confluency at the time of transfection. Cells were transiently transfected with pLV-EF1A-LivePAR-Hygro (176063; Addgene) or pLV-EF1A-LivePAR(R163A)-Hygro (187609; Addgene) plasmids using Lipofectamine LTX reagent with PLUS reagent (A12621; Thermo Fisher Scientific). For each confocal dish, 2.5 μg of plasmid DNA was diluted in 125 μL of Opti-MEM (11058021; Thermo Fisher Scientific) and mixed with 2.5 μ of PLUS reagent. Separately, 12.5 μ of Lipofectamine LTX reagent was diluted in 125 μ of Opti-MEM. The diluted DNA/PLUS mixture was combined with the diluted Lipofectamine LTX mixture and incubated for 5 min at RT. The resulting 250 μ transfection complex was added dropwise to cells maintained in 2 m of complete DMEM. Cells were gently rocked to distribute the complex evenly and incubated at 37°C in a humidified incubator with 5% CO_2_. GFP expression and cell morphology were assessed 24–48 hours after transfection, and cells with moderate GFP expression with healthy morphology were used for subsequent live-cell imaging.

For live-cell imaging, transfected HDF cells were transferred to phenol red-free culture medium. Nuclei were labeled with Hoechst 33342 under light-protected conditions, followed by washing with fresh medium. For EV treatment groups, *L. plantarum* EVs were applied for 30 min before UV irradiation. Baseline GFP and nuclear images were acquired before UV irradiation. Cells were then exposed to 5 J/cm^2^ UV irradiation, and time-lapse fluorescence images were collected immediately after UV exposure using a live-cell imaging chamber maintained at 37°C and 5% CO_2_.

### Confocal imaging

For confocal imaging, the fixed samples were maintained in DPBS during image acquisition. Images were acquired using a confocal laser scanning microscope (Stellaris 5; Leica Microsystems) equipped with a 63× oil-immersion objective lens (HC PL APO CS2 63×/1.40 Oil; Leica Microsystems). AF647-labeled structures were excited with a 638 nm laser, and fluorescence emission was collected between 650 and 750 nm using a hybrid detector (HyD). Hoechst 33342-stained nuclei were excited with a 405 nm laser, and fluorescence emission was collected between 420 and 600 nm using a HyD detector. In addition to fluorescence imaging, phase-contrast images were acquired to visualize cell membrane.

Image acquisition was performed in line-scanning mode with a line average of 4, a scan speed of 600 Hz, and a pinhole diameter of 1 Airy unit. For three-dimensional visualization of dye-labeled EV uptake, z-stack images were collected at 1 μm intervals over a total of 10 optical sections. Images were obtained at a resolution of 1024 × 1024 pixels, corresponding to a pixel size of approximately 180.4 nm. Maximum-intensity projection images were subsequently generated from the acquired z-stack datasets for image analysis and presentation.

For imaging of the reconstructed human skin tissue, tissue sections were mounted using VECTASHIELD Antifade Mounting Medium prior to image acquisition. Z-stack images were acquired over a depth range of 70–100 μm and divided into 10 optical sections. Line averaging was performed three times for each optical section, and all images were obtained at a resolution of 1024 × 1024 pixels, corresponding to a pixel size of approximately 180.4 nm. Maximum-intensity projection images were subsequently generated from the acquired z-stack datasets for image analysis and presentation.

For live-cell imaging and FRAP experiments, HDF cells expressing LivePAR-GFP were cultured in glass-bottom confocal dishes and maintained in phenol red-free DMEM during image acquisition (*44*). LivePAR-GFP fluorescence was excited using a 488 nm laser, and fluorescence emission was collected between 500 and 650 nm using a HyD detector. Hoechst 33342-stained nuclei were excited using a 405 nm laser, and fluorescence emission was collected between 420 and 490 nm using a HyD detector. Live-cell images were acquired at a resolution of 1024 × 1024 pixels, corresponding to a pixel size of approximately 51.54 nm, whereas FRAP images were acquired at a resolution of 256 × 256 pixels, corresponding to a pixel size of approximately 206.75 nm. For FRAP measurements, pre-bleach images were acquired every 7 s for 35 s. Photobleaching was subsequently performed by illuminating a region of interest (ROI) containing the LivePAR-GFP cluster signal with a 488 nm laser at 100% power for 10 s. Following photobleaching, post-bleach images were acquired every 7 s for 210 s to monitor fluorescence recovery.

### STORM imaging

For STORM imaging, fluorophore-labeled cell samples were imaged in an imaging buffer composed of 100 mM 2-mercaptoethylamine (30070; Sigma-Aldrich), 5% (w/v) glucose (50–99-7; Sigma-Aldrich), and an oxygen-scavenging system containing 0.5 mg/mL glucose oxidase (G2133; Sigma-Aldrich) and 38 μg/ml catalase (C3515; Sigma-Aldrich), all dissolved in DPBS (pH 8.5) (*45*).

STORM imaging was performed using a custom-built system (Center for Polymers and Composite Materials) based on an inverted microscope (Ti2-U; Nikon) equipped with a 100× oil-immersion objective lens (NA 1.49; CFI SR HP Apo TIRF; Nikon) (*46*). A 647 nm laser was used for excitation, and a weak 405 nm laser (0.1–1 mW) was used to reactivate the fluorophores. A 561 nm laser was used for EV imaging, whereas a 488 nm laser operated at 2 mW was used for ROS measurements. To acquire 3D information on the cellular organelles, a cylindrical lens with a focal length of 500 mm (LJ1144RM-A; Thorlabs) was inserted into the detection path to induce astigmatism, which caused the point spread function (PSF) to elongate along the x- or y-axis depending on the axial (z) position of the fluorophore. In this 3D imaging setting, molecules located above or below the focal plane exhibited distinct elliptical shapes. Based on these PSF variations, the x, y, and z coordinates of individual molecules were determined to reconstruct 3D super-resolution images.

A CRISP autofocus system (ASI) was used to maintain focal stability during long-term image acquisition using the reflection of an infrared beam at the sample–liquid interface. Fluorescence signals were filtered using bandpass filters (LF408/488/561/635-B; Semrock) and detected using an electron-multiplying charge-coupled device (EMCCD) camera (iXon Ultra 888; Andor) (*47*). The STORM datasets of cellular organelles were collected with 50,000 frames at 70 Hz, whereas those of IL-6, IL-8, γH2AX, and NF-κB comprised 40,000 frames. For imaging of HDF cells treated with CFSE-labeled EVs, 20,000 frames were acquired at 50 Hz. For the ROS measurements, 200-frame images were acquired at 20 Hz. Each localization event was fitted to a Gaussian function to determine the molecular centroid positions, followed by drift correction and reconstruction of the final super-resolution image.

For two-color imaging of γH2AX and PARP-1, sequential imaging was performed using the red and green channels. To accurately align the channels, fluorescent beads were first imaged within the same ROI and used for calibration. Organelles labeled with AF647 were imaged in the 647 nm channel using a 647 nm excitation laser, and AF488-labeled organelles were imaged in the 488 nm channel using a 488 nm excitation laser. The fluorescence signals were spectrally separated using emission filters and mirrors, and subsequently detected using an EMCCD camera. Two-color STORM images were merged using a custom-built MATLAB code, in which the calibration data obtained from the beads were applied to match the localizations from both channels (*48*).

For time-resolved STORM imaging, cells were UV-irradiated and subsequently fixed at multiple defined postirradiation time points (0, 0.25, 0.5, 1, 3, 6, 12, and 24 hours). At each time point, STORM images were acquired from 12 individually prepared biological replicates, and the mean values of each molecular readout, including ROS levels, γH2AX cluster size, PARP-1 cluster number, NF-κB nuclear-to-cytoplasmic localization density ratio, and IL-6 and IL-8 cluster number, were quantified and plotted as a function of postirradiation time to reconstruct the temporal progression of UV-induced molecular signaling.

### TEM imaging

For TEM imaging, HDFs were initially fixed with 2.5% (v/v) GA (16220; Electron Microscopy Sciences) in 0.1 M cacodylate buffer (pH 7.4) (C0250; Sigma-Aldrich) at 4°C overnight. After fixation, the samples were washed three times with 0.1 M cacodylate buffer on ice, followed by postfixation with 2% (v/v) osmium tetroxide (19190; Electron Microscopy Sciences) and 1.5% (w/v) potassium ferrocyanide (P9387; Sigma-Aldrich) for 1 hour. The samples were then washed three times with DW on ice and treated with 1% (w/v) thiocarbohydrazide (20753; Thermo Fisher Scientific) for 20 min at RT using a 0.22 μm-filtered solution. After washing thrice with DW at RT, the samples were postfixed with 2% osmium tetroxide in DW for 30 min at RT. Subsequently, the samples were washed three times with DW and incubated overnight at 4°C in a 1% uranyl acetate substitute (uranyLess; 22409, Electron Microscopy Sciences). After three washes with DW at RT, en bloc staining was performed by treatment with lead aspartate, prepared by mixing aspartic acid (BP374–100; Thermo Fisher Scientific) and lead nitrate (17900; Electron Microscopy Sciences) for 30 min at 60°C, followed by three final washes with DW at RT. After en bloc staining, the samples were dehydrated using a graded ethanol series (1.00983; Merck). Following dehydration, the samples were infiltrated with Epon 812 resin (14120; Electron Microscopy Sciences) diluted in ethanol via a stepwise increase in resin concentration. Ultrathin sections of approximately 70 nm thickness were cut from the polymerized resin blocks using an EM UC7 ultramicrotome (Leica, Austria) and placed on 150-mesh copper grids. The sections on the grids were stained with uranyLess and lead citrate (22410; Electron Microscopy Sciences), and subsequently imaged using a KBSI Bio-TEM system (JEM-1400 Plus at 120 kV; JEOL).

For TEM imaging with the reconstructed human skin tissue, the sample was fixed in 2.5% glutaraldehyde dissolved in 0.1 M cacodylate buffer (pH 7.4) at 4°C overnight. The fixed samples were rinsed with the same buffer and then postfixed in 2% osmium tetroxide prepared in 0.1 M cacodylate buffer for 3 hours on ice. After three washes with cacodylate buffer, the samples were dehydrated through a graded ethanol series and infiltrated with Epon 812 resin by stepwise replacement of propylene oxide (20401; Electron Microscopy Sciences) with the resin. Samples were embedded in pure Epon 812 and polymerized to obtain resin blocks. Ultrathin sections (∼70 nm) were prepared using an EM UC7 ultramicrotome (Leica, Austria), collected on 150-mesh copper grids, and contrast-stained with uranyl acetate and lead citrate. TEM images were acquired using a KBSI Bio-TEM system (JEM-1400 Plus at 120 kV, JEOL).

### Quantification from images

To quantitatively analyze the ultrastructural features of cellular organelles from STORM images, including mitochondria, ER, Golgi apparatus, and microtubule, we used the ‘SR-Tesseler’ software (*49*), which performs cluster analysis based on Voronoi diagrams of single-molecule localization microscopy data, as described in a previous study (*46*). Voronoi diagrams were generated to reconstruct the spatial distribution of the signals using the intensity and frame information obtained from the STORM images of each organelle. To reduce background noise and isolate the structural features of the organelles, we optimized the object segmentation parameters, primarily the density factor and minimum area, using the ‘SR-Tesseler’ software (*49*).

For mitochondrial quantification, the ‘Mitochondria Analyzer’ plug-in in ImageJ was used, in which mitochondria were identified from the skeletonized ‘SR-Tesseler’-processed STORM images (*17*, *50*). The length of the longest mitochondrion in each image was measured, and the average length across the entire image was used to compare the mitochondrial morphology under different experimental conditions.

To analyze ER ultrastructure, we used the built-in ‘AnalyzER’ application in MATLAB, which classifies structures based on pixel-level intensity profiles from ‘SR-Tesseler’-processed STORM images, as described in the previous study (*17*, *51*). The tubular and sheet structures of the ER were distinguished by their full width at half maximum (FWHM); structures with FWHM values above a defined threshold were classified as sheets, those in an intermediate range as tubules, and those below a minimum threshold as background. Based on this classification, the ratio of ER tubules to sheet structures was calculated. The area percentage of each structure type was quantified using the ‘Area Fraction’ tool in the ImageJ.

To quantify the number and spatial distribution of the Golgi apparatus fragments, we used a custom-written MATLAB code, as described in a previous study (*17*). STORM images exported from ‘SR-Tesseler’ were first converted to an 8-bit grayscale format using ImageJ. The code then applies Otsu’s method to binarize the Golgi apparatus image and identify the fragments using connected component analysis. The nucleus was binarized from the corresponding image, and its centroid was automatically detected. The Euclidean distance between each pixel of the identified Golgi fragments and the nuclear centroid was calculated to quantify the number of Golgi fragments and their spatial distribution relative to the nucleus.

For microtubule quantification, microtubule filaments were extracted from the ‘SR-Tesseler’–processed STORM images. These filaments were then converted into skeletonized representations using the ‘Analyze Skeleton’ plug-in in ImageJ (*52*). The length of each microtubule was measured, and the mean filament length per cell was calculated to consider UV-induced microtubule shortening and recovery under different experimental conditions.

For MTOC quantification, localization data exported from the STORM images of the MTOC were analyzed. Microtubules extending from each MTOC were treated as linear structures, and the localization density was calculated by dividing the number of localizations by the filament length. The mean localization density across all filaments was used to evaluate the UV-induced disruption of the MTOC structure.

For ROS quantification, the contrast values of the ROS fluorescence were extracted from raw diffraction-limited image data. The mean contrast within the defined cellular ROIs was calculated and used as the ROS level. These values were used to compare relative ROS signal intensities across the experimental conditions.

To quantify NF-κB activation in the nucleus, the nuclear-to-cytoplasmic (N/C) localization density ratio was calculated as previously reported (*53*). Single-molecule localization coordinates were extracted from the STORM images and analyzed using a custom MATLAB script. The number of localizations detected within the nucleus and cytoplasm was divided by the corresponding area of each region to calculate the localization density. The N/C ratio was calculated by dividing the nuclear localization density in the nucleus by that in the cytoplasm.

To quantify the colocalization between γH2AX and PARP-1 in two-color STORM images, we employed a previously reported custom-written MATLAB code (*54*). For each STORM image, γH2AX and PARP-1 clusters were first independently identified within the nuclear region. The pixel overlap ratio between the two channel images was then calculated and defined as the colocalization percentage. To validate the significance of the measured colocalization, the centroid positions of γH2AX clusters were randomly redistributed within the nuclear area to generate ten randomized images. The mean colocalization percentage obtained from these randomized images was subsequently compared with that of the original two-color STORM image of γH2AX and PARP-1.

To quantify the intracellular collagen and extracellular collagen fibrils, different analysis approaches were applied according to their structural characteristics. For intracellular collagen, multiple 1 μm × 1 μm ROIs were defined in each STORM image, and the localization density within each ROI was calculated and averaged. For extracellular collagen fibrils, several regions were selected for filament analysis, and the filament length density within each region was measured and averaged for each sample.

To quantify the fluorescence intensity of CFSE-labeled EVs in confocal images, background-corrected fluorescence intensities were calculated by subtracting the mean fluorescence intensity of cell-free regions from that of cellular regions containing CFSE-labeled EVs. Image analysis was performed using ImageJ. Multiple 50 × 50 pixel ROIs were selected from both cell-containing and cell-free areas in each image. The mean fluorescence intensity was measured for each ROI, and the averaged values were used to calculate the background-corrected fluorescence intensity. For STORM image analysis, the localization density of CFSE-labeled EVs internalized by HDF cells was quantified within 2 μm × 2 μm ROIs in STORM images.

For quantitative analysis of confocal images of the reconstructed human skin tissue, mitochondrial circularity and aspect ratio were determined using the ‘Analyze Particles’ in ImageJ. Mitochondrial length and microtubule length were quantified using the same analysis procedures described for STORM image analysis.

To quantify LivePAR nuclear enrichment and PAR cluster formation, nuclear ROIs were defined from Hoechst-stained images and applied to the corresponding GFP-channel images using ImageJ. The mean nuclear LivePAR-GFP fluorescence intensity was measured at each time point and normalized to the pre-UV baseline intensity for each cell. Cells with weak GFP expression, saturated GFP signal, or substantial movement during time-lapse acquisition were excluded from analysis. Normalized nuclear GFP intensity over time was used to evaluate UV-induced LivePAR nuclear enrichment. For PAR cluster analysis, GFP fluorescence images were thresholded to exclude background signal before quantitative analysis of PAR clusters. The number and size of PAR clusters were subsequently quantified using the ‘Analyze Particles’ in ImageJ.

For FRAP analysis, fluorescence recovery within the photobleached ROI was quantified using ImageJ. The mean fluorescence intensity of the bleached ROI was measured at each time point using the ‘Multi Measure’ in ImageJ. Fluorescence intensities were normalized to the mean fluorescence intensity measured from the pre-bleach images to generate recovery curves. The fluorescence recovery half-time was determined by fitting the normalized recovery curves to a single-exponential recovery model.

### Cluster analysis from STORM images

To identify the clusters of IL-6, IL-8, γH2AX, and PARP-1 from STORM images, the density-based spatial clustering of applications with noise (DBSCAN) algorithm was used by employing the “sklearn.cluster.DBSCAN” module in Python (version 3.12.0) (*55*–*57*). Twelve individual cell images were analyzed for each experimental condition. DBSCAN parameters, including eps (ε) and min_samples_, were fixed before group comparison and identical parameter values were applied to all images within a given analysis. The min_samples_ value was chosen to distinguish genuine biological clusters from randomly distributed background localizations. While parameters were optimized on an image-by-image basis to account for variations in localization density across cells, the same parameter values were consistently applied to all images within each experimental group. After DBSCAN-based cluster identification, the γH2AX clusters were further analyzed using a custom MATLAB script (MathWorks, R2021a) to perform principal component analysis (PCA), aligning each cluster along its principal axes, as described in a previous study (*56*). The spatial distributions along the PCA-aligned axes were used to calculate the FWHM, and the mean γH2AX cluster size was defined as the average FWHM of the major and minor axes, computed using a custom-written Python script. After DBSCAN clustering, quantitative analyses of IL-6, IL-8, and PARP-1 were performed by counting the number of clusters identified for each experimental condition.

Ripley’s L function was used to quantify the nanoscale clustering behavior of PARP-1 clusters. After defining the nuclear ROI, the mean localization density (λ) in the nucleus was determined by dividing the number of localizations by the corresponding ROI area. Ripley’s K, L, and H functions were calculated over a range of distance values (*r*) to examine PARP-1 clustering at various spatial scales. To avoid unreliable results, r values larger than one-quarter of the smallest ROI dimension were omitted. For each ROI, all pairwise distances between localizations were calculated. To minimize boundary effects, localizations close to the ROI boundary were excluded when their distance to the edge was smaller than *r*. Only edge-corrected localizations were used for Ripley’s function calculations, and ROIs with fewer than 10 remaining localizations were disregarded. The spatial distribution of PARP-1 localizations was treated as a two-dimensional point pattern. For each nucleus, the number of neighboring localizations within a distance *r* from each point was calculated, and Ripley’s K-function was defined as (*58*, *59*) $K\left(r\right)=\frac{1}{n}\sum_{i=1}^{n}N_{i}\left(r\right)/\lambda$, where *n* is the total number of localizations, $N_{i}\left(r\right)$ is the number of neighboring localizations within distance *r* of the *i-*th localization, and λ is the mean localization density within the nuclear ROI. This K-function was transformed into the L- and H-functions as $L\left(r\right)=\sqrt{K\left(r\right)/\pi }$ and H(r)=L(r)−r. For a spatially random distribution, the expected value of H(r) is zero. Rather than applying a specific numerical threshold, clustering was defined by the deviation of H(r) from zero, where H(r) > 0 indicates clustering at a given distance scale *r*. The peak position of the H(r) curve was used to determine the characteristic cluster scale, following the previously described approach (*59*). Positive H(r) values indicate clustering relative to a spatially random distribution, whereas values near zero or negative reflect random or dispersed spatial organizations, respectively.

To further characterize the clustering behavior of PARP-1 beyond point-based clustering metrics, NND analysis was performed. For clustering, DBSCAN was first applied with a fixed neighborhood radius (ε) and a minimum number of points (MinPts), enabling the identification of spatially dense PARP-1 clusters while excluding sparse localizations that were classified as noise. Only the clusters identified by DBSCAN were included in the subsequent analyses. For each detected cluster, the centroid position was calculated as the mean of the localization coordinates of that cluster. Cluster-cluster NNDs were then computed by measuring the Euclidean distances between all cluster centroids and identifying the distance to the nearest neighboring cluster. ROIs containing fewer than two clusters were excluded from the NND analysis.

### Statistical analysis

Quantitative analyses were performed using data obtained from 12 independent measurements for each experimental condition (n = 12) as indicated in the respective figure legends. Each independent measurement corresponds to a single cell image acquired from a separately prepared biological replicate, where cells were cultured, treated, and fixed on different days under identical experimental conditions. This ensures that the reported values reflect genuine biological variability rather than technical replicates from a single preparation. No data normalization, transformation, or outlier exclusion was applied at any stage of analysis. All results are presented as mean ± standard error of the mean. Statistical significance was evaluated using one-way analysis of variance (ANOVA) followed by Tukey’s multiple-comparisons test. Statistical significance thresholds were defined as *P <* 0.05 (*), < 0.01 (**), and < 0.001 (***), whereas non-significant differences are denoted as “ns.” Statistical analyses, trend-line fitting, data visualization, and graph generation were performed using OriginPro 2016 software (OriginLab).
