# Supplementary material for: Super-resolution mapping of nuclear stress signaling by probiotic-derived extracellular vesicles in UV-induced skin photodamage
Source: Sci Adv. 2026 Oct 30;12(44):eaek5197. doi: 10.1126/sciadv.aek5197 (PMC13721989; doi:10.1126/sciadv.aek5197)
Supplement: Supplementary file 1 [file sciadv.aek5197_sm.pdf]

Science Advances
AAAS

Supplementary Materials for

Super-resolution mapping of nuclear stress signaling by probiotic-derived extracellular vesicles in UV-induced skin photodamage

Ga-eun Go *et al.*

Corresponding author: Doory Kim, doorykim@hanyang.ac.kr

Sci. Adv. **12**, eaek5197 (2026)
DOI: 10.1126/sciadv.aek5197

This PDF file includes:

Figs. S1 to S27

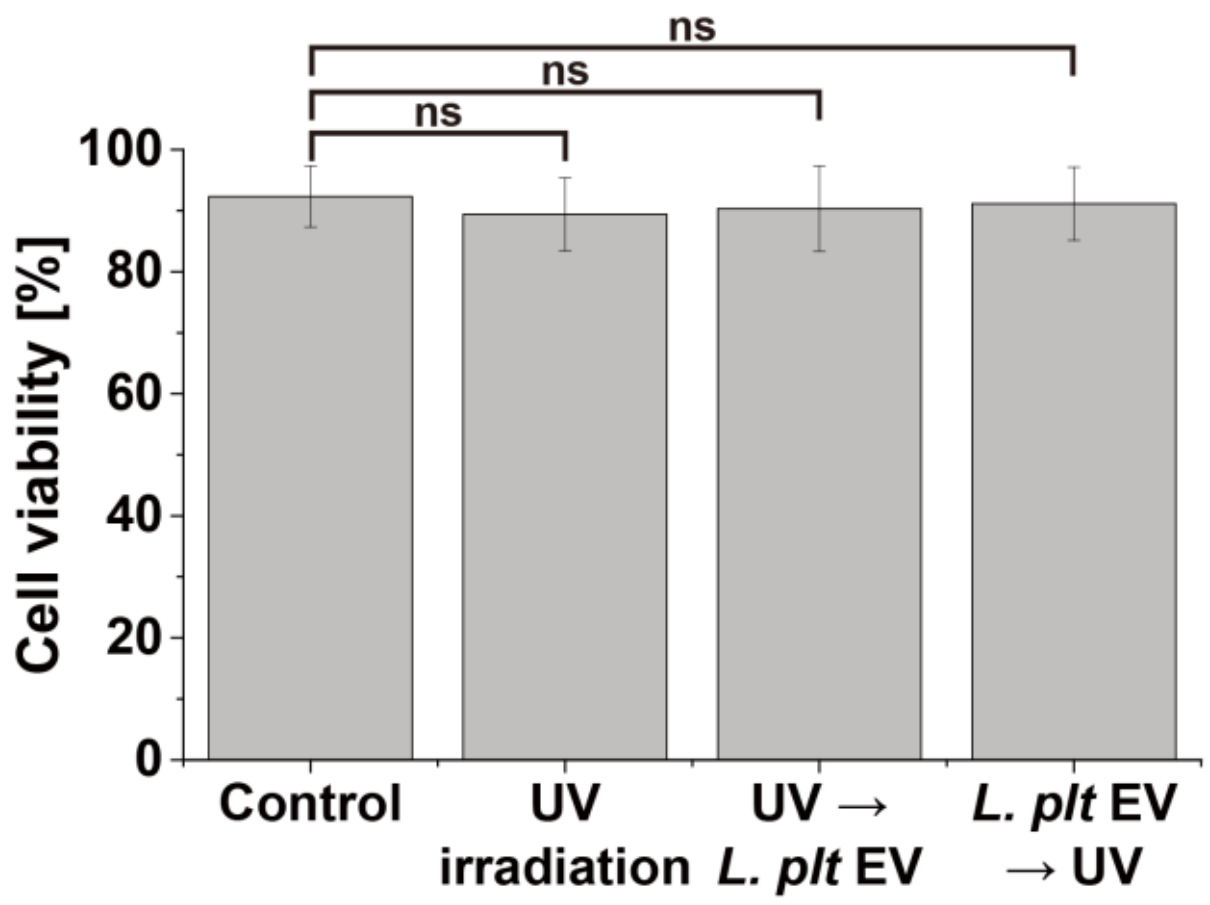

Fig. S1. Cell viability of HDF cells following UV irradiation and *L. plantarum* EV treatment. Cell viability was assessed by Trypan blue exclusion assay under four conditions: untreated control, UV-irradiated, UV-irradiated with *L. plantarum* EV post-treatment, and *L. plantarum* EV pre-treatment followed by UV irradiation. Data are presented as mean ± SEM from twelve independent experiments. One-way ANOVA with Tukey's post hoc test, ns: $P > 0.05$ compared to the control.

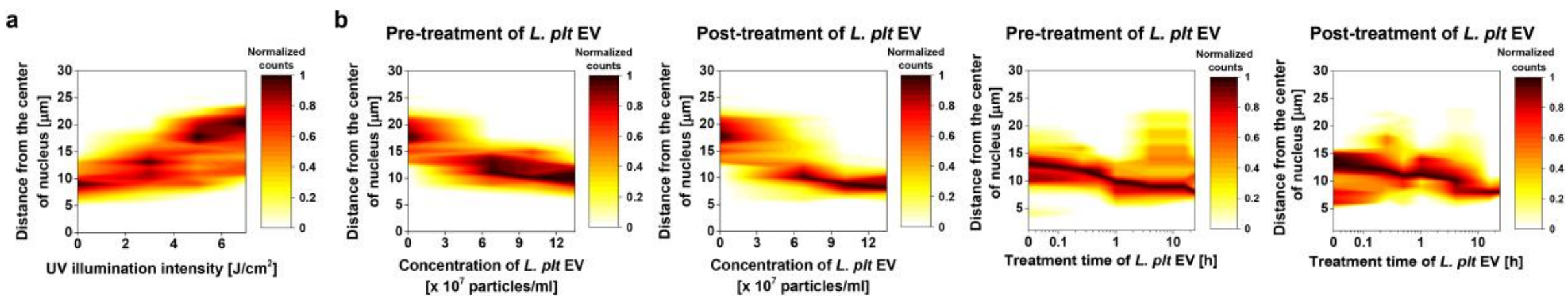

Fig. S2. Quantitative analysis of Golgi dispersal in UV-irradiated and *L. plantarum* EV-treated HDF cells. Heatmaps showing the spatial distribution of Golgi fragments as a function of distance from the nuclear centroid, corresponding to the Golgi fragmentation data presented in **(a)** Fig. 1C and **(b)** Fig. 2C, respectively. Data are presented as mean ± SEM from twelve independent experiments.

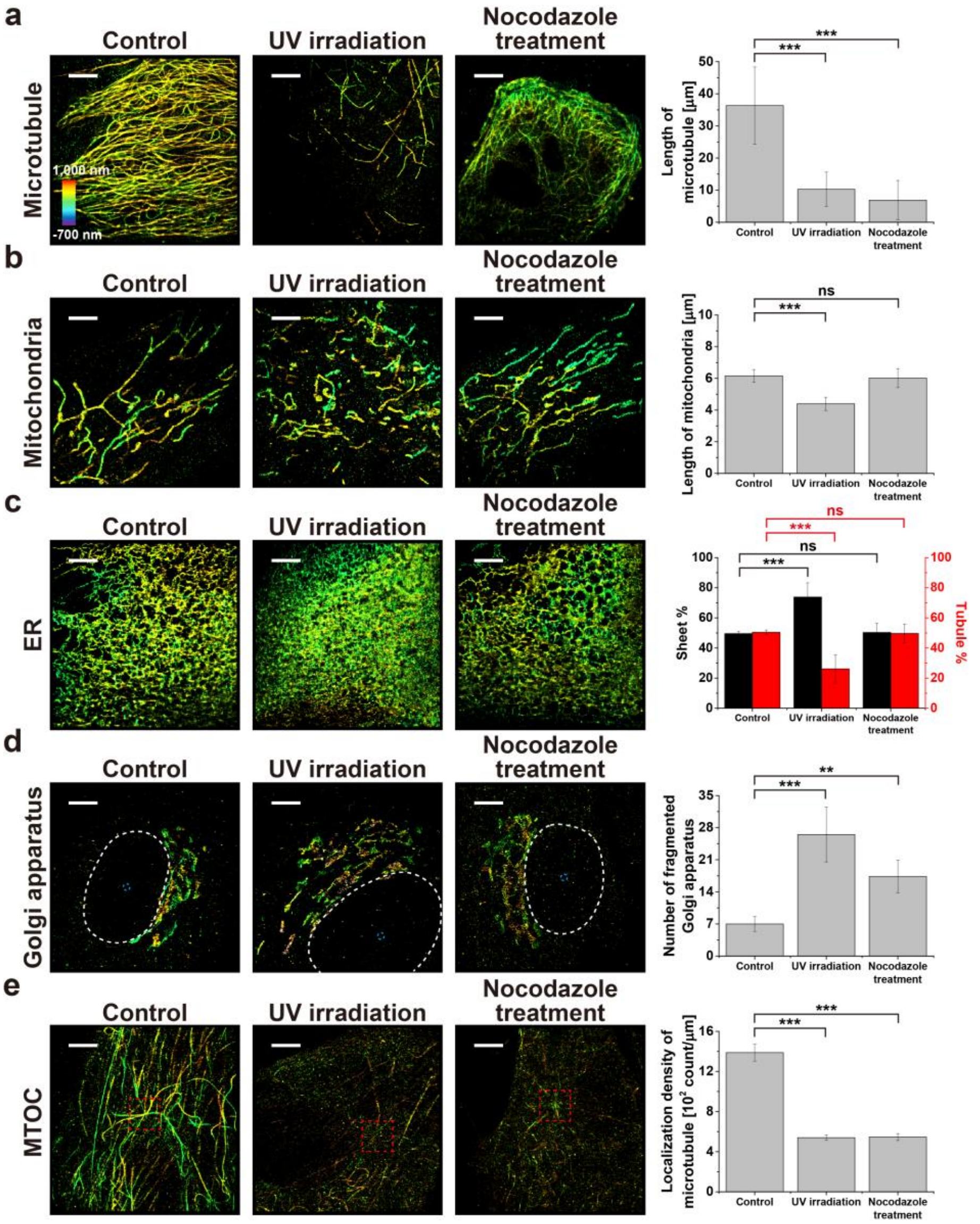

Fig. S3. Effect of nocodazole-induced microtubule depolymerization on organelle ultrastructure. Representative STORM images of **(a)** microtubules, **(b)** mitochondria, **(c)** ER, **(d)** Golgi apparatus, and **(e)** MTOC in untreated control, UV-irradiated, and nocodazole-treated HDF cells. Quantitative analyses of organelle morphology under each condition as a function of nocodazole treatment are presented in the graphs on the right: (a) microtubule length, (b) mitochondrial length, (c) relative ratio of tubular or sheet-like ER structure, (d) number of Golgi fragments, and (e) localization density of MTOC. White dashed line: nuclear boundary. Red dashed line: MTOC. Scale bars, 5 μm. Data are presented as mean ± SEM from twelve independent experiments. One-way ANOVA with Tukey's post hoc test, ns: $P > 0.05$, $^{**}P < 0.01$, $^{***}P < 0.001$ compared to the control.

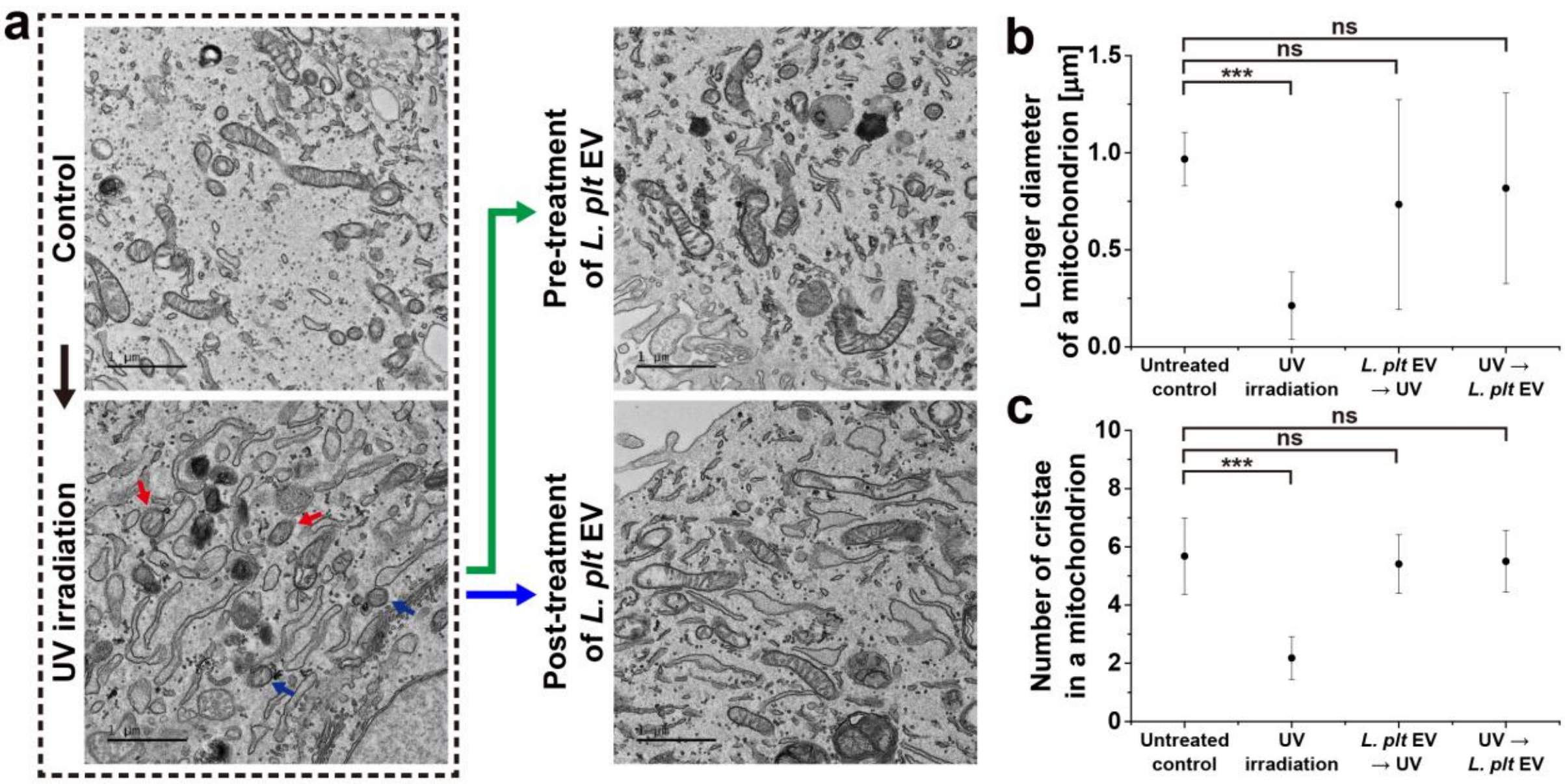

Fig. S4. Transmission electron microscopy (TEM) analysis of mitochondrial ultrastructure in UV-irradiated and *L. plantarum* EV-treated HDF cells. **(a)** Representative TEM images of mitochondria under four conditions: untreated control, UV-irradiated, and UV-irradiated with either pre- or post-treatment with *L. plantarum* EVs. Red arrows: mitochondrial fission. Blue arrows: cristae loss. Scale bars, 1 μm. Quantitative analyses of **(b)** mitochondrial longer diameter and **(c)** number of cristae per mitochondrion are shown on the right. Data are presented as mean ± SEM from twelve independent experiments. One-way ANOVA with Tukey's post hoc test, ns: $P > 0.05$, $^{***}P < 0.001$ compared to the control.

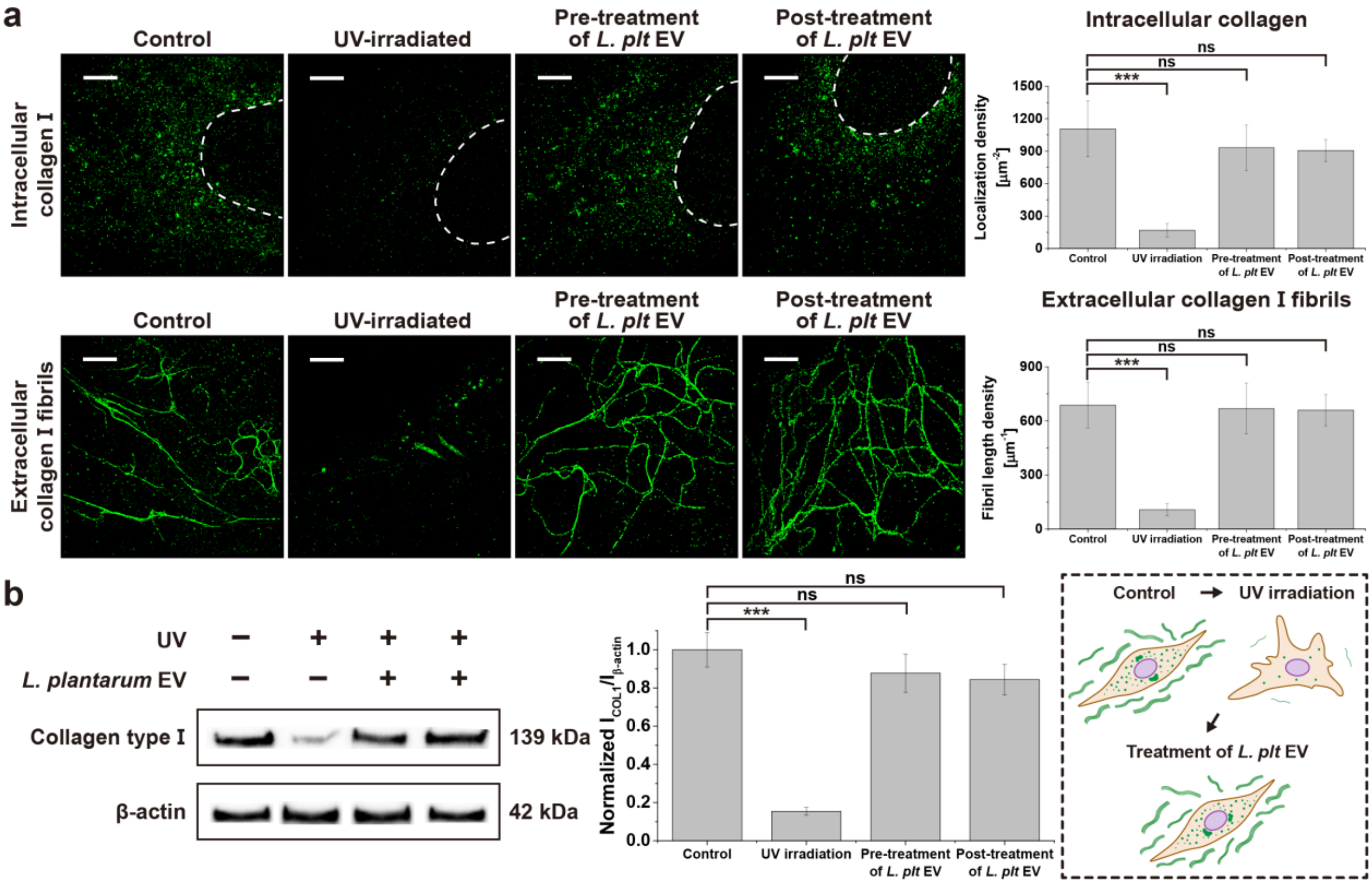

Fig. S5. *L. plantarum* EV treatment restores type I collagen in UV-photodamaged human dermal fibroblasts. (a) Representative STORM images of type I collagen in control, UV-irradiated, *L. plantarum* EV-pretreated, and EV-post-treated HDF cells. The upper panels show intracellular collagen I, with the nuclear boundaries indicated by white dashed lines, whereas the lower panels show extracellular collagen I fibrils surrounding the cells. Quantification of intracellular collagen I as the number of localizations per unit area and extracellular collagen I fibrils as the total fibril length per unit area is shown on the right. Scale bars, 5 μm. (b) Representative Western blot analysis of collagen I in HDF cells under the indicated conditions. Quantification of collagen I expression normalized to β-actin is shown on the right. The schematic illustrates collagen I in the untreated control, the UV-induced loss of collagen I and its restoration following *L. plantarum* EV treatment. Data are presented as mean ± SEM from twelve independent experiments. One-way ANOVA with Tukey's post hoc test, ns: $P > 0.05$, ***$P < 0.001$ compared to the control.

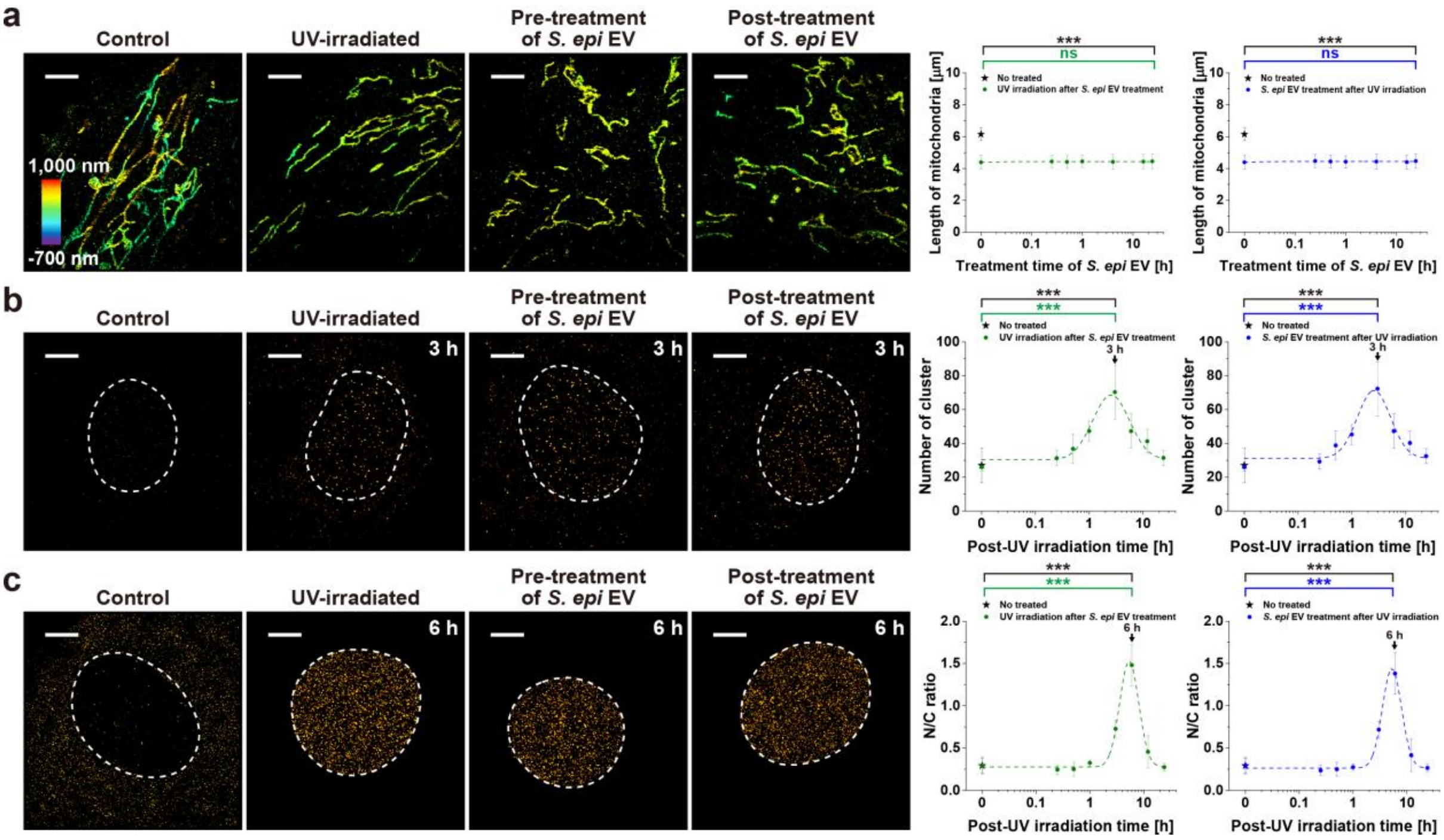

Fig. S6. Evaluation of *S. epidermidis* EVs as a bacterial EV specificity control in UV-photodamaged HDF cells. Representative STORM images of mitochondria (a), PARP-1 (b), and NF-κB (c) in untreated control, UV-irradiated, *S. epidermidis* EV-pretreated, and *S. epidermidis* EV-post-treated HDF cells. Quantitative analysis of mitochondrial length (a), PARP-1 cluster number (b), and nuclear-to-cytoplasmic (N/C) ratio of NF-κB localization (c) over the indicated EV treatment times is shown on the right. White dashed lines indicate nuclear boundaries. Scale bars, 5 µm. Data are presented as mean ± SEM from twelve independent experiments. One-way ANOVA with Tukey's post hoc test, ns: $P > 0.05$, ***$P < 0.001$ compared to the control.

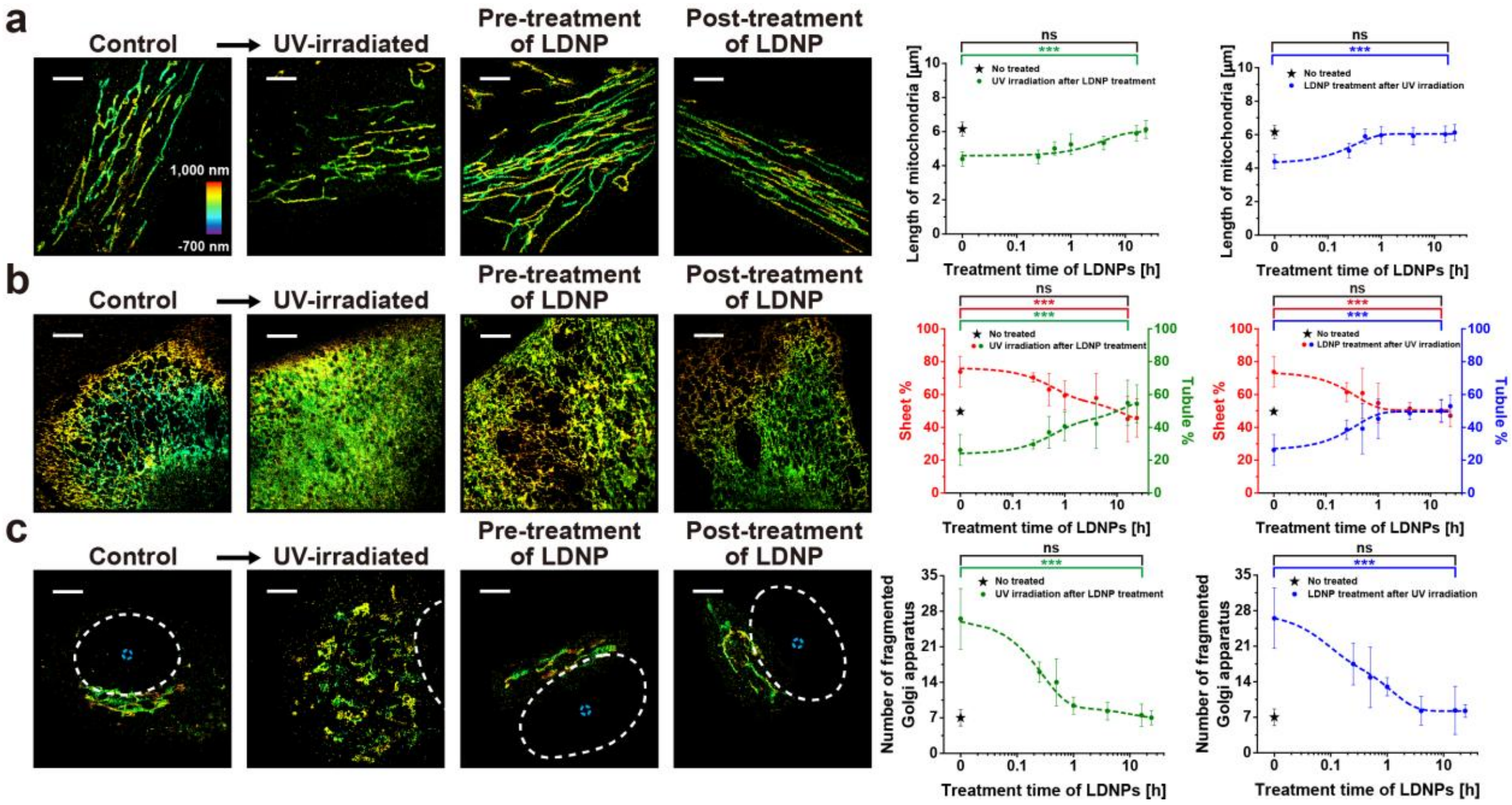

Fig. S7. Protective and restorative effects of *L. plantarum*-derived nanoparticles (LDNPs) on UV-damaged organelles in HDF cells. Representative STORM images of **(a)** mitochondria, **(b)** endoplasmic reticulum (ER), and **(c)** Golgi apparatus under four experimental conditions: untreated control, UV-damaged cells, cells pretreated with LDNPs before UV exposure, and cells treated with LDNPs after UV damage. Quantitative analyses of organelle morphology as a function of LDNPs treatment duration are presented in the graphs on the right: (a) mitochondrial length, (b) relative ratio of tubular or sheet-like ER structure, and (c) number of Golgi fragments. White dashed line: nuclear boundary. Scale bars, 5 μm. Data are presented as mean ± SEM from twelve independent experiments. One-way ANOVA with Tukey's post hoc test, ns: $P > 0.05$, $^{***}P < 0.001$ compared to the control.

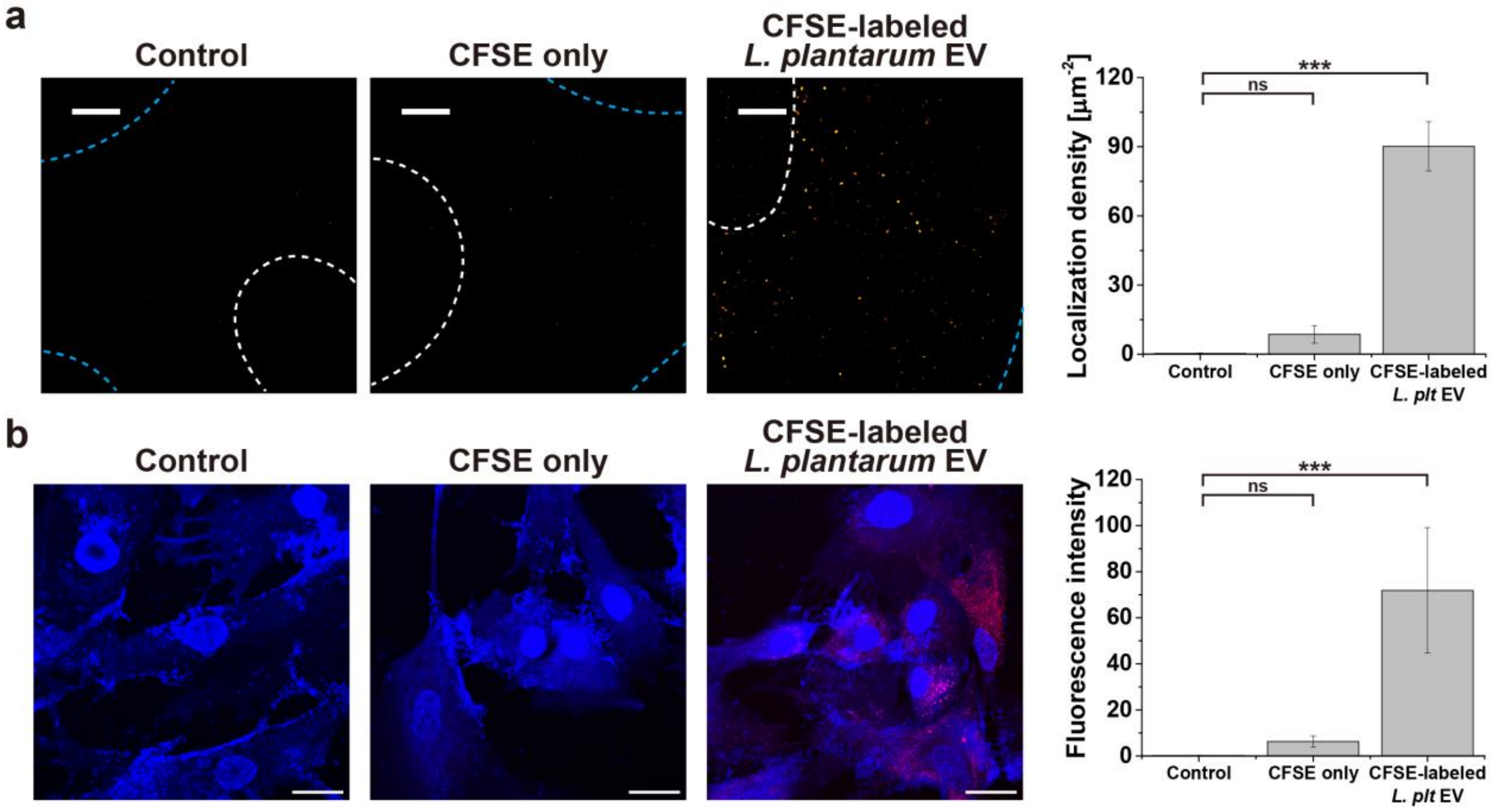

Fig. S8. Cellular internalization of CFSE-labeled *L. plantarum* EVs in HDF cells. **(a)** Representative STORM images of HDF cells under three conditions: untreated control, dye-only treated, and CFSE-labeled *L. plantarum* EV-treated cells. Quantitative analysis of intracellular localization density under each condition is shown on the right. **(b)** Representative confocal images of HDF cells under the same three conditions, together with quantitative analysis of intracellular fluorescence intensity shown on the right. In both (a) and (b), the progressive increase in intracellular signal in CFSE-labeled EV-treated cells, compared to the untreated control and dye-only conditions, confirms genuine cellular internalization of *L. plantarum* EVs rather than surface adhesion or free dye contamination. White dashed line: nuclear boundary. Blue dashed line: cell boundary. Scale bars, 5 μm in (a) and 30 μm in (b). Data are presented as mean ± SEM from twelve independent experiments. One-way ANOVA with Tukey's post hoc test, ns: $P > 0.05$, ***$P < 0.001$ compared to the control.

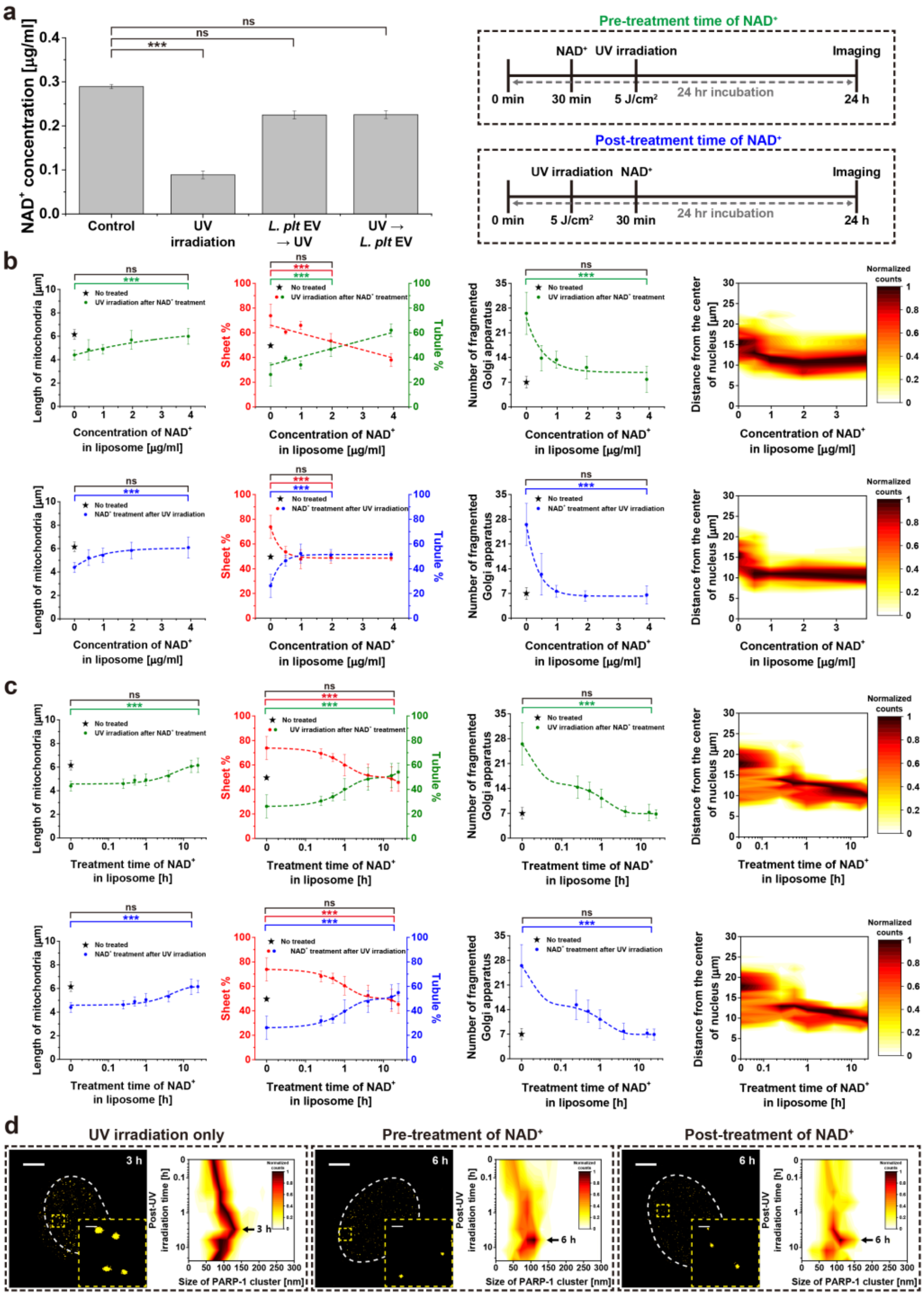

Fig. S9. NAD+ as a functional mediator of *L. plantarum* EV-mediated anti-photodamage effects. **(a)** Intracellular NAD+ levels in HDF cells under four conditions: untreated control, UV-irradiated, and UV-irradiated with either pre- or post-treatment with *L. plantarum* EVs, as measured by NAD+/NADH quantification assay. **(b and c)** Quantitative analyses of organelle morphology in UV-photodamaged HDF cells treated with NAD+-loaded liposomes as a function of NAD+ concentration (b) and treatment duration (c), under both pre- and post-treatment conditions. For each organelle, the following parameters were quantified: mitochondrial length, relative proportions of tubular and sheet-like ER structures, number of Golgi fragments, and distance of Golgi fragments from the nuclear centroid. **(d)** Representative STORM images of PARP-1 at peak time points after UV irradiation under UV-irradiation only, NAD+ pre-treatment, and NAD+ post-treatment conditions, together with heatmaps illustrating the time-dependent progression of PARP-1 cluster number across all three conditions. White dashed line: nuclear boundary. Scale bars, 5 μm in (d) and 500 nm in the yellow dashed box region in (d). Data are presented as mean ± SEM from twelve independent experiments. One-way ANOVA with Tukey's post hoc test, ns: $P > 0.05$, $***P < 0.001$ compared to the control.

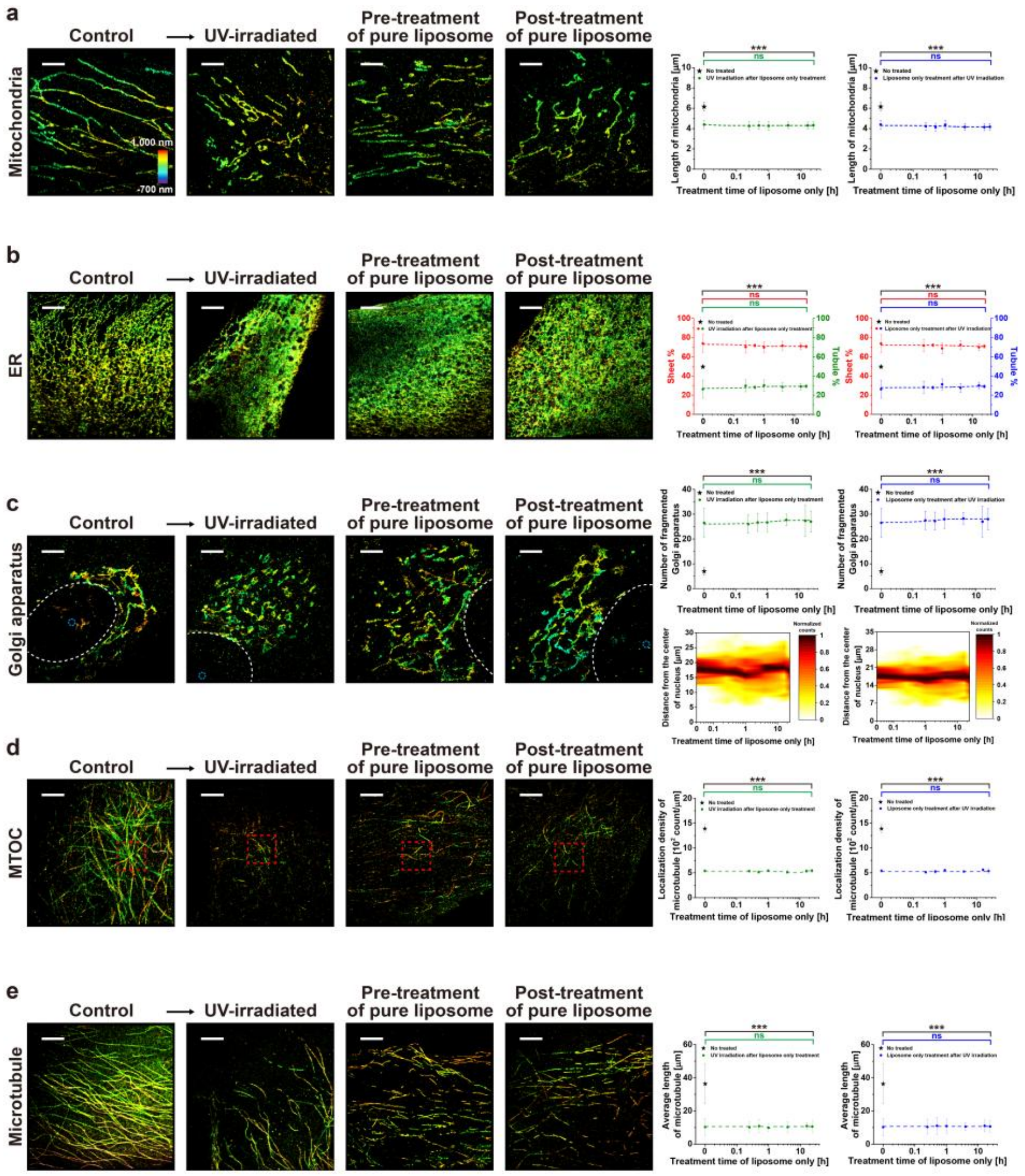

Fig. S10. Empty liposome treatment does not restore UV-induced organelle damage in HDF cells. Representative STORM images of **(a)** mitochondria, **(b)** endoplasmic reticulum (ER), **(c)** Golgi apparatus, **(d)** microtubule organizing center (MTOC), and **(e)** microtubules under four experimental conditions: untreated control, UV-irradiated, and UV-irradiated with either pre- or post-treatment with NAD^+-free empty liposomes. Quantitative analyses of organelle morphology as a function of treatment duration are presented in the graphs on the right: (a) mitochondrial length, (b) relative proportions of tubular and sheet-like ER structures, (c) number of Golgi fragments and distance of Golgi fragments from the nuclear centroid, (d) localization density of MTOC, and (e) microtubule length. Unlike *L. plantarum* EV treatment, empty liposome treatment did not restore UV-induced structural alterations in any of the organelles examined regardless of treatment duration, confirming that the protective effects of *L. plantarum* EVs are attributable to their bioactive cargo rather than to the lipid vesicle carrier. White dashed line: nuclear boundary. Scale bars, 5 μm. Data are presented as mean ± SEM from twelve independent experiments. One-way ANOVA with Tukey's post hoc test, ns: $P > 0.05$, ***$P < 0.001$ compared to the control.

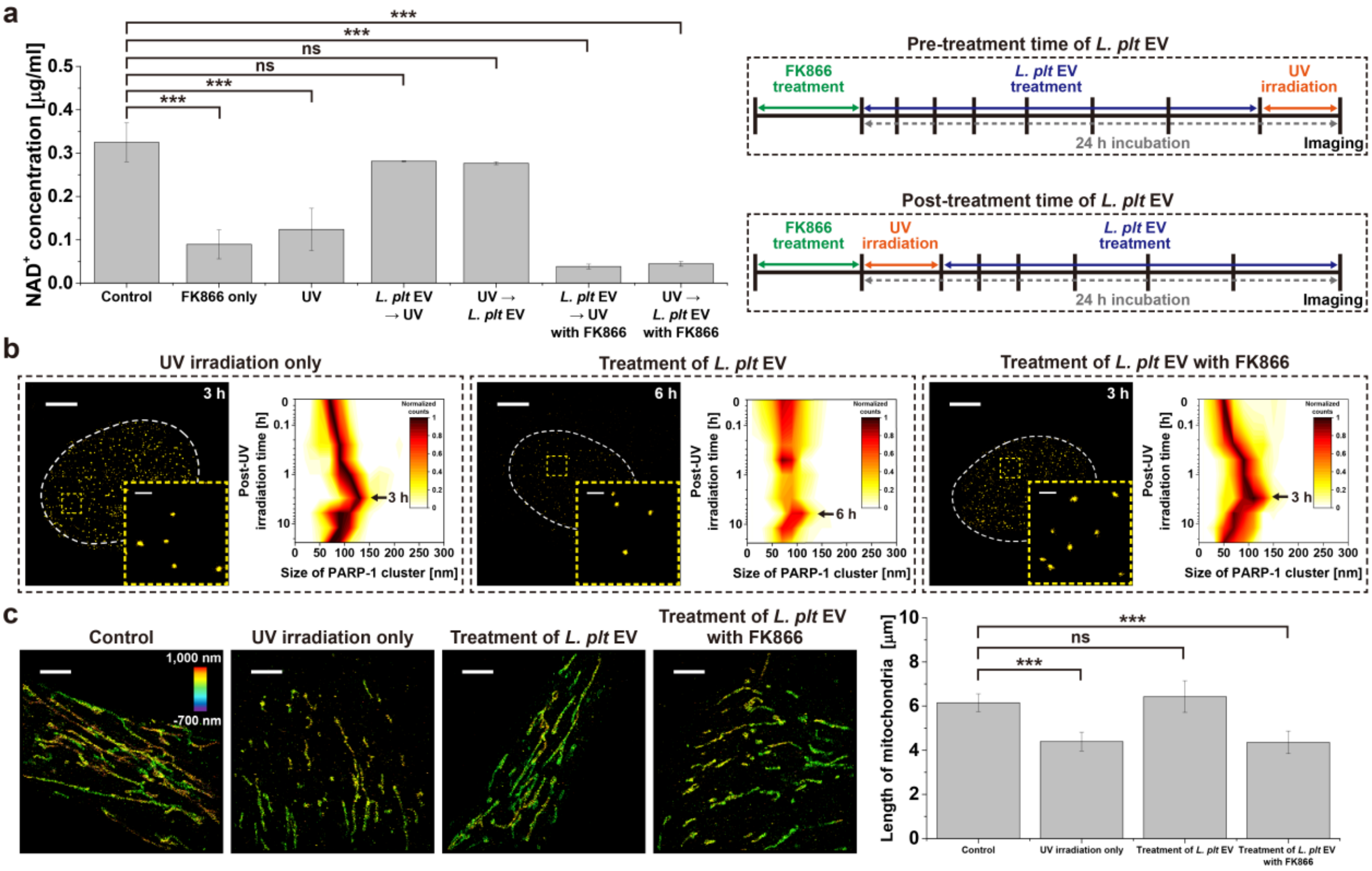

Fig. S11. FK866-mediated NAD⁺ depletion impairs the protective effects of *L. plantarum* EVs in UV-photodamaged HDF cells. (a) Intracellular NAD⁺ levels in HDF cells under the following conditions: untreated control, FK866 treatment alone, UV irradiation alone, *L. plantarum* EV pre-treatment followed by UV irradiation, UV irradiation followed by *L. plantarum* EV post-treatment, *L. plantarum* EV pre-treatment followed by UV irradiation in the presence of FK866, and UV irradiation followed by *L. plantarum* EV post-treatment in the presence of FK866. UV irradiation reduced intracellular NAD⁺ levels, whereas both EV pre- and post-treatment substantially restored NAD⁺ levels. This EV-mediated restoration was largely abolished by FK866 pretreatment. Schematics on the right illustrate the experimental timelines for EV pre- and post-treatment with FK866. (b) Representative STORM images and time-dependent cluster-size distributions of PARP-1 following UV irradiation under the indicated treatment conditions. The data for the UV irradiation-only condition are reproduced from Fig. S9d for comparison. In UV-only cells, PARP-1 clustering reached a maximum at approximately 3 h, whereas *L. plantarum* EV treatment delayed the peak to approximately 6 h. FK866-mediated NAD⁺ depletion largely abolished this EV-induced delay, with PARP-1 clustering again peaking at approximately 3 h. White dashed lines indicate nuclear boundaries, and enlarged views of the boxed regions are shown in the insets. (c) Representative STORM images of mitochondria and quantitative analysis of mitochondrial length under the indicated treatment conditions. UV irradiation induced mitochondrial fragmentation, as indicated by a decrease in mitochondrial length, whereas *L. plantarum* EV treatment substantially restored mitochondrial length. This restorative effect was markedly impaired by FK866 pretreatment, indicating that EV-mediated protection of mitochondrial morphology depends on cellular NAD⁺ availability. Scale bars, 5 µm and 500 nm in the yellow dashed box region. Data are presented as mean ± SEM from twelve independent experiments. One-way ANOVA with Tukey's post hoc test, ns: $P > 0.05$, ***$P < 0.001$ compared to the control.

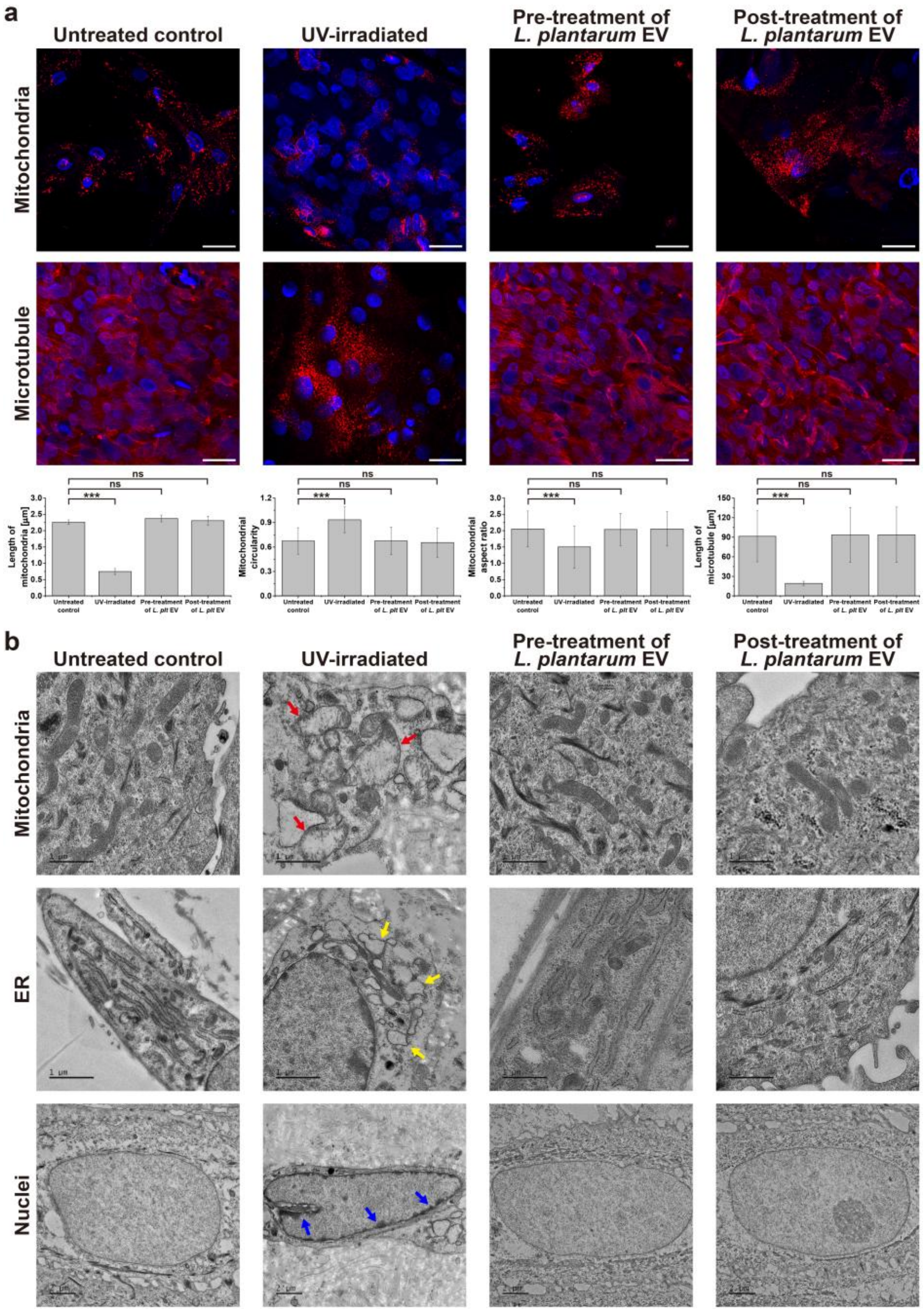

Fig. S12. Validation of the protective and restorative effects of *L. plantarum* EVs in a reconstructed human skin model. **(a)** Confocal fluorescence images of mitochondria (upper) and microtubules (lower) in a reconstructed human skin model under untreated control, UV-irradiated, *L. plantarum* EV pre-treatment, and *L. plantarum* EV post-treatment conditions. UV irradiation induced mitochondrial fragmentation, accompanied by decreased mitochondrial length, increased circularity, and decreased aspect ratio, as quantified in the graphs. UV exposure also caused pronounced microtubule depolymerization, whereas both pre- and post-treatment with *L. plantarum* EVs largely restored mitochondrial morphology and microtubule organization to levels comparable to the untreated control. Scale bars, 30 μm. Data are presented as mean ± SEM from twelve independent experiments. One-way ANOVA with Tukey's post hoc test, ns: $P > 0.05$, $^{***}P < 0.001$ compared to the control. **(b)** Representative TEM images of mitochondria (upper), ER (middle), and nuclei (lower) in reconstructed human skin sections under the indicated experimental conditions. UV irradiation induced mitochondrial fission accompanied by loss of cristae structure, marked swelling and dilation of the ER lumen, and heterochromatin condensation and nuclear membrane invagination. All three ultrastructural abnormalities were markedly alleviated following pre- or post-treatment with *L. plantarum* EVs, restoring tissue ultrastructure toward the untreated control. Red arrows: mitochondrial fission. Yellow arrows: ER swelling and dilation. Blue arrows: heterochromatin condensation. Scale bars, 1 μm for mitochondria and ER, and 2 μm for nuclei.

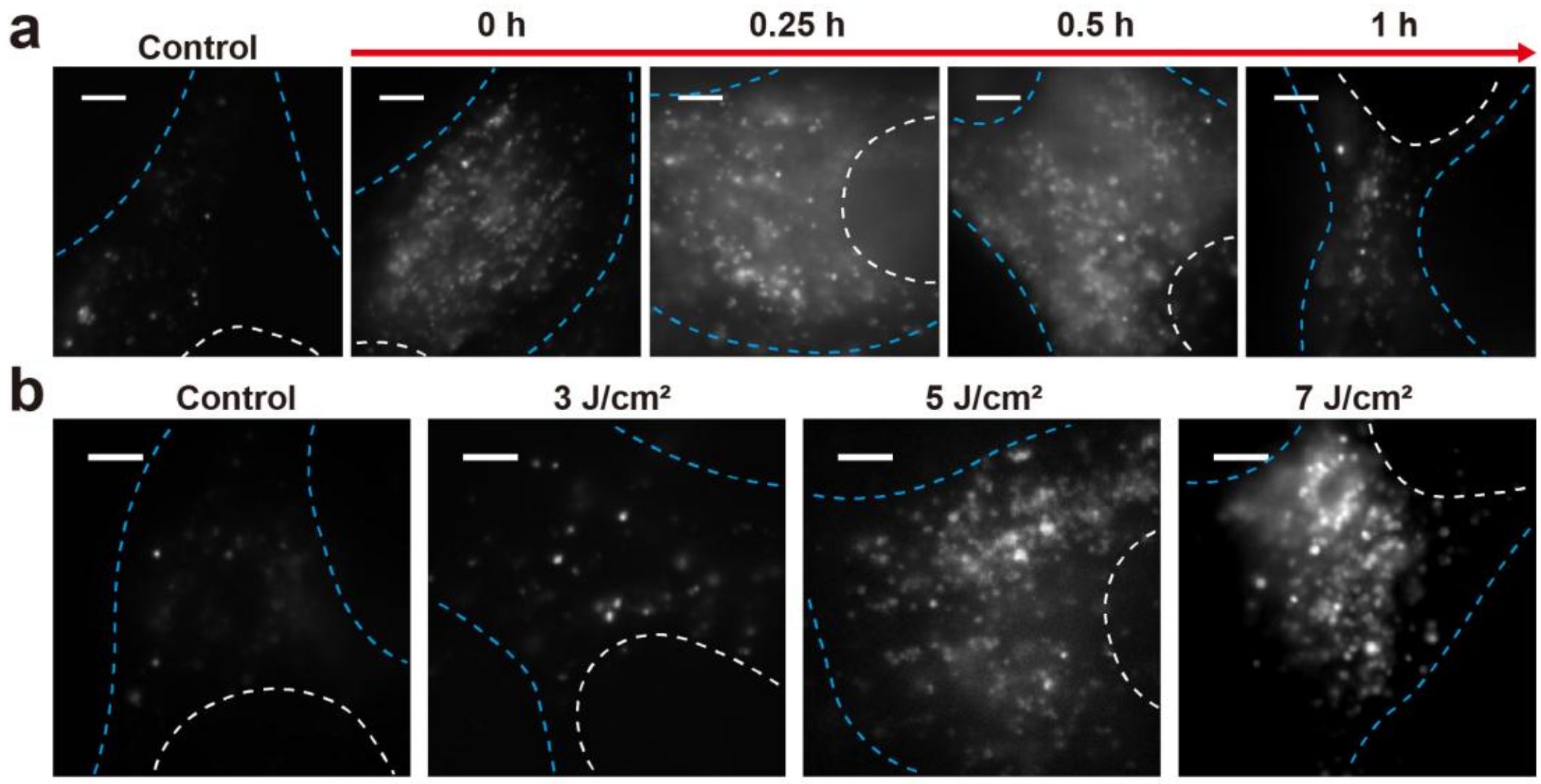

Fig. S13. Representative DCFH-DA fluorescence images showing intracellular ROS levels in HDF cells **(a)** at different time points post-UV illumination and **(b)** under varying UV doses. White dashed line: nuclear boundary. Blue dashed line: cell boundary. Scale bars, 5 μm.

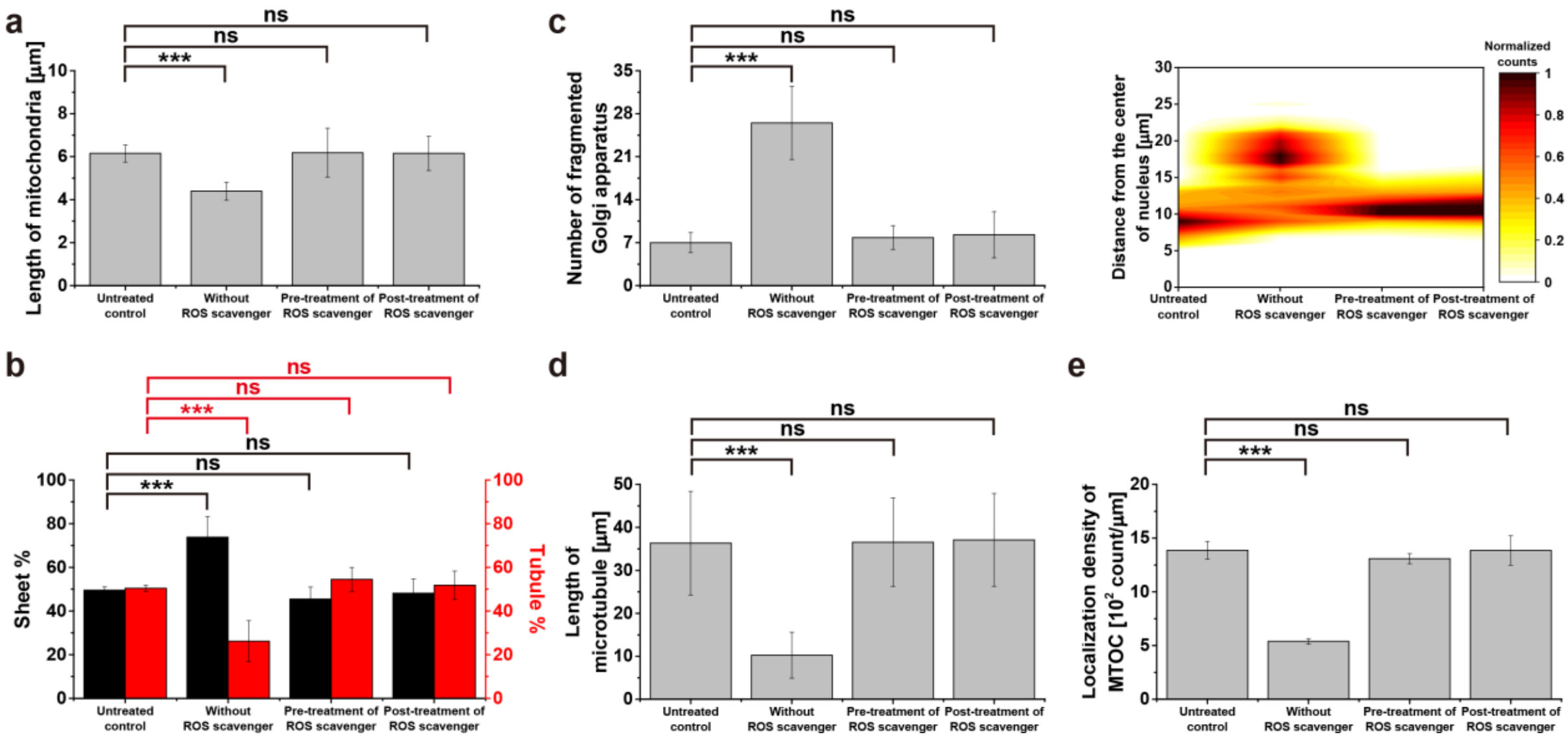

Fig. S14. Quantitative analyses of organelle morphology in UV-irradiated HDF cells with and without ascorbic acid treatment as a ROS scavenger. **(a)** Mitochondrial length, **(b)** relative proportions of tubular versus sheet-like ER structures, **(c)** number of Golgi fragments and distance of Golgi fragments from the nuclear centroid, **(d)** localization density of MTOC, and **(e)** microtubule length under four conditions: untreated control, UV-irradiated without ascorbic acid treatment, and UV-irradiated with either pre- or post-treatment with ascorbic acid. (n = 12, mean ± SEM) One-way ANOVA with Tukey's post hoc test, ns: $P > 0.05$, ***$P < 0.001$ compared to the control.

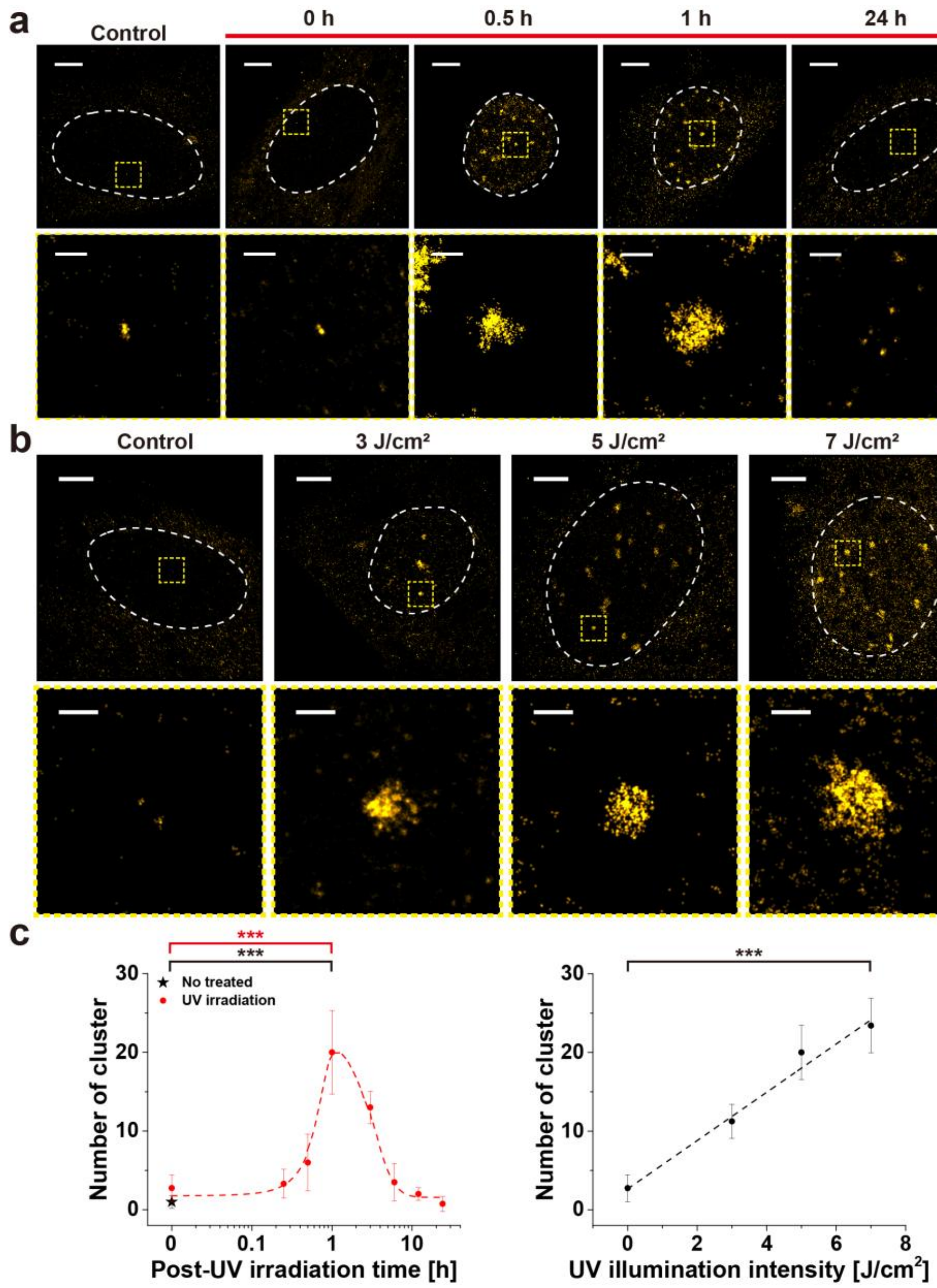

Fig. S15. Representative STORM images of γH2AX in HDF cells **(a)** at different post-UV illumination time points and **(b)** under varying UV doses. White dashed line: nuclear boundary. Scale bars, 5 μm in (a) and (b) and 500 nm in the yellow dashed box region in (a) and (b). **(c)** Quantitative analysis of γH2AX in HDF cells after UV irradiation, corresponding to the γH2AX cluster data presented in Fig. 3C. Left: Time course of number of γH2AX clusters at different post-irradiation time points. Right: Dependence of number of γH2AX clusters on UV illumination intensity, measured 1 h after exposure. Data are presented as mean ± SEM from twelve independent experiments. One-way ANOVA with Tukey's post hoc test, ****P* < 0.001 compared to the control.

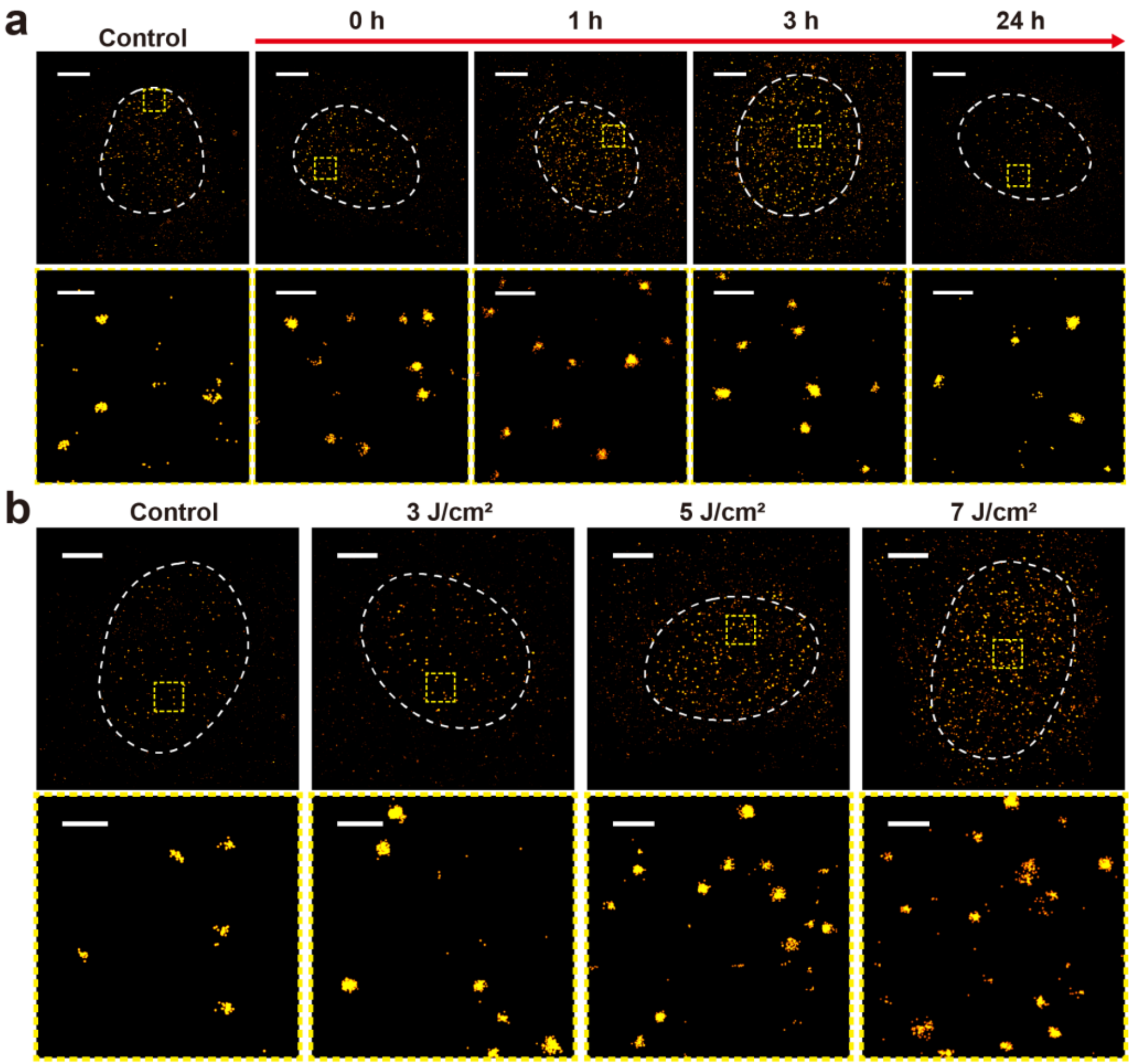

Fig. S16. Representative STORM images of PARP-1 in HDF cells **(a)** at different post-UV illumination time points and **(b)** under varying UV doses. White dashed line: nuclear boundary. Scale bars, 5 μm in (a) and (b) and 500 nm in the yellow dashed box region in (a) and (b).

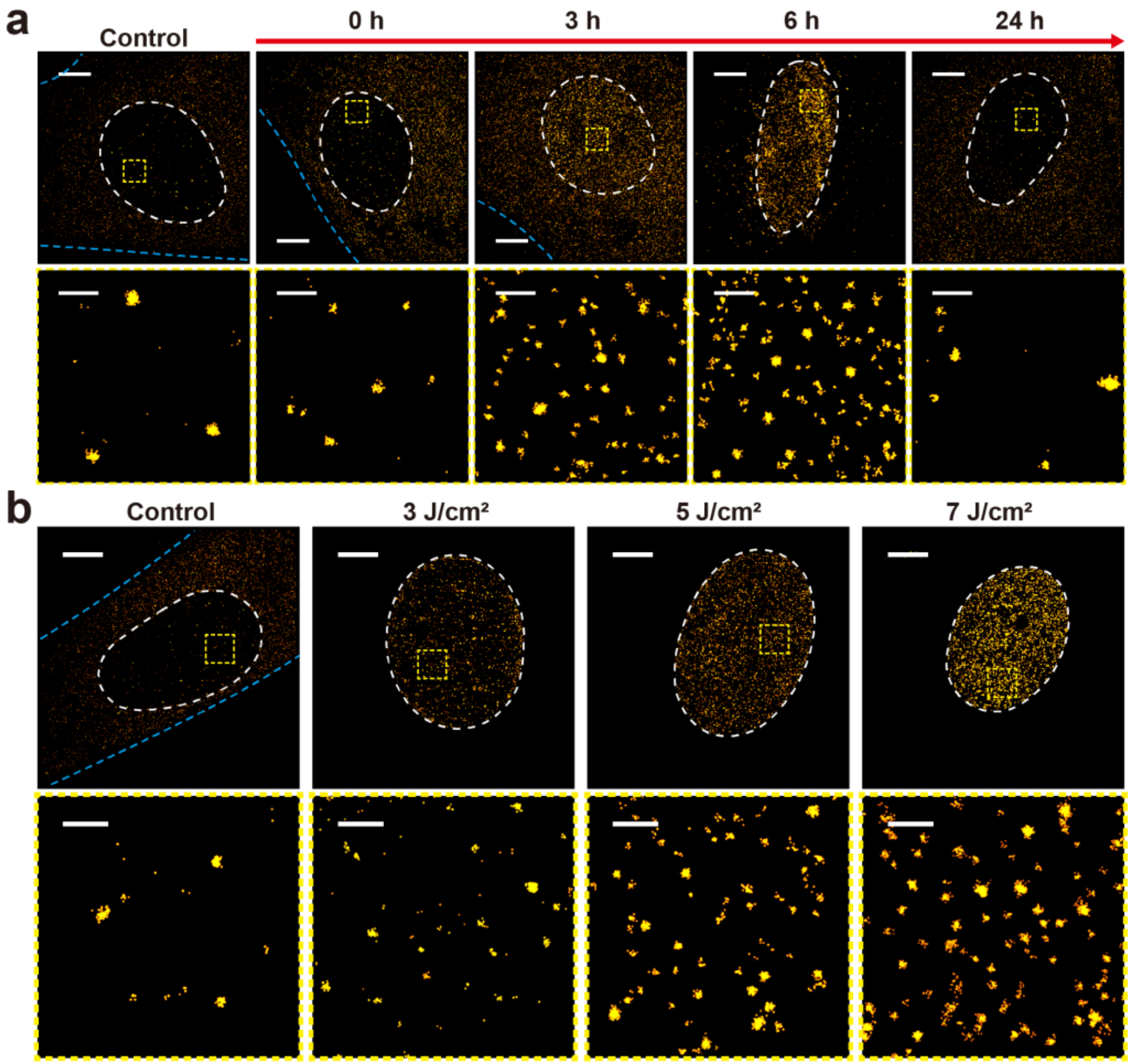

Fig. S17. Representative STORM images of NF-κB in HDF cells **(a)** at different post-UV illumination time points and **(b)** under varying UV doses. White dashed line: nuclear boundary. Blue dashed line: cell boundary. Scale bars, 5 μm in (a) and (b) and 500 nm in the yellow dashed box region in (a) and (b).

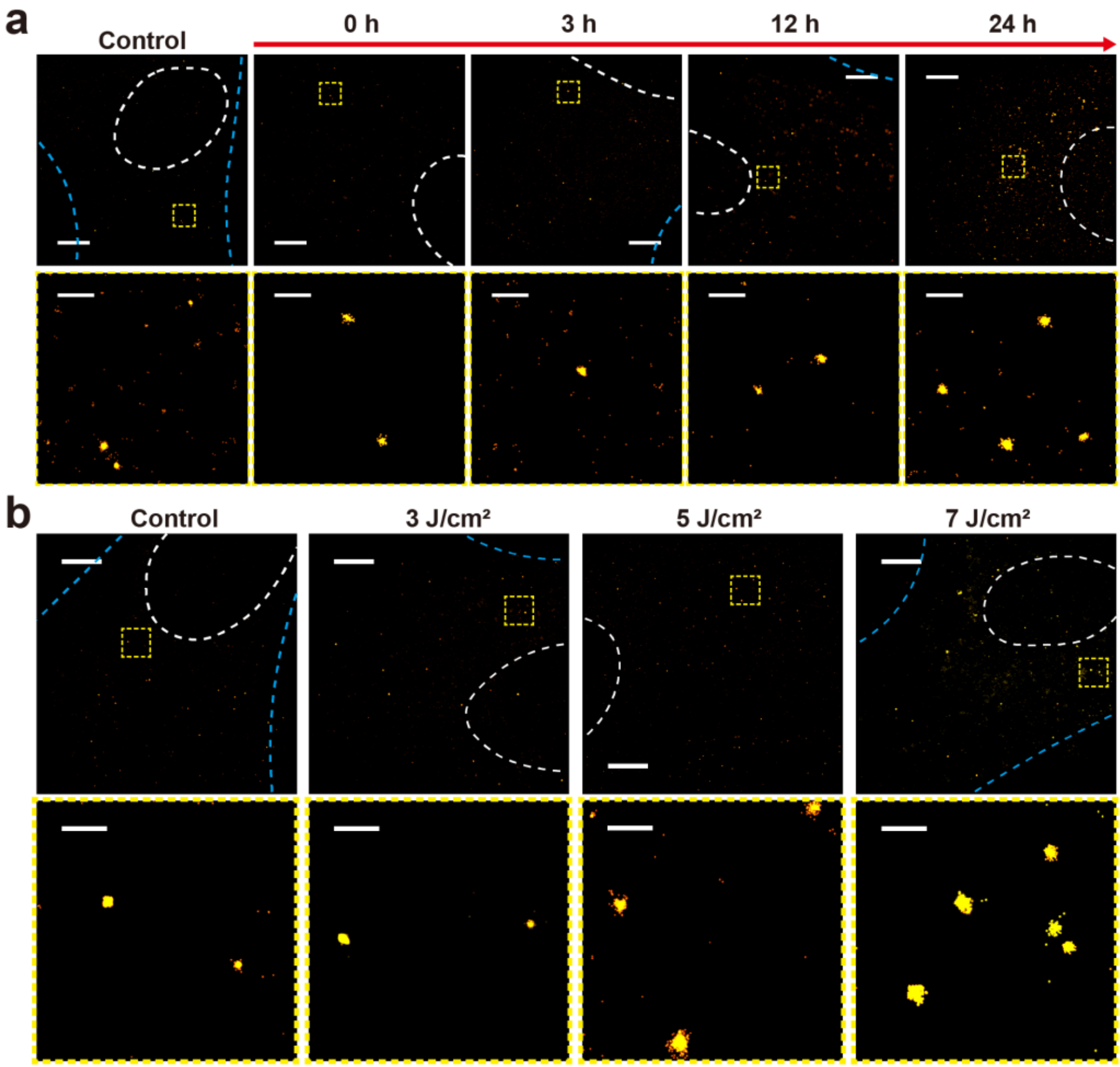

Fig. S18. Representative STORM images of IL-6 in HDF cells **(a)** at different post-UV illumination time points and **(b)** under varying UV doses. White dashed line: nuclear boundary. Blue dashed line: cell boundary. Scale bars, 5 μm in (a) and (b) and 500 nm in the yellow dashed box region in (a) and (b).

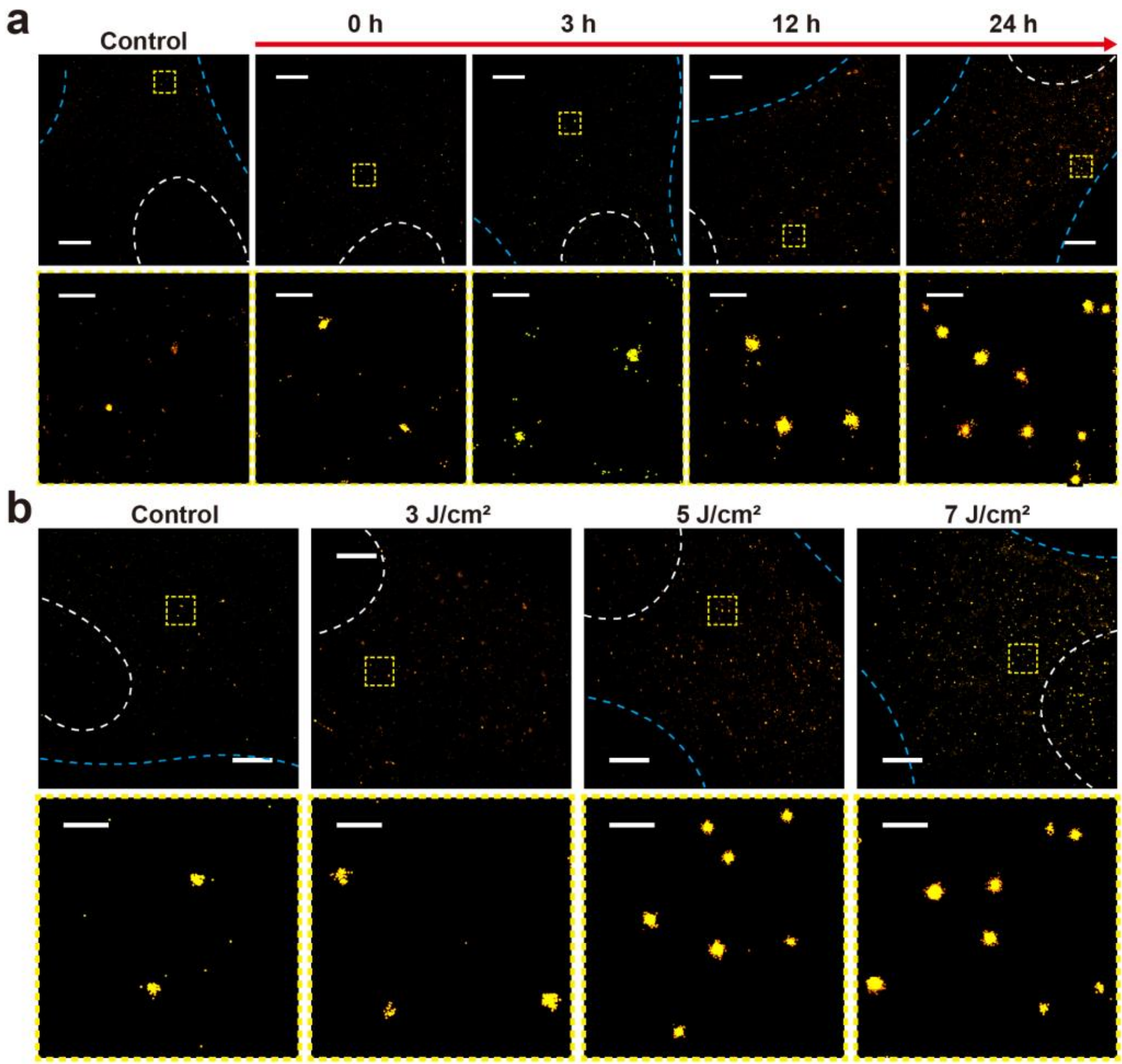

Fig. S19. Representative STORM images of IL-8 in HDF cells **(a)** at different post-UV illumination time points and **(b)** under varying UV doses. White dashed line: nuclear boundary. Blue dashed line: cell boundary. Scale bars, 5 μm in (a) and (b) and 500 nm in the yellow dashed box region in (a) and (b).

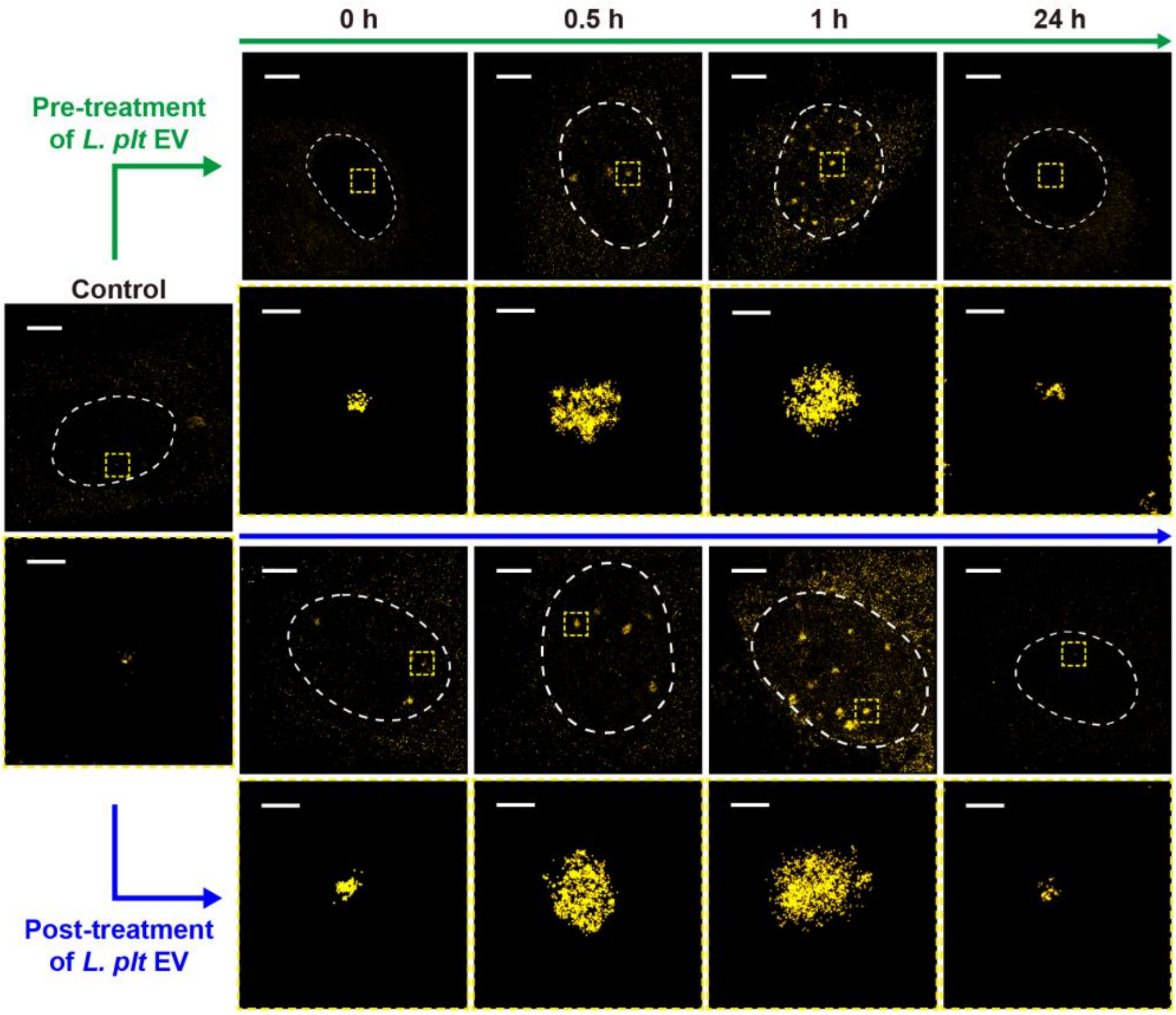

Fig. S20. Representative STORM images of γH2AX in HDF cells following UV irradiation with pre- and post-treatment with *L. plantarum* EVs. Representative STORM images of γH2AX at different post-UV irradiation time points in cells pretreated (upper) or post-treated (lower) with *L. plantarum* EVs, compared to UV-irradiated controls. White dashed line: nuclear boundary. Scale bars, 5 μm and 500 nm in the yellow dashed box region.

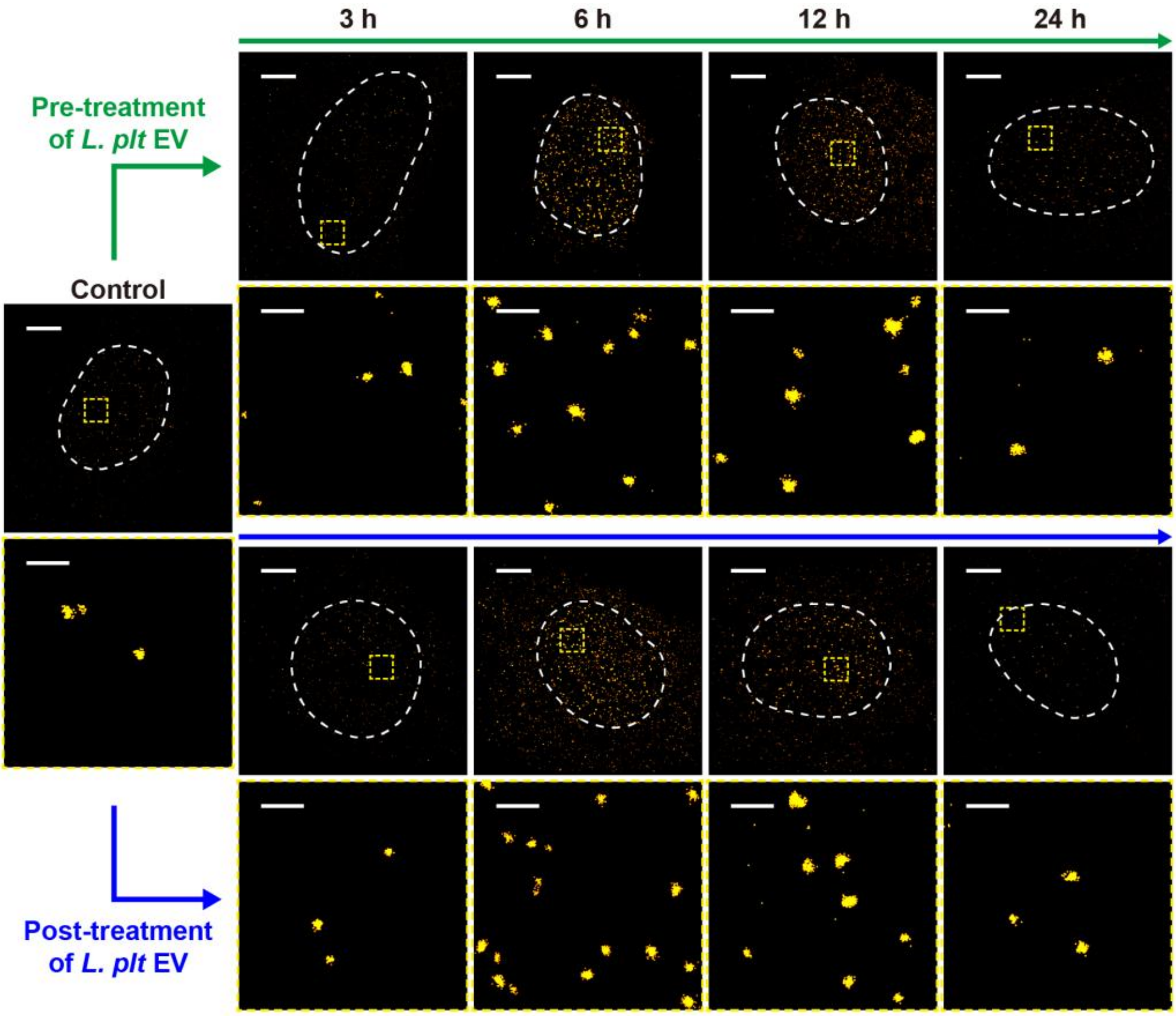

Fig. S21. Representative STORM images of PARP-1 in HDF cells following UV irradiation with pre- and post-treatment with *L. plantarum* EVs. Representative STORM images of PARP-1 at different post-UV irradiation time points in cells pretreated (upper) or post-treated (lower) with *L. plantarum* EVs, compared to UV-irradiated controls. The delayed peak of PARP-1 clustering at approximately 6 h in EV-treated cells, compared to approximately 3 h in UV-only cells, is consistent with the quantitative data presented in Fig. 4C. White dashed line: nuclear boundary. Scale bars, 5 μm and 500 nm in the yellow dashed box region.

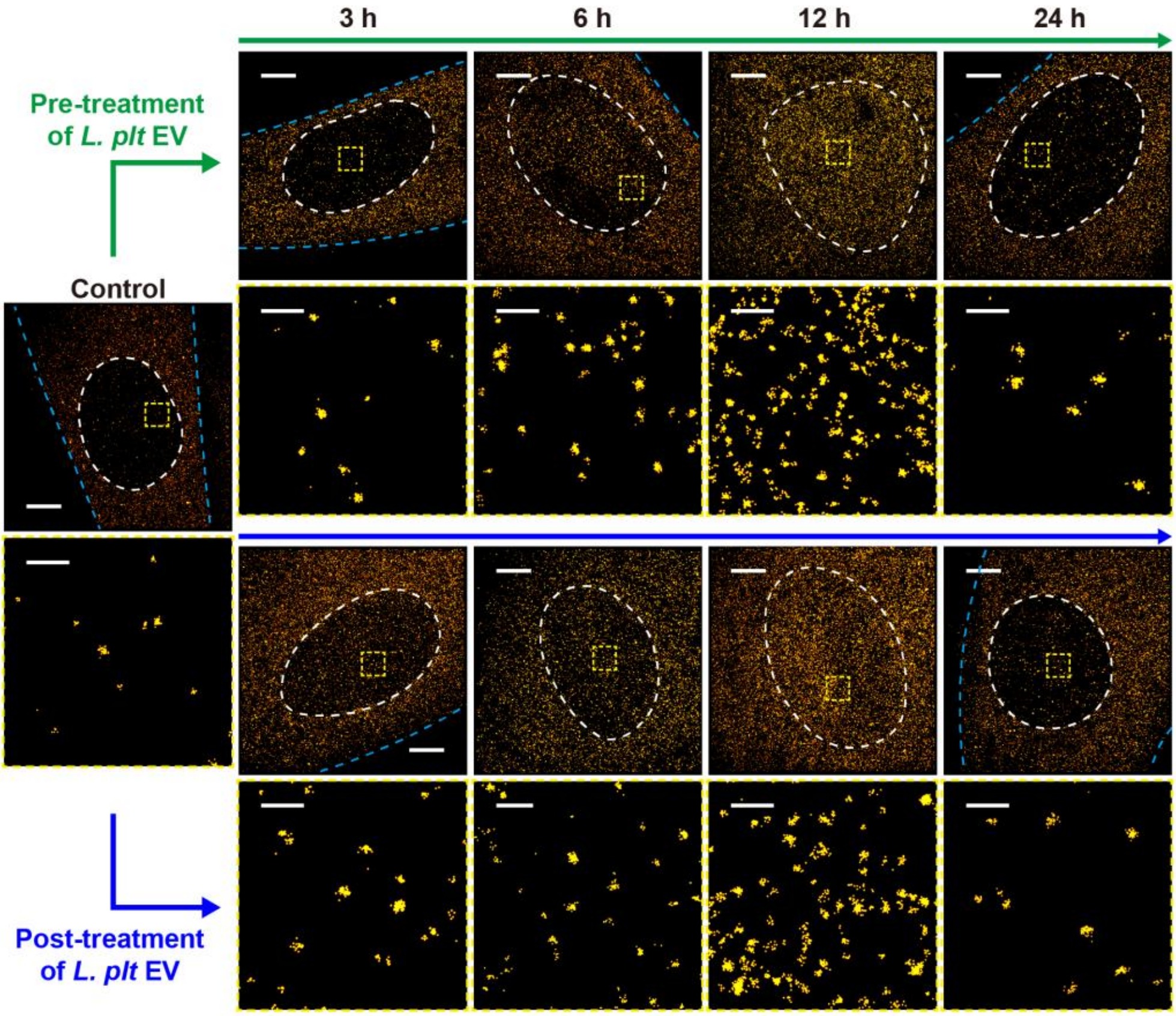

Fig. S22. Representative STORM images of NF-κB in HDF cells following UV irradiation with pre- and post-treatment with *L. plantarum* EVs. Representative STORM images of NF-κB at different post-UV irradiation time points in cells pretreated (upper) or post-treated (lower) with *L. plantarum* EVs, compared to UV-irradiated controls. The substantially reduced nuclear accumulation of NF-κB in EV-treated cells across all time points is consistent with the quantitative data presented in Fig. 4D. White dashed line: nuclear boundary. Blue dashed line: cell boundary. Scale bars, 5 μm and 500 nm in the yellow dashed box region.

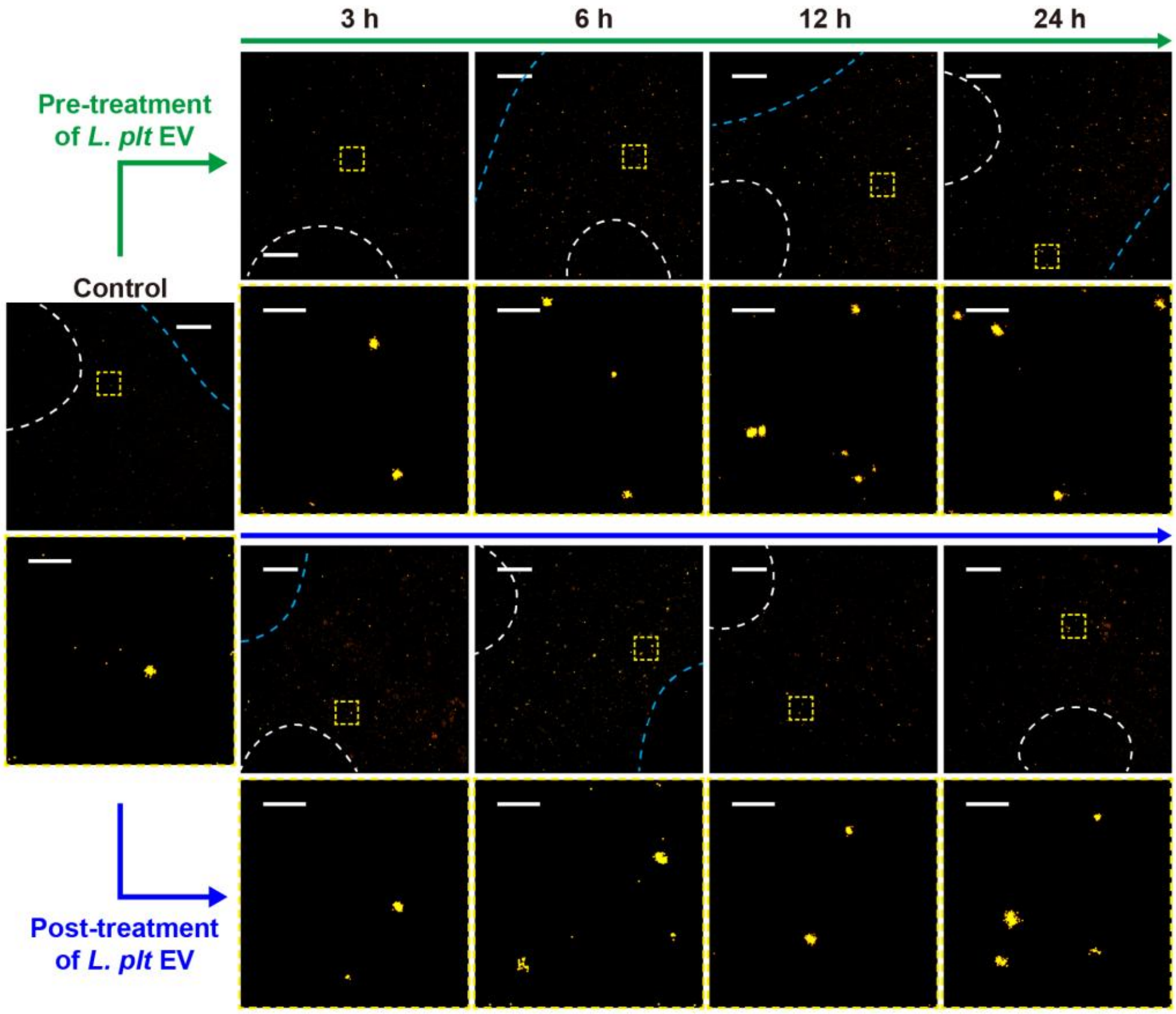

Fig. S23. Representative STORM images of IL-6 in HDF cells following UV irradiation with pre- and post-treatment with *L. plantarum* EVs. Representative STORM images of IL-6 at different post-UV irradiation time points in cells pretreated (upper) or post-treated (lower) with *L. plantarum* EVs, compared to UV-irradiated controls. The marked reduction in IL-6 cluster number in EV-treated cells relative to UV-only controls is consistent with the quantitative data presented in Fig. 4E. White dashed line: nuclear boundary. Blue dashed line: cell boundary. Scale bars, 5 µm and 500 nm in the yellow dashed box region.

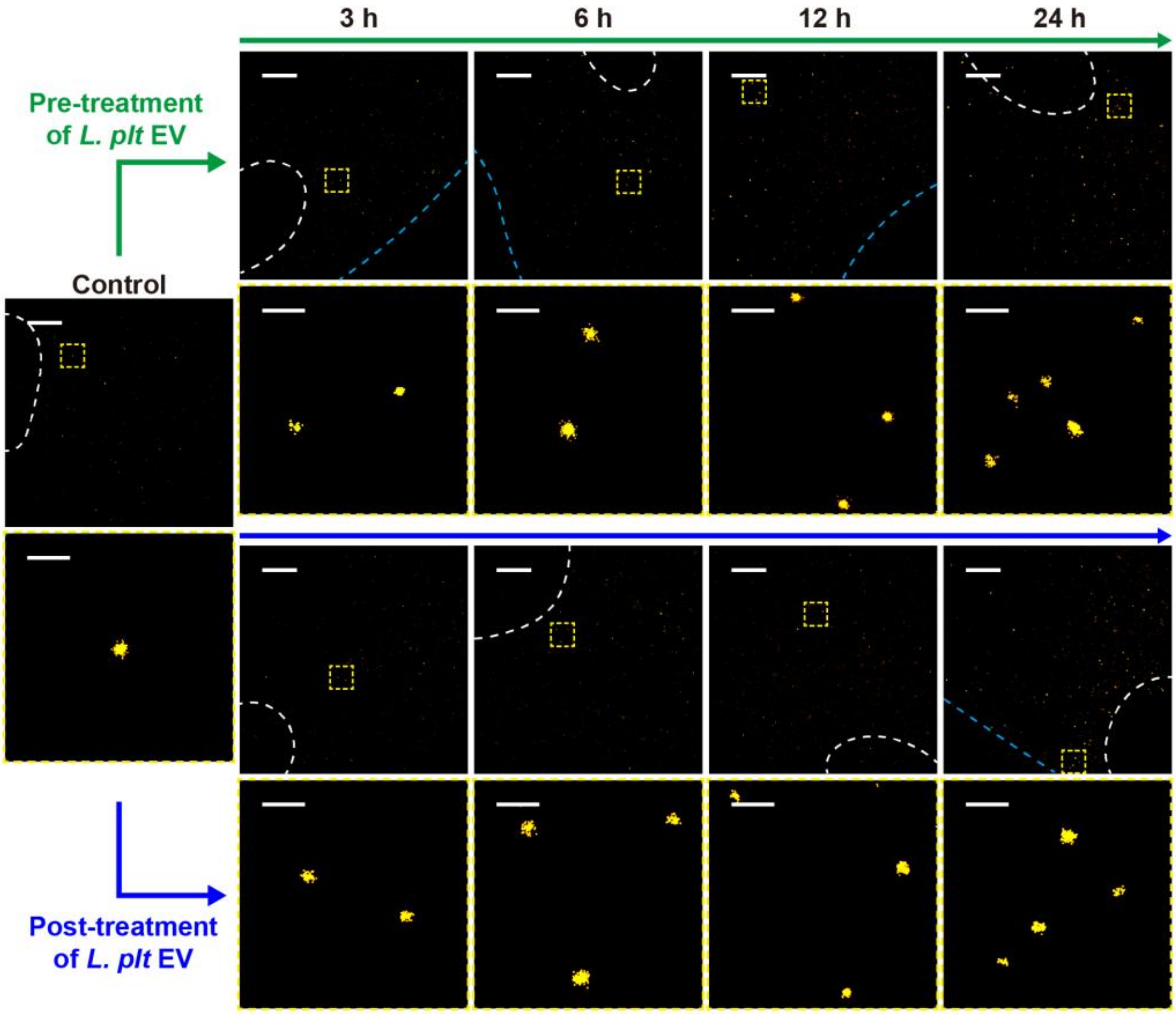

Fig. S24. Representative STORM images of IL-8 in HDF cells following UV irradiation with pre- and post-treatment with *L. plantarum* EVs. Representative STORM images of IL-8 at different post-UV irradiation time points in cells pretreated (upper) or post-treated (lower) with *L. plantarum* EVs, compared to UV-irradiated controls. The marked reduction in IL-8 cluster number in EV-treated cells relative to UV-only controls is consistent with the quantitative data presented in Fig. 4F. White dashed line: nuclear boundary. Blue dashed line: cell boundary. Scale bars, 5 µm and 500 nm in the yellow dashed box region.

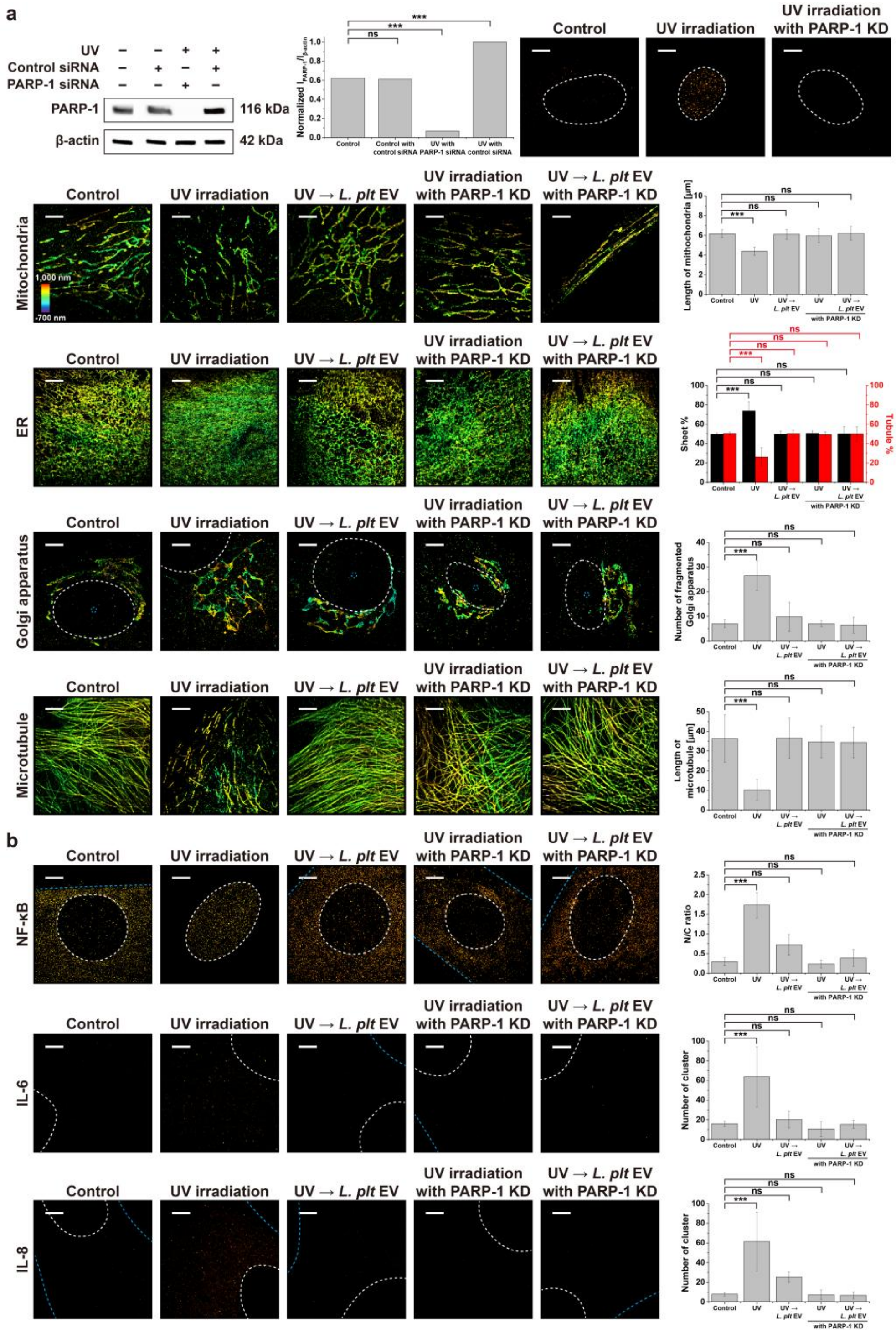

Fig. S25. Genetic perturbation of the PARP-1–NF-κB signaling axis in UV-irradiated HDF cells. **(a)** Representative STORM images and quantitative analyses of mitochondrial length, relative proportions of tubular and sheet-like ER structures, number of Golgi fragments, and microtubule length in HDF cells transfected with control siRNA or PARP-1-targeting siRNA, with or without *L. plantarum* EV treatment, following UV irradiation. Western blot and STORM images confirming PARP-1 knockdown efficiency are shown at the top. **(b)** Representative STORM images and quantitative analyses of NF-κB nuclear-to-cytoplasmic (N/C) localization density ratio and IL-6 and IL-8 cluster number under the same conditions as in (a). White dashed line: nuclear boundary. Blue dashed line: cell boundary. Scale bars, 5 μm. Data are presented as mean ± SEM from twelve independent experiments. One-way ANOVA with Tukey's post hoc test, ns: $P > 0.05$, $***P < 0.001$ compared to the control.

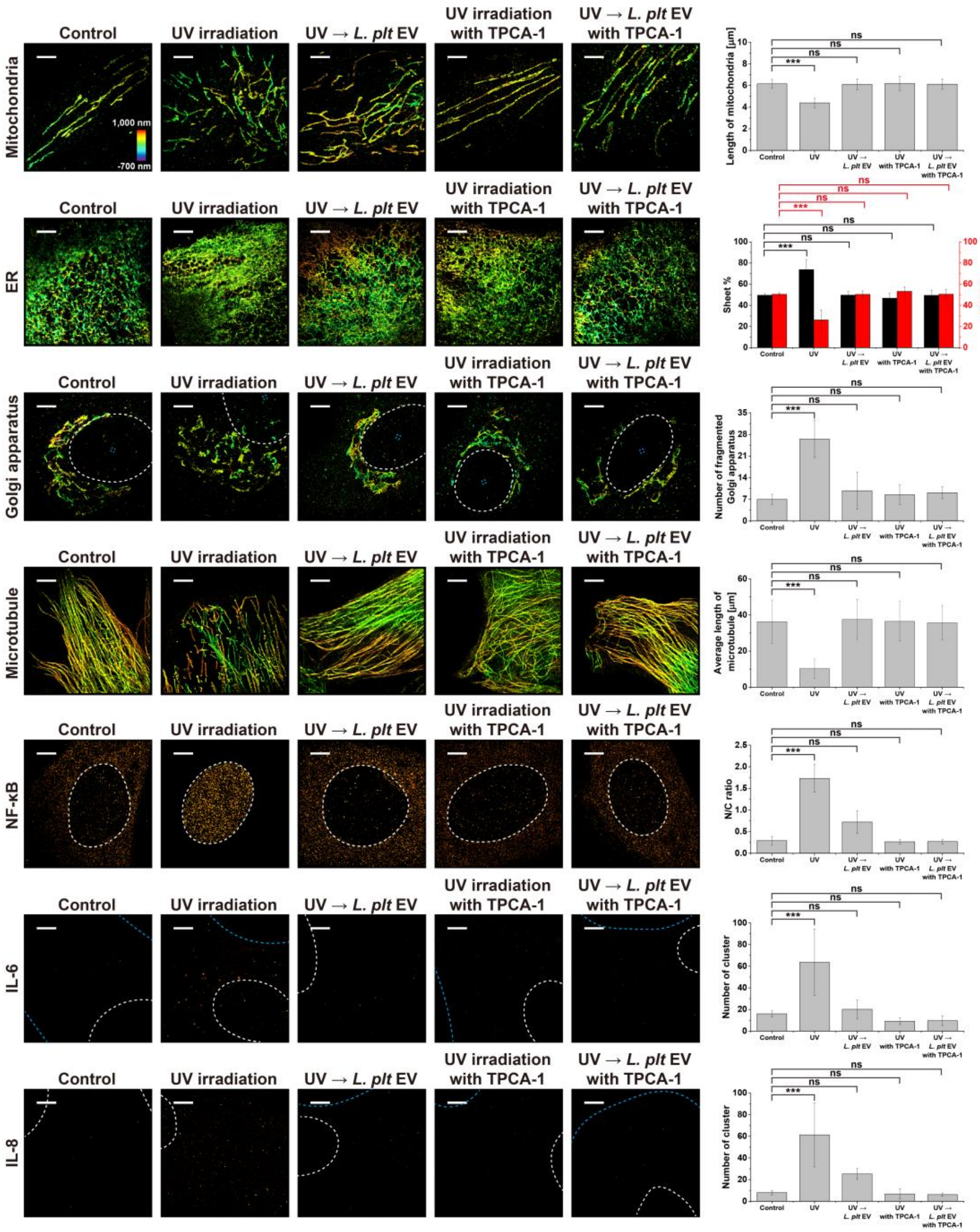

Fig. S26. Pharmacological perturbation of the PARP-1–NF-κB signaling axis in UV-irradiated HDF cells. Representative STORM images and quantitative analyses of mitochondrial length, relative proportions of tubular and sheet-like ER structures, number of Golgi fragments, microtubule length, and IL-6 and IL-8 cluster number in UV-irradiated HDF cells treated with the NF-κB inhibitor TPCA-1, with or without *L. plantarum* EV treatment. The control microtubule image is the same as that shown in Fig. 2E and is reproduced here for comparison. In all panels, NF-κB inhibition recapitulated the suppressive effects of *L. plantarum* EV treatment on both organelle ultrastructure and IL-6 and IL-8 clustering, and additional EV treatment produced no further suppression, confirming that EV-mediated protective effects are channeled through the PARP-1–NF-κB signaling axis. White dashed line: nuclear boundary. Blue dashed line: cell boundary. Scale bars, 5 μm. Data are presented as mean ± SEM from twelve independent experiments. One-way ANOVA with Tukey's post hoc test, ns: $P > 0.05$, $***P < 0.001$ compared to the control.

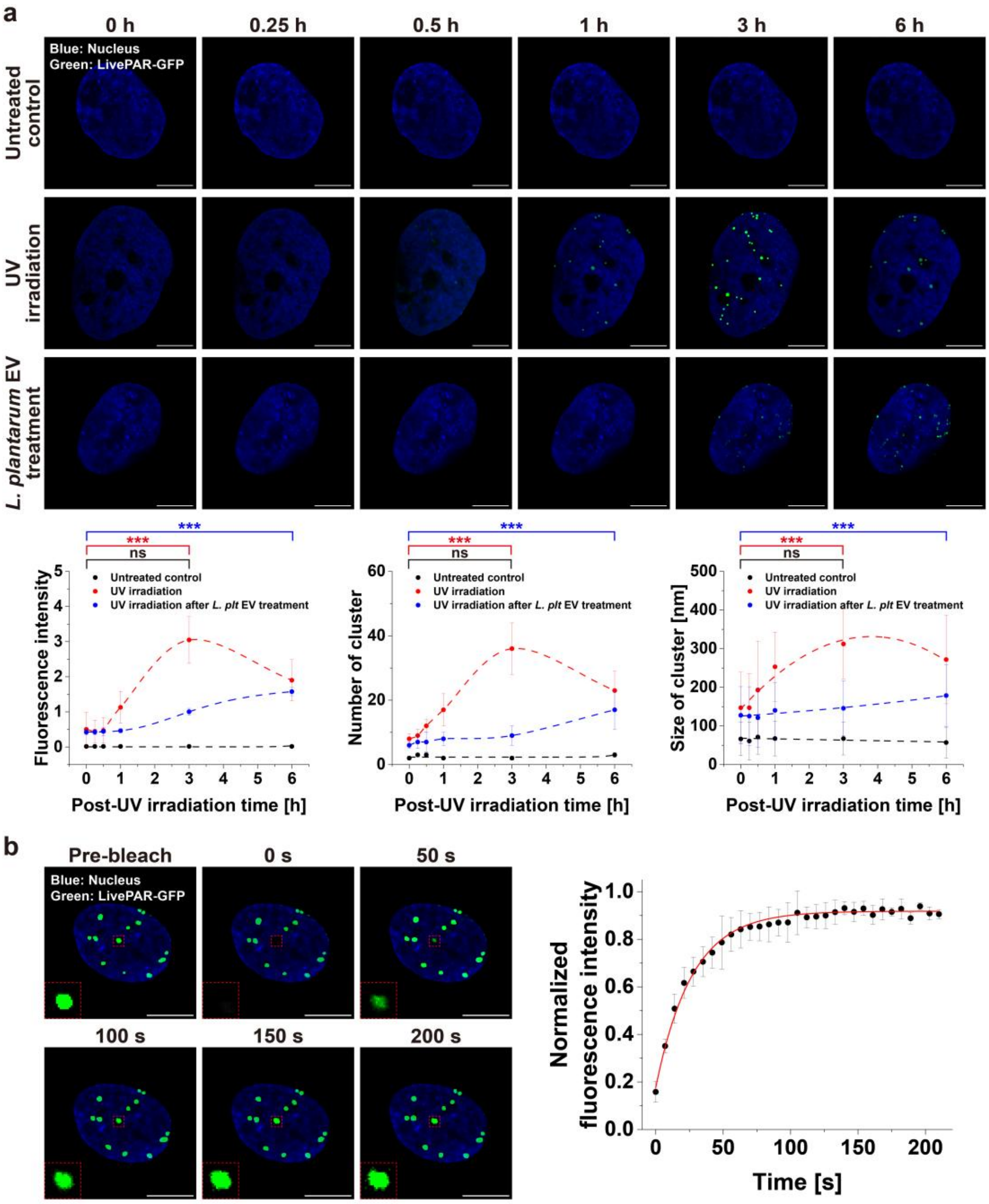

Fig. S27. Live-cell imaging and FRAP analysis of UV-induced PARP-1 condensate dynamics in HDF cells. **(a)** Representative time-lapse confocal images of LivePAR signal in UV-irradiated HDF cells without and with *L. plantarum* EV pre-treatment, showing the temporal progression of intranuclear PARP-1 activity clustering following UV exposure. Quantitative analysis of normalized nuclear LivePAR-GFP fluorescence intensity, number and size of clusters over time is shown below. *L. plantarum* EV pre-treatment delayed the onset and reduced the magnitude of UV-induced nuclear LivePAR enrichment compared to UV-only controls, consistent with the kinetic delay observed in time-resolved STORM imaging. **(b)** Representative FRAP images and recovery curves of LivePAR-positive nuclear foci in UV-irradiated HDF cells. Rapid and near-complete fluorescence recovery was observed following photobleaching of individual foci, indicating dynamic molecular exchange between the assemblies and the surrounding nucleoplasm and confirming the liquid-like nature of UV-induced PARP-1 condensates. Red dashed box: bleached region. Scale bars, 10 μm. Data are presented as mean ± SEM from twelve independent experiments. One-way ANOVA with Tukey's post hoc test, ns: $P > 0.05$, $^{***}P < 0.001$ compared to the control.
